# Identification of m6A-associated LncRNAs as predict factors for the immune infiltration and prognosis of thyroid cancer

**DOI:** 10.1080/07853890.2023.2192049

**Published:** 2023-03-28

**Authors:** Yongcheng Su, Beibei Xu, Jiangquan Li, Qianwen Shen, Ziyu Lei, Miaomiao Ma, Fuxing Zhang, Tianhui Hu

**Affiliations:** a Department of General Surgery, The First Hospital affiliated to Xiamen University, School of Medicine, Xiamen University, Xiamen, China; b Cancer Research Center, Xiamen University School of Medicine, Xiamen, China; c Shenzhen Research Institute of Xiamen University, Shenzhen, China

**Keywords:** m6A, thyroid cancer, immunotherapy, lncRNA

## Abstract

**Objective:**

This study aims to evaluate the prognostic value of m6A-associated long noncoding RNAs (lncRNAs) and their interaction with tumour microenvironment in thyroid cancer (THCA).

**Methods:**

The clinical and gene expression data of tumours from 502 patients with THCA and 58 adjacent normal tissues were retrieved from The Cancer Genome Atlas (TCGA)–THCA dataset. The Pearson test was utilized to identify potential m6A-associated lncRNAs (*p* < 0.001 and Pearson correlation coefficient > 0.4). Quantitative real-time polymerase chain reaction was performed to verify the expression levels of lncRNAs in tissues. MTT, EdU, colony formation and wound-healing assays were performed to determine the functions of m6A-associated lncRNAs in THCA cell proliferation and metastasis.

**Results:**

M6A-associated lncRNAs were identified in three cluster groups. A significant survival difference was found among them, with cluster 1 patients showing worse survival. Moreover, lower immune and estimate scores were correlated to poorer prognosis, and CD8+ T cell and memory CD4+ T cell levels were increased in cluster 1. Cluster 2, with better overall survival, had high expression of PD-L1 and CTLA-4. Eleven of the m6A-associated lncRNAs were screened to establish the risk model, including AC007365.1, AC008555.1, AC040160.1, AC064807.1, AC126773.4, AL023583.1, AL512306.2, EIF2AK3-DT, LINC00667, LYPLAL1-DT and MIR181A2HG. Based on the median risk score, THCA patients were stratified into low-risk and high-risk groups. Overall survival analysis showed a dramatic difference between the two groups. qRCR was performed to verify the expression levels of lncRNA (LYPLAL1-DT, EIF2AK3-DT and MIR181A2HG) in THCA and adjacent normal tissues. Furthermore, functional experiments showed that knockdown of MIR181A2HG obviously inhibited the proliferation and migration of papillary thyroid cancer (PTC) cells *in vitro*, whereas LYPLAL1-DT overexpression promoted PTC cell proliferation and migration.

**Conclusions:**

Eleven of the m6A-associated lncRNAs were identified as a risk model to predict clinical outcomes and provide a novel and efficient immunotherapeutic strategy for THCA patients.

**Key messages**
m6A-associated lncRNAs can be used to predict the clinical outcomes of thyroid cancer patients.
An m6A-associated lncRNAs risk model, which can accurately evaluate the immune status and risk stratification in individual thyroid cancer patients, was established.
Knockdown/overexpression of representative lncRNAs in the risk model significantly affected the proliferation and migration of papillary thyroid cancer cells.

## 1. Introduction

Thyroid cancer (THCA) is the most common endocrine malignancy worldwide, accounting for more than 586,202 new cancer cases and 43,646 deaths worldwide in 2020 [1]. Depending on histological differences, THCAs can be classified into differentiated thyroid carcinoma (DTC) and anaplastic thyroid carcinoma (ATC) [2]. DTCs, including thyroid, follicular and poorly DTCs, contribute to approximately 90% of all patients with THCA [3]. The ATC subtype is associated with rapid progression and poor prognosis, and no effective therapy is currently available for the ATC subtype [2]. Therefore, the identification of new and more innovative and ideal therapeutic targets for THCA is urgent.

Long noncoding RNAs (lncRNAs) are a class of RNAs of more than 200 nucleotides in length that do not code for a protein [4–9], previously considered ‘junk’ [10]. The rapid evolution in high-throughput biome sequencing technologies has allowed an increasing number of potential functions of lncRNAs to be discovered [11]. Moreover, an increasing amount of research has shown that lncRNAs play a key role in various physiological and pathological processes, including cancer initiation and progression [12,13] and regulation of immune responses [14,15]. For instance, lncRNA can activate the Wnt/β-catenin signaling pathway by interacting directly with β-catenin, subsequently promoting colon cancer epithelial-to-mesenchymal transition and metastasis [16]. Interest in the potential function of lncRNAs is increasing, and various types of studies on lncRNAs are rapidly emerging [17].

N6‐methyladenosine (m6A) modification is a type of eukaryotic RNA modification [18] that has been shown to play a regulatory role in numerous human diseases, especially in cancer initiation and progression [19], such as in lung [20], endometrial [21] and liver cancers [22]. It is reported that the upregulation of the m6A regulatory gene METTL14 contributes to pancreatic cancer metastasis [23]. LNCAROD can promote cancer progression through m6A methylation mediated by METTL3 and METTL14 in patients with head and neck squamous cell carcinoma [24]. Similarly, METTL3, a vital m6A methyltransferase, regulates the m6A modification of LINC00958 and affects the prognosis of patients with liver cancer [25]. In addition, recent studies have also shown that the m6A modification is related to immunoregulation [18, 26–29]. However, the underlying mechanism of how m6A-associated lncRNAs are involved in tumour regulation through immune infiltration in THCA remains unclear.

To date, few studies have attempted to explore the association between m6A modification and the tumour immune response in THCA. We attempted to evaluate the prognostic value of m6A-associated lncRNAs in THCA. Subsequently, consensus clustering groups and risk scores were generated to further assess the relationship between m6A-associated lncRNAs and the tumour microenvironment (TME), including immune checkpoints, tumour immune cell infiltration and immune scores in THCA patients.

## 2. Materials and methods

### 2.1. Thyroid cancer samples collection

Fourteen pairs of thyroid cancer and the adjacent normal tissue samples were collected from the first affiliated hospital of Xiamen University; all thyroid cancer samples were confirmed by pathological examination. This study was approved by the Institutional Review Board of the first affiliated hospital of Xiamen University and conducted with the informed consent of all patients.

### 2.2. Cell lines, cell culture and cell transfection

Papillary thyroid cancer cell lines (KTC-1 and BCBAP) were obtained from the Chinese Academy of Sciences (Shanghai, China) Cell Bank of Type Culture Collection. KTC-1 and BCBAP cells were grown in 1640 with 10% fetal bovine serum (FBS) and cultured at 37 °C in a 5% CO_2_ incubator. The LYPLAL1-DT overexpression and short hairpin RNA (shRNA) of MIR181A2HG were induced with lentiviral expression system. All sequence information is presented in Supplementary Table S1.

### 2.3. Cell proliferation assay

Cell proliferation was measured using the methyl thiazolyl tetrazoliym (MTT) assays, the MTT assays were performed following the manufacturer’s instructions. Approximately 3000 PTC cells were incubated for 24, 48, 72 and 96 h in 96-well plates, and 20 l of MTT ((5 mg/ml, Sigma-Aldrich; Merck KGaA) was added to each well for 4 h until the purple precipitate was fully yielded. Absorbance at 490 nm was measured in 15 min after 150 l DMSO was added into each well to dissolve the precipitates.

### 2.4. EdU cell proliferation assays

EdU assay was used to detect the DNA synthesis of growing PTC cells by using the EdU imaging kit (RiboBio, China), and the EdU assay steps were performed following the manufacturer’s instructions.

### 2.5. Colony formation assays

Approximately 500 cells per well were seeded into a 6-well culture plate and incubated at 37 °C for 2 weeks. After washing with PBS twice, cells were fixed with 4% paraformaldehyde for 15 min and then dyed with crystal violet. Only colonies ≥ 50 cells were counted under a microscope. Each experiment was repeated three times.

### 2.6. Wound healing

Wound-healing assays were performed according to previously described methods [30].

### 2.7. Quantitative real-time polymerase chain reaction

According to the manufacturer’s instructions, total RNA was extracted with the Trizol (Invitrogen) reagent. 1 μg of mRNA was reversely transcribed to cDNA by the reverse transcription system (AG11711, Accurate Biology, Hunan, China). qPCR amplification was subsequently performed with the SYBR Master Mix (YEASEN Biotech, Shanghai, China). The GAPDH gene level was used as reference. The primers for quantitative real-time polymerase chain reaction (qRCR) amplification were listed in Supplementary Table S2.

### 2.8. Bioinformatic analysis

The clinical and gene expression data (FPKM value) of tumours from 502 patients with THCA and 58 adjacent normal tissues were retrieved from The Cancer Genome Atlas (TCGA)–THCA dataset. Moreover, the expression profiles of 23 m6A regulatory genes in TC patients, including writers (METTL3, METTL14, METTL16, WTAP, VIRMA, ZC3H13, RBM15 and RBM15B), readers (YTHDC1, YTHDC2, YTHDF1, YTHDF2, YTHDF3, HNRNPC, FMR1, LRPPRC, HNRNPA2B1, IGFBP1, IGFBP2, IGFBP3 and RBMX) and erasers (FTO and ALKBH5), were extracted from the TCGA–THCA database.

The Pearson test was utilized to identify potential m6A-associated lncRNAs (*p* < 0.001 and Pearson correlation coefficient > 0.4). Subsequently, m6A-associated prognostic lncRNAs in THCA were distinguished using univariate Cox regression analysis. The ‘ConsensusClusterPlus’ package in R was applied to perform cluster analysis [31]. The proportion of 22 human immune cell types in THCA patients was evaluated using the CIBERSORT algorithm, and the ESTIMATE algorithm, which included Immunoscore, ESTIMATE and stromal scores, was used to estimate the immune score in THCA patients [32]. An R script of the ESTIMATE algorithm was downloaded from http://bioinformatics.mdanderson.org/estimate. Higher ImmunoScores or StromalScores indicate greater levels of immune or stromal components in the TME. The ESTIMATE score is the sum of the ImmuneScore and StromalScore, and denotes the overall proportion of immune and stromal components in the TME. The formula for risk score was as follows:

$$\text{risk}\;\;\text{score}=\overset{n}{\underset{i=1}{\sum }}\text{expression}\;\;\text{of}\;\;\ln\;\;cRNAn*coeffi\text{ci}ent\;\ln\;cRNAn$$

The TCGA–THCA dataset wa s randomly divided into a training dataset (50%) and a test dataset (50%). A LASSO analysis was performed to identify lncRNAs with prognostic value for THCA patients.

### 2.9. Statistical analyses

Statistical analysis was performed using R studio (version 4.1.1), the R packages ‘survminer’ and ‘survival’ were used to perform survival analysis, and the significance between groups was assessed using the log-rank test. Receiver operating characteristic (ROC) curves were applied to assess the risk score. All experiments were representative of three independent experiments. And the data (mean ± SD) were evaluated by GraphPad Prism version 8.0.2. Differences between two groups were analysed by Student’s *t* test and differences among multiple groups by one-way ANOVA. *p* Values less than 0.05 were considered statistically significant.

## 3. Results

### 3.1. Identification of m6A-associated lncRNAs in THCA

**Figure 1.**
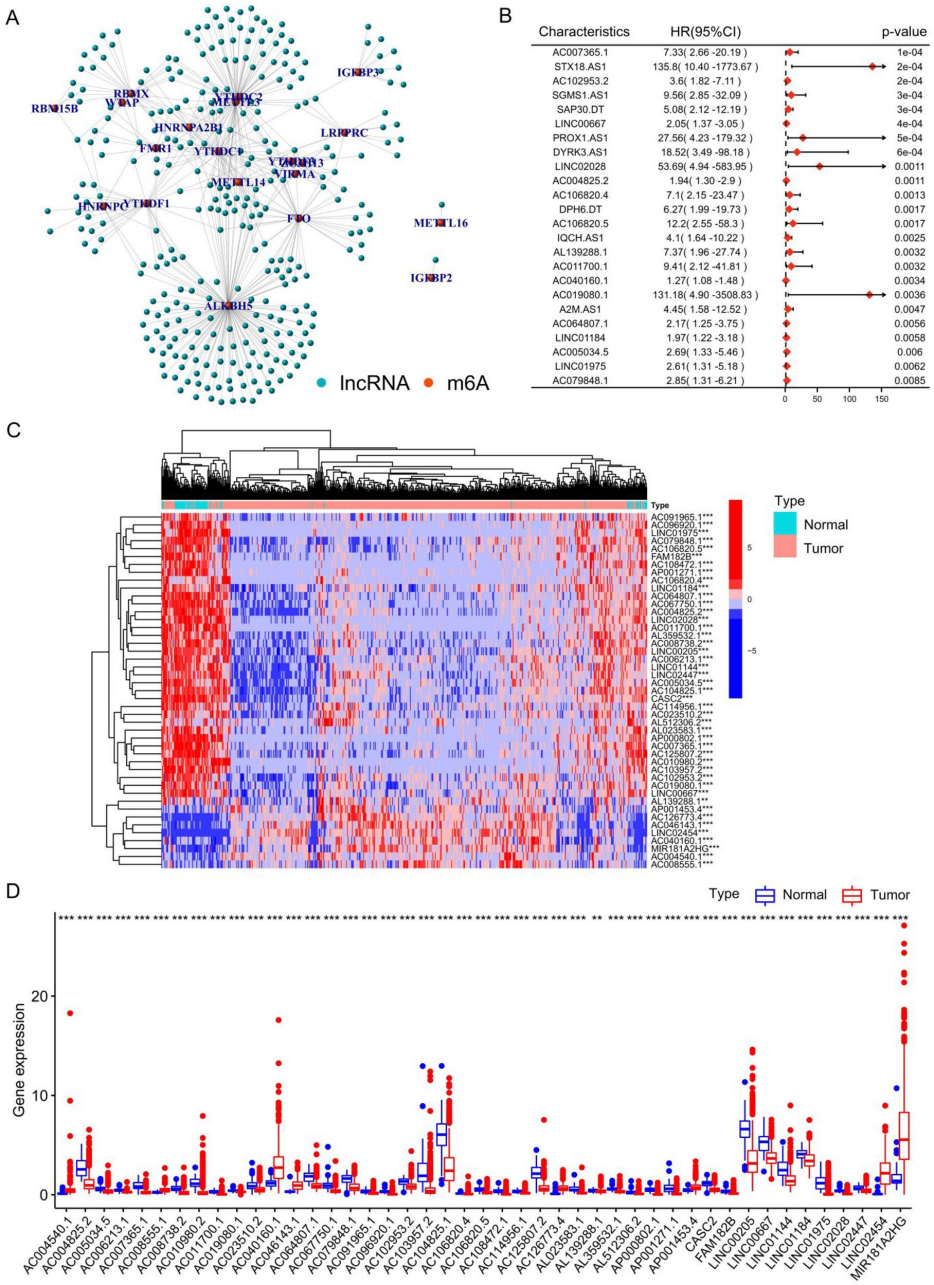
Identification of N6-methyladenosine (m6A)-associated long noncoding RNAs (lncRNAs) in thyroid cancer (THCA). (A) m6A-lncRNA co-expression network in THCA patients. (B) Forest plot of m6A-associated prognostic lncRNAs in THCA. (C) Heatmap of m6A-associated prognostic lncRNAs in THCA. (D) The expression levels of m6A-associated prognostic lncRNAs between normal and cancer tissues. *p < 0.05; **p < 0.01; ***p < 0.001.

We extracted and analysed the expression levels of 23 m6A regulatory factors in 502 THCA patients. Pearson correlation analysis was utilized to identify potential m6A-associated lncRNAs based on the TCGA–THCA cohort, when the Pearson correlation coefficient (R) was < 0.4 and *p* value was < 0.001, the lncRNA was defined as m6A-associated lncRNA. Ultimately, 322 lncRNAs were proposed to be m6A-associated lncRNAs (Supplementary Table S3), and the m6A regulator genes and m6A-associated lncRNA co-expression network was built based on Pearson correlation coefficients (Figure 1(A)). According to the univariate Cox analysis, 70 m6A-associated lncRNAs were closely correlated to the overall survival (OS) of THCA patients (Table S4). Figure 1(B) shows a forest plot of m6A-associated lncRNAs according to univariate Cox risk regression analysis, a lncRNA with HR > 1 was considered a risk lncRNA, and a gene with HR < 1 was considered a protective lncRNA. The heatmap (Figure 1(C)) and box plot (Figure 1(D)) display the differentially expressed m6A-associated lncRNAs in normal and THCA tissues. This indicates significant differences in the expression of m6A-associated lncRNAs between normal and cancer tissues and that these differences are correlated with the prognosis of patients with thyroid cancer.

### 3.2. Consensus clustering of m6A-associated lncRNAs in THCA

**Figure 2.**
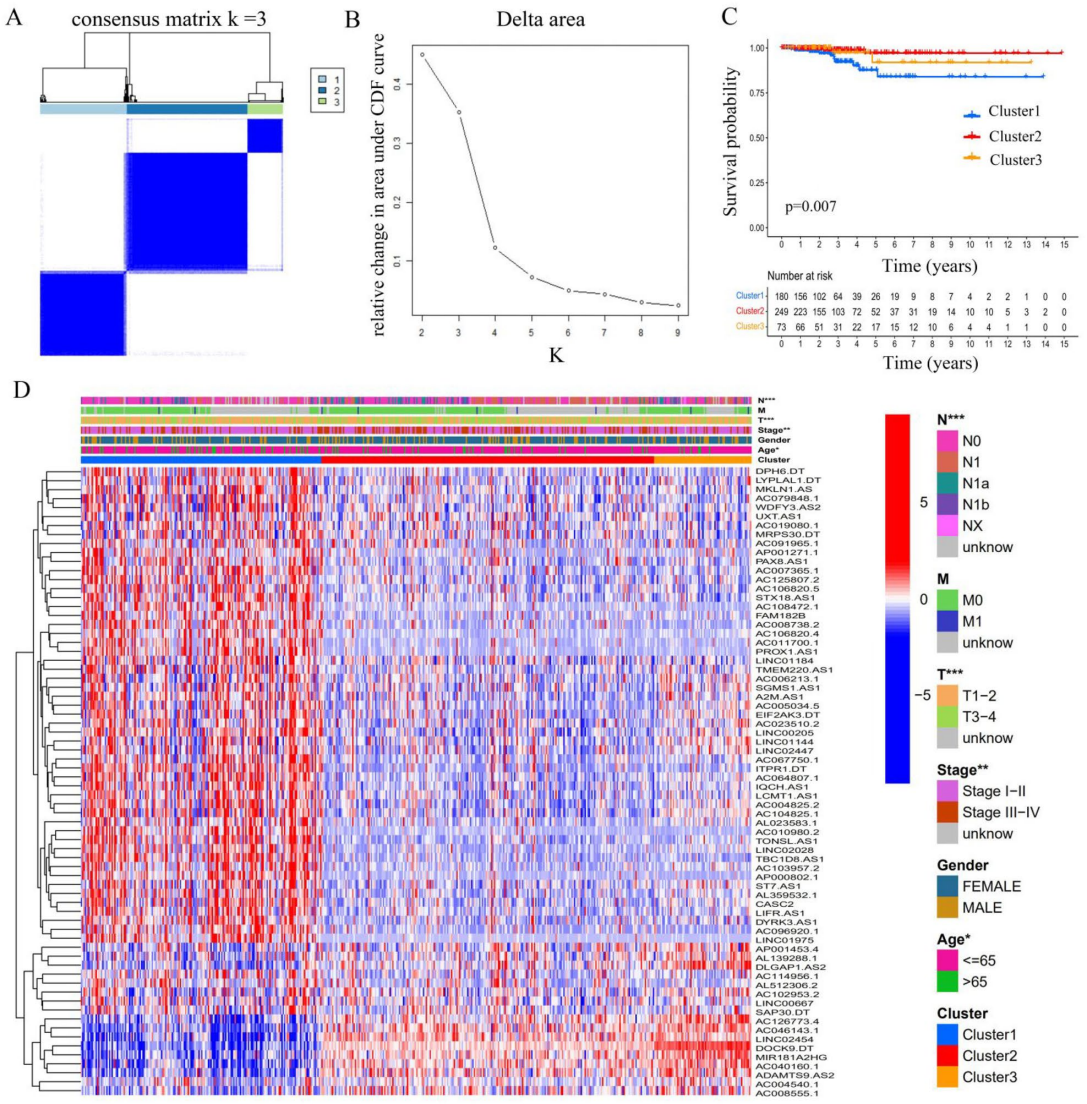
Consensus clustering of m6A-associated lncRNAs in THCA. (A) Consensus clustering matrix for *k* = 3. (B) The relative change in area under cumulative distribution function curve for *k* = 2 to 9. (C) Kaplan–Meier curves of overall survival for the three clusters in THCA. (D) Heatmap depicting the clinicopathologic features among the three clusters.

The ‘ConsensusClusterPlus’ package in R was used to perform cluster analysis [31], which demonstrated that clustering with *k* = 3 resulted in the greatest stability. When *k* = 3, the consensus matrix was divided into three distinct clusters (Figure 2(A,B)). Subsequently, 502 THCA patients were assigned to three different clusters according to their expression of 70 m6A-associated prognostic lncRNAs: cluster1 (*n* = 180), cluster 2 (*n* = 249), and cluster 3 (*n* = 73). For details regarding cluster comparisons (see Supplementary Table S5). Survival analysis revealed differences in survival among the clusters, with patients in cluster 1 having poorer prognoses than the other clusters (Figure 2(C)). Notably, higher expression levels of m6A-associated lncRNAs were observed in cluster 1 (Figure 2(D)), suggesting that higher levels of m6A-associated lncRNAs predict poorer prognosis. And the upper bar of the heatmap also showed the distribution of clinicopathological features among three clusters, including TMN stage, age, gender and stage.

### 3.3. Immune characteristics among the three clusters in THCA patients

**Figure 3.**
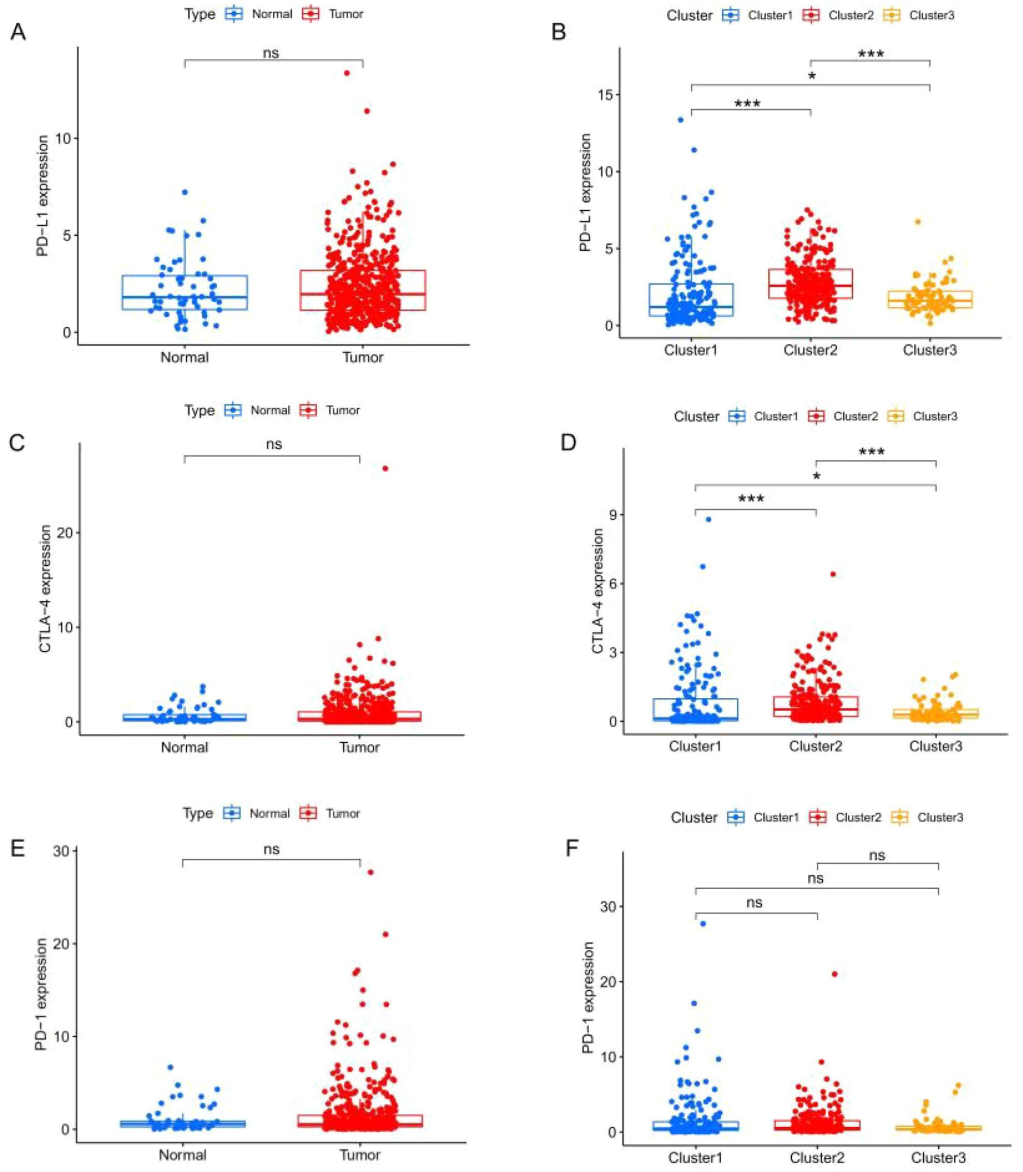
Immune characteristics among the three clusters. (A) Programmed death-ligand 1 (PD-L1) expression in THCA based on The Cancer Genome Atlas (TCGA) database. (B) The expression level of PD-L1 in three clusters based on the TCGA database. (C). Cytotoxic lymphocyte associated antigen-4 (CTLA-4) expression in THCA based on the TCGA database. (D) The expression level of CTLA-4 in the three clusters based on the TCGA database. (E) Programmed cell death protein 1 (PD-1) expression in THCA based on the TCGA database. (F) The expression level of PD-1 in the three clusters based on the TCGA database. ns: not significant, *p < 0.05; **p < 0.01; ***p < 0.001.

**Figure 4.**
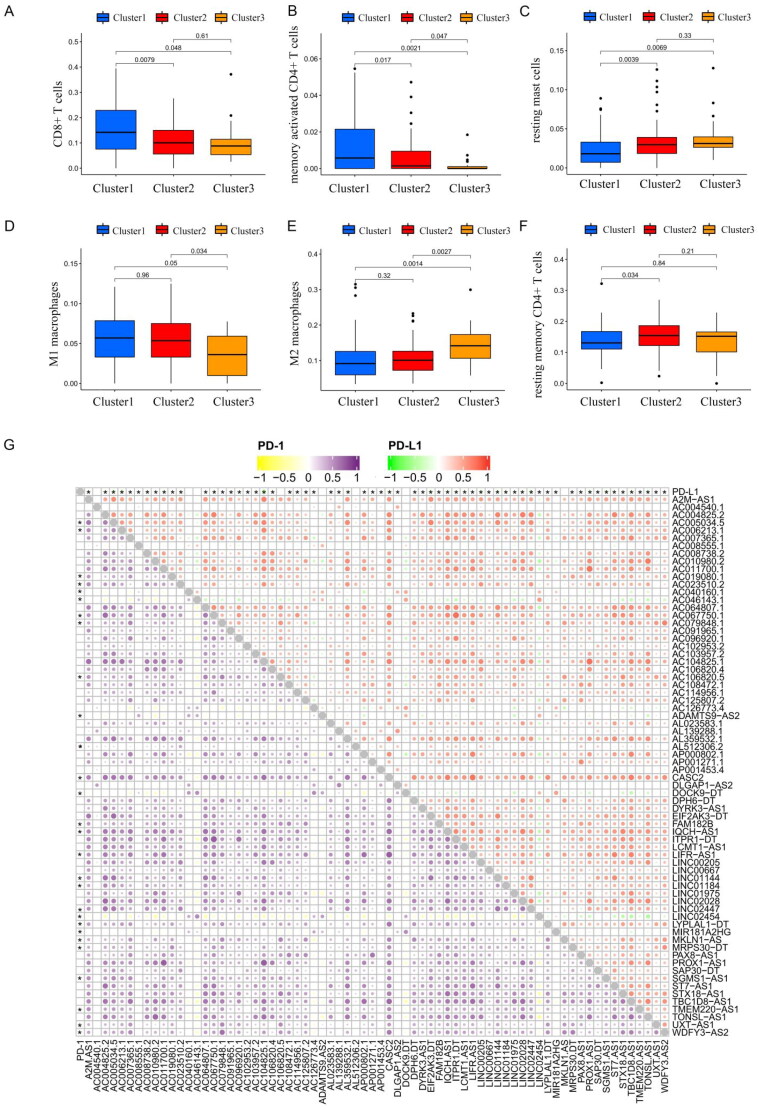
The numbers of CD8+ T cells (A), memory-activated CD4+ T cells (B), resting mast cells (C), M1 macrophages (D), M2 macrophages (E), and resting memory CD4+ T cells (F) in the three clusters based on TCGA database. Correlation analysis between expression of programmed death-ligand 1 (PD-L1) and N6-methyladenosine (m6A)-associated prognostic long noncoding RNAs (lncRNAs) based on the Pearson coefficient (G). Correlation analysis between levels of programmed cell death protein 1 (PD-1) and programmed death-ligand 1 (PD-L1) and m6A-associated lncRNAs based on the Pearson correlation coefficient. The upper right corner depicts the correlation between PD-L1 and m6A-associated lncRNAs in THCA, and the color of the dots represents the type of correlation (purple indicates a positive correlation and yellow indicates a negative correlation). The lower left corner depicts the correlation between PD-1 and m6A-associated lncRNAs in THCA, and the color of the dots represents the type of correlation (red indicates a positive correlation and green indicates a negative correlation). The size of the dots represents the magnitude of the correlation, with larger dots representing a stronger correlation. *p < 0.05.

Previous studies have shown that treatment with immunotherapy and chemotherapy can be influenced by the expression of immune checkpoint proteins. Therefore, we first explored the differential expression of immune checkpoint genes (CTLA-4, PD-1 and PD-L1). There was no significant difference between cancer tissue and the adjacent healthy tissue (Figure 3(A,C,E)), whereas cluster 2 had a high level of expression of PD-L1 (Figure 3(B)) and CTLA-4 (Figure 3(D)). The CIBERSORT algorithm, a deconvolution approach to assess the level of immune cell infiltration, was used to assess the different immune cell infiltration levels among various clusters (Figure 4). The box plot showed that the proportion of CD8+ T cells (Figure 4(A)) and memory CD4+ T cells (Figure 4(B)) in cluster 1 were higher, whereas resting mast cell levels were significantly lower in clusters 2 and 3 (Figure 4(C)). As for macrophages, we found that the levels of M1 macrophages were lower in cluster 3 (Figure 4(D)), and M2 macrophages had higher levels in cluster 3 (Figure 4(E)). However, the expression of resting memory CD4+ T cells was not significantly different among the various clusters (Figure 4(F)). Together, these results suggest that the immune microenvironment plays an important role in the occurrence and progression of THCA and is closely related to the prognosis of THCA patients.

Subsequently, a correlation analysis of immune checkpoint genes (PD-1/PD-L1) with m6A-associated lncRNAs was also performed based on Person correlation analysis, the results indicated that PD-L1 and PD-1 expression were found to have a significantly negative association with m6A-associated prognostic lncRNAs (Figure 4(G) and Supplementary Table S6).

**Figure 5.**
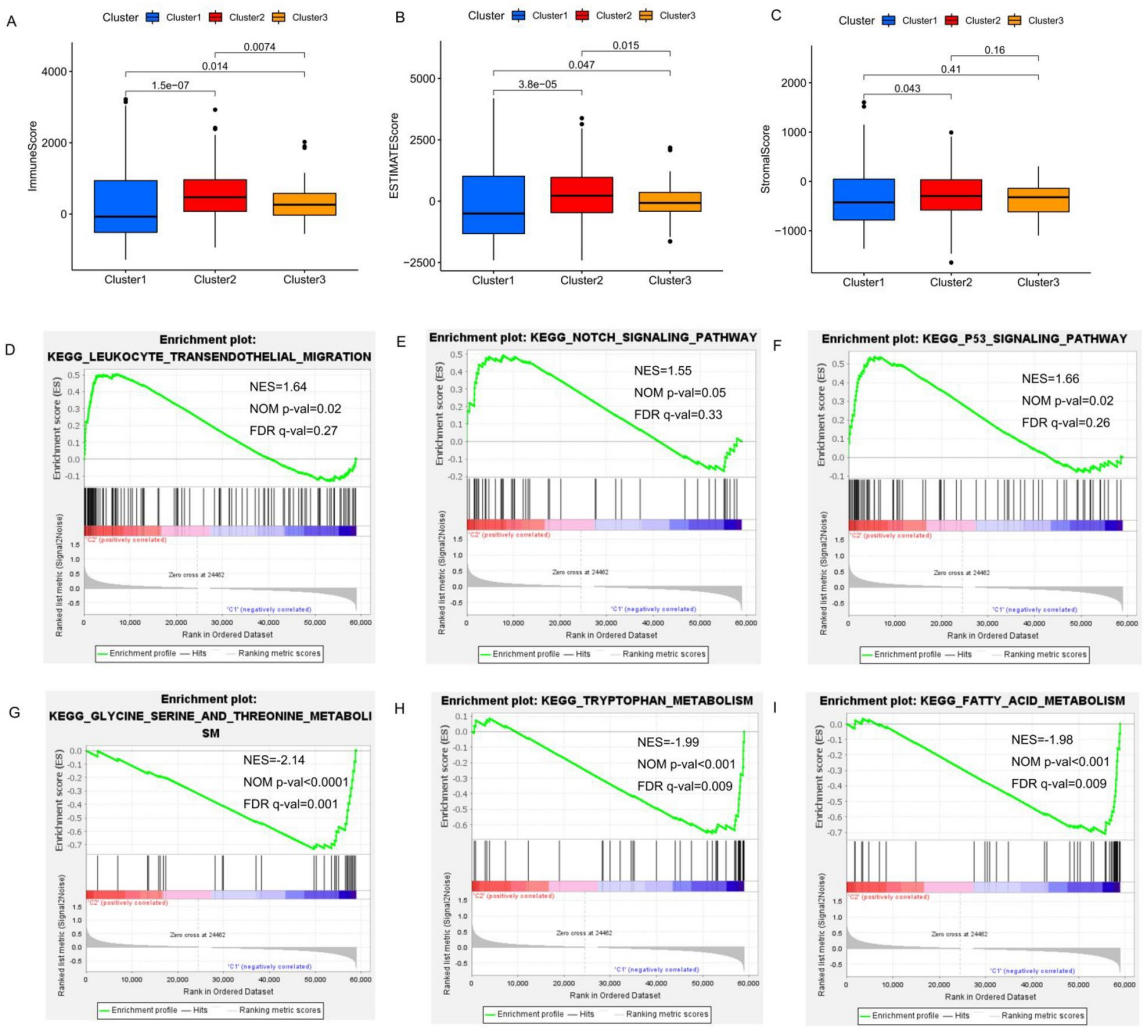
Immunoscore (A), ESTIMATE score (B), and stromal score (C) in clusters 1, 2 and 3. Gene set enrichment analysis of the N6-methyladenosine-associated long noncoding RNAs in cluster 2 (D–F) and cluster 1 (G–I), respectively. NES: normalized enrichment score; NOM *p* value: normalized *p* value; FDR: false discovery rate.

The ESTIMATE algorithm is used to infer the level of infiltrating stromal and immune cells in tumour tissues and tumour purity using gene expression data. The predictive ability of this method has been validated in large and independent data sets. As shown in Figure 5(A–C), significant differences in the immune and ESTIMATE scores were observed among three clusters; the immune score and ESTIMATE score in cluster 2 are clearly higher than those in clusters 1 and 3. In addition, the OS of patients in cluster 2 with higher immune and ESTIMATE scores was greater than that of clusters 1 and 3 (Figure 2(C), *p* = 0.007). Overall, cluster 2 was characterized by high TME scores and higher levels of immune checkpoint proteins (PD-L1 and CTLA-4), suggesting that THCA patients in cluster 2 are more likely to respond to immune checkpoint inhibition than patients in the other clusters.

Additionally, gene set enrichment analysis (GSEA) indicated that cancer hallmarks, including leukocyte transendothelial migration genes (Figure 5(D)), Notch signaling pathways (Figure 5(E) and p53 signaling pathways (Figure 5(F)) were more strongly correlated with cluster 2. Glycine, serine and threonine metabolism (NES = −2.140, false discovery rate [FDR] *q*-value = 4.64E − 04, Figure 5(G), tryptophan metabolism (NES = −1.99, FDR *q*-value = 0.009, Figure 5(H)) and fatty acid metabolism (NES = −1.98, FDR *q*-value = 0.009, Figure 5(I)) signaling pathways were enriched in cluster 1. These results suggest that m6A-associated lncRNAs may play an important role in regulating cancer-related pathways.

### 3.4. Construction of the m6A-associated lncRNA risk model in THCA patients

**Figure 6.**
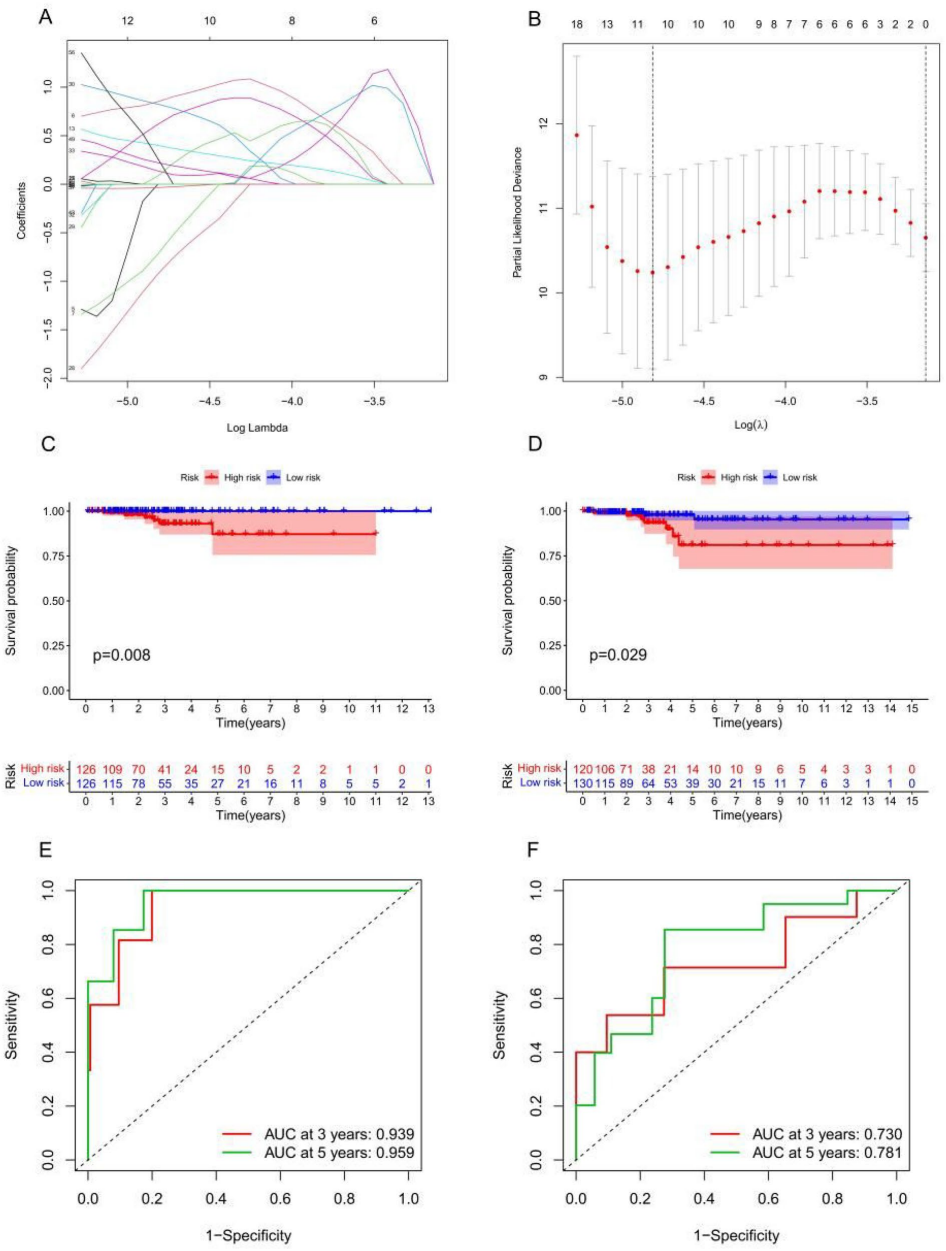
Risk model from m6A-associated lncRNAs. (A–B) LASSO coefficient of m6A-associated lncRNAs in thyroid cancer (THCA). Survival analysis for THCA patients in the training (C) and testing cohorts (D). The receiver operating characteristic curve of the risk model in the training (E) and testing cohorts (F).

According to the univariate Cox analysis, 70 m6A-associated lncRNAs were strongly correlated with the OS of THCA patients. Subsequently, LASSO regression analysis [33] selected 11 of the 70 m6A-associated lncRNAs for use in establishing a risk model to accurately evaluate risk stratification in individual samples (Figure 6(A,B)). These 11 lncRNAs included AC007365.1, AC008555.1, AC040160.1, AC064807.1, AC126773.4, AL023583.1, AL512306.2, EIF2AK3-DT, LINC00667, LYPLAL1-DT and MIR181A2HG. Risk scores were calculated using the following formula: risk score = AL023583.1 × 0.82 + AC007365.1 × 0.85 − AC008555.1 × 0.72 + AC040160.1 × 0.40 + AC064807.1 × 0.62 − AC126773.4 × 0.92 + AL023583.1 × 0.82 + AL512306.2 × 0.14 + EIF2AK3-DT × 0.08 + LINC00667 × 0.22 + LYPLAL1-DT × 0.26 − MIR181A2HG × 0.03. The median risk score was used to divide samples into low-risk and high-risk groups. Subsequently, the OS analysis revealed a dramatic difference between the high- and low-risk groups (Figure 6(C)) and testing datasets (Figure 6(D)). Supplementary Figure S1 shows the risk model distribution, survival status and heatmap of 11 m6A-associated prognostic lncRNAs in the training (Supplementary Figure S1(A)) and testing cohorts (Supplementary Figure S1(B)). The ROC analysis demonstrated that our risk model had a high prognostic value for THCA patients, the 3- and 5-year area under the curve (Figure 6(E–F)) in the training and testing datasets showed an excellent predictive value.

### 3.5. Risk model score as an independent prognostic factor in THCA patients

We first performed univariate Cox regression and found that the risk score was correlated to OS in THCA patients (Supplementary Figure S2(A)), we then conducted a multivariate analysis, which further confirmed the results of the univariate Cox regression analysis (Supplementary Figure S2(B)).

### 3.6. Association of the risk model score with age, N stage, clusters 1 and 2 and immunoscore in THCA

**Figure 7.**
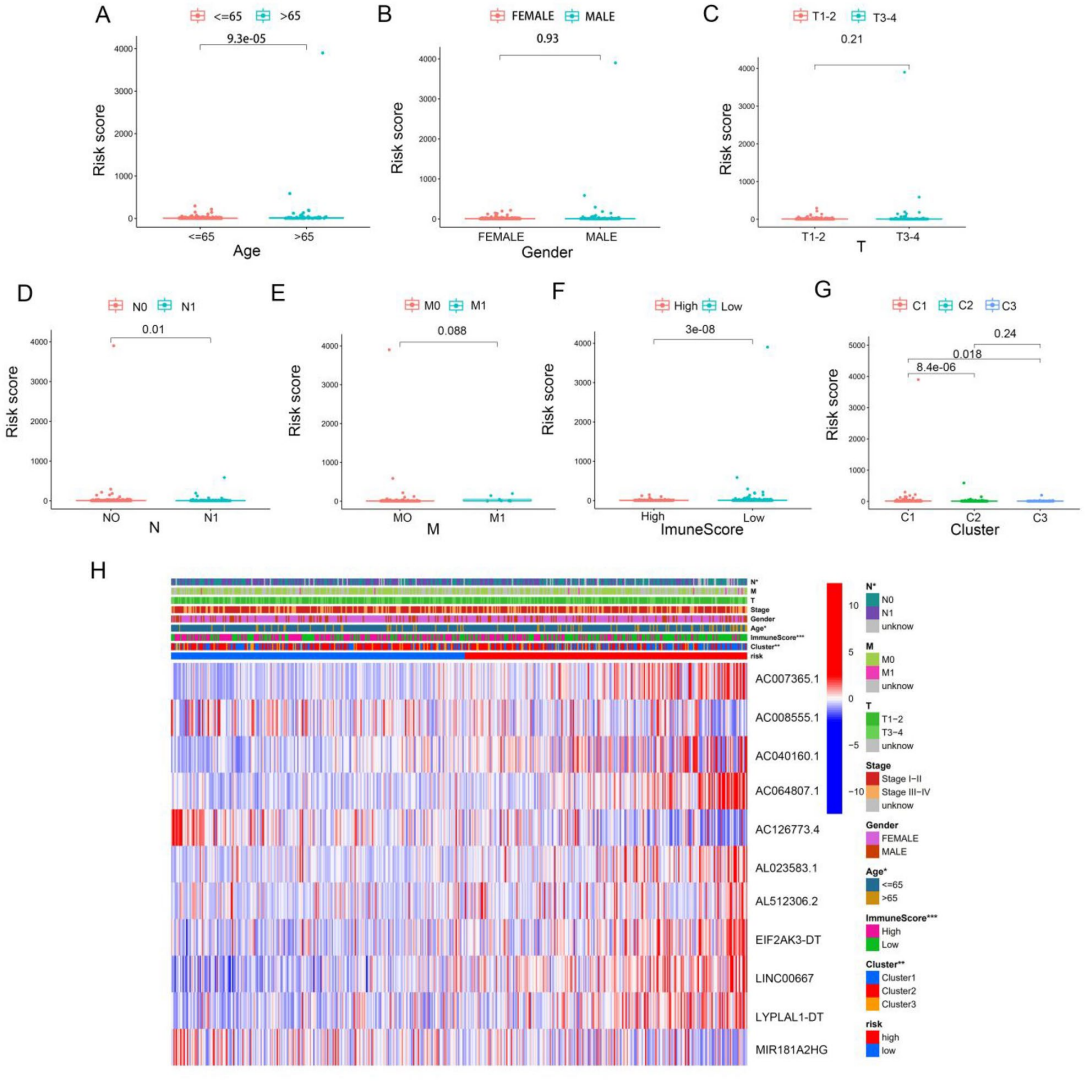
Risk model score associated with clinicopathological features, including age (A); grade (B); T stage (C); M stage (D); N stage (E); clusters 1, 2 and 3 (F); and immunoscore (G). Heatmap demonstrating the clinicopathologic features between two risk groups (H).

In order to further investigate the correlation between risk scores and clinical characteristics, we analysed the differences in risk scores between subgroups stratified by clinical characteristics. Our findings revealed that the risk score was highest in THCA patients older than 65 years (Figure 7(A)). No major differences in risk scores were observed in relation to gender or T and M stage (Figure 7(B)–(E)). In terms of immune score, the risk score in the higher immune score group was lower than that in the lower immunoscore group (Figure 7(F)). Moreover, the risk model score was higher in cluster 1 than that in the other clusters (Figure 7(G)). Compared to the other consensus clusters, cluster 1 had a significantly higher risk score and poorer prognosis than clusters 2 and 3.

Next, we performed subgroup analyses, and found that worse outcomes in THCA patients in the high-risk group were correlated with age (Supplementary Figure S3(A)–(B)), gender (Supplementary Figure S3(C) and (D)), M0 stage (Supplementary Figure S3 (E)), N stage (Supplementary Figure S3 (G) and (H)), T3–T4 stage (Supplementary Figure S3(J)) and pathological stages III–IV (Supplementary Figure S3(L)). The *p* values associated with the above subgroups, except for the M1 stage (Supplementary Figure S3(F)) and pathological stage I–II subgroups (Supplementary Figure S3(K)), were <0.05. Compared to the low-risk group, m6A-associated lncRNA, including AL023583.1, AC007365.1, AC040160.1, AC064807.1, AL512306.2, EIF2AK3-DT, LINC00667 and LYPLAL1-DT levels were higher in the high-risk group, whereas AC00855.1, AC126773.4 and MIR181A2HG were decreased (Figure 7(H) and Supplementary Figure S4(A)). The upper bar of the heatmap also identified significant differences in age, N stage, immunoscore and cluster groups between the two risk groups.

### 3.7. Knockdown of LncRNA MIR181A2HG or LYPLAL1-DT overexpression affected the proliferation and migration of PTC cells in vitro

**Figure 8.**
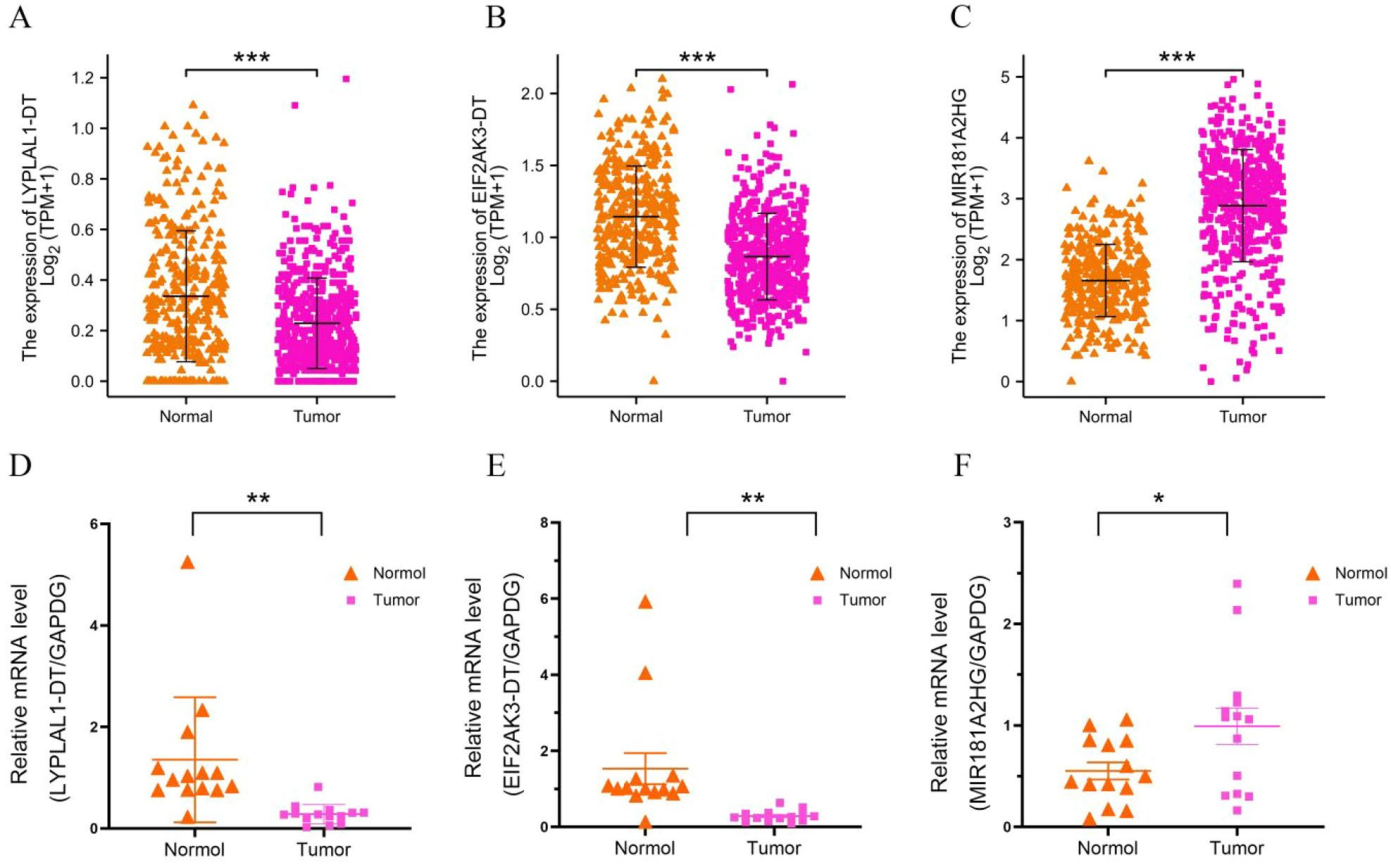
Differential expression analysis of THCA patients with LYPLAL1-DT, EIF2AK3-DT and MIR181A2HG. LYPLAL1-DT(A), EIF2AK3-DT(B) and MIR181A2HG(C) expression based on TCGA-THCA dataset. qPCR analysis of LYPLAL1-DT(D), EIF2AK3-DT(E) and MIR181A2HG(F) expression in 14 pairs of THCA and adjacent normal tissues. An unpaired Student’s *t* test was used to determine the significance of differences. *p < 0.05; **p < 0.01; ***p < 0.001.

In our study, 11 m6A-related lncRNAs were used to construct a prognosis model. For verification purposes, we initially selected four lncRNAs in the model with relatively low scores: MIR181A2HG, LYPLAL1-DT, EIF2AK3-DT and LINC00667. Our expectation was that if these four lncRNAs can be successfully verified, lncRNAs with higher scores should have even better chances of being correct. Analysis of TCGA data showed that MIR181A2HG was highly expressed in thyroid cancer, and LYPLAL1-DT, EIF2AK3-DT and LINC00667 were suppressed in cancer tissue (Figure 8(A–C)). We then validated the expression of these genes in our thyroid cancer tissue samples (Figure 8(D–F)). However, construction of the linc00667 expression plasmid failed, as the linc00667 fragment was too large (5489 bp). No further experiments were carried out on linc00667, thus related linc00667 qPCR data were not included in this manuscript. Since the functional experiments required to study LYPLAL1-DT and EIF2AK3-DT are the same, LYPLAL1-DT was chosen for subsequent functional testing. Additionally, we constructed plasmids to knockdown a high expression gene (MIR181A2HG) and overexpress a low expression gene (LYPLAL1-DT) for subsequent functional tests.

**Figure 9.**
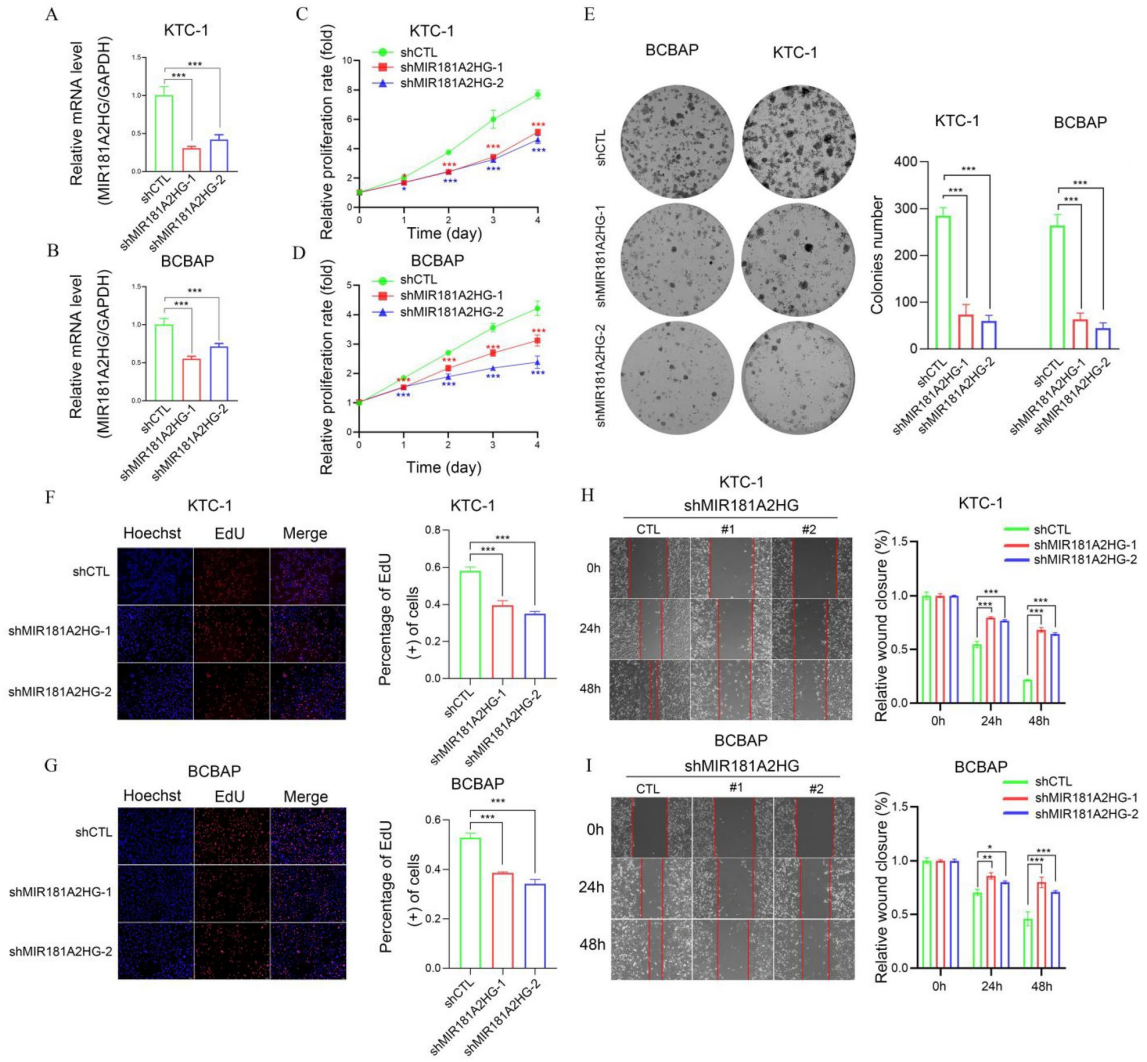
Knockdown of MIR181A2HG inhibited the proliferation and migration of PTC cells *in vitro*. (A–B) Relative MIR181A2HG expression in KTC1 and BCBAP cells transfected with two independent shRNAs targeting MIR181A2HG by qPCR. (C–D) KTC1 and BCBAP cell proliferation after knockdown of MIR181A2HG by MTT assay. (E–G) Representative results of the colony formation (scale bar:100 μm), and EdU assays (scale bar:100 μm) in KTC1 and BCBAP cells after MIR181A2HG-sh1 or MIR181A2HG-sh2 transfection. (H–I) MIR181A2HG knockdown suppressed migration capabilities in KTC1 and BCBAP cells. *p < 0.05; **p < 0.01; ***p < 0.001.

**Figure 10.**
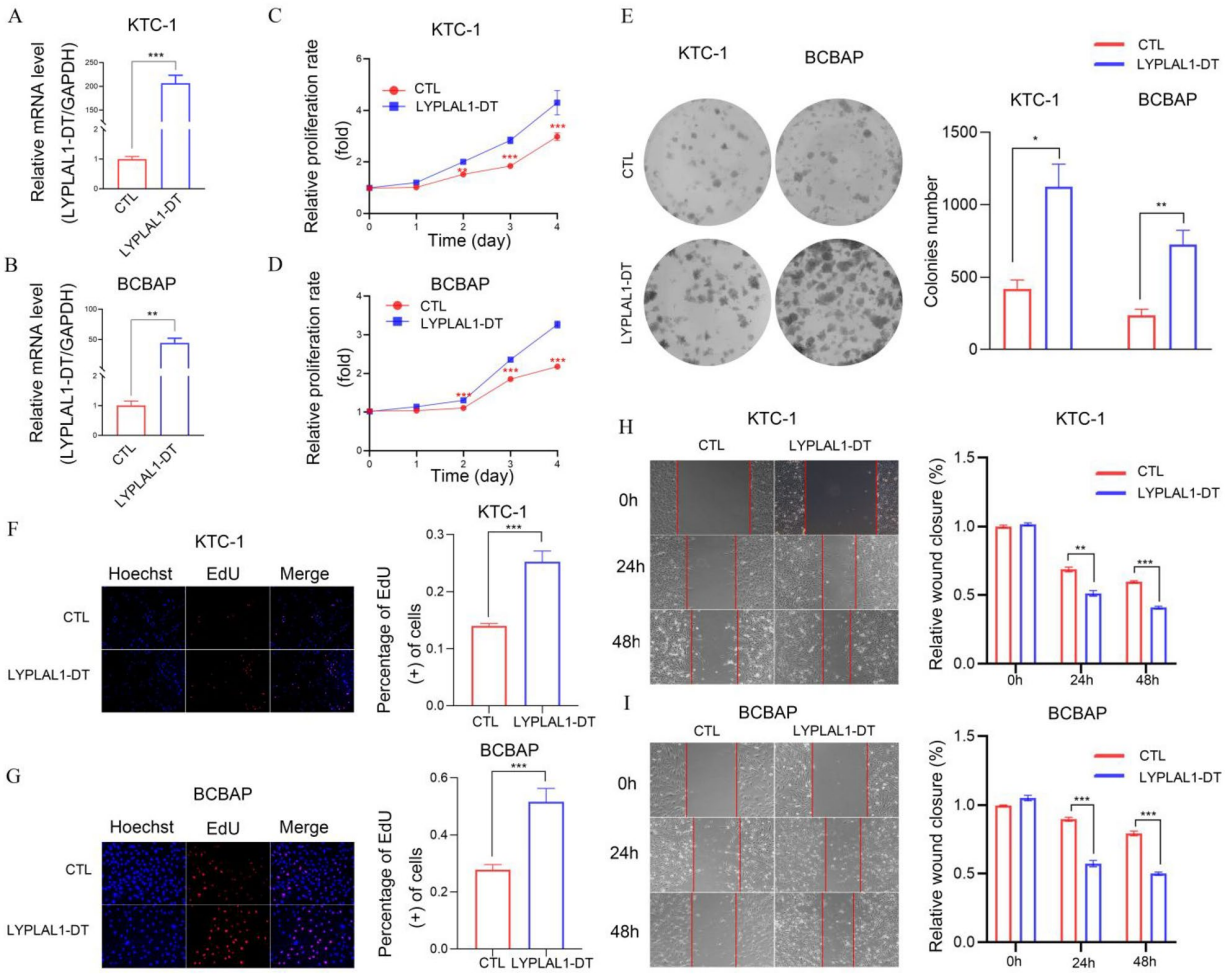
Overexpression of LYPLAL1-DT promoted the proliferation and migration of PTC and BCBAP cells *in vitro*. (A–B) Relative LYPLAL1-DT expression in KTC1 and BCBAP cells by qPCR. (C–D) KTC1 and BCBAP cell proliferation after overexpression of LYPLAL1-DT by MTT assay. (E–G) Representative results of the colony formation (scale bar:100 μm), and EdU assays (scale bar:100 μm) in KTC1 and BCBAP cells after overexpression of LYPLAL1-DT. (H–I) Overexpression of LYPLAL1-DT promoted migration capabilities in KTC1 and BCBAP cells. *p < 0.05; **p < 0.01; ***p < 0.001.

We further investigated the involvement of MIR181A2HG and LYPLAL1-DT in PTC *in vitro*. First, shCTL/shMIR181A2HG cell lines of KTC-1 and BCPAP were constructed by lentivirus transfection. qPCR results showed that the expression of MIR181A2HG was significantly reduced in the two cell lines (Figure 9(A,B)). Then, MTT (Figure 9(C,D)), EdU (Figure 9(F,G)) and colony formation assays (Figure 9(E)) were conducted to determine the role of MIR181A2HG on PTC cell proliferation. Knockdown of MIR181A2HG significantly inhibited proliferation of KTC-1 and BCBAP cells (*p* < 0.01). Subsequently, the wound-healing assay was used to detect the migration of PTC cells. The migration of MIR181A2HG-knockdown cells was significantly inhibited compared with the control group (Figure 9(H,I)). Similarly, to further confirm roles of LYPLAL1-DT in PTC, LYPLAL1-DT was overexpressed in KTC1 and BCBAP cells (Figure 10(A,B)). As expected, LYPLAL1-DT overexpression promoted cell proliferation and migration in PTC, indicated by MTT assays (Figure 10(C,D)), colony formation (Figure 10(E)), EdU (Figure 10(F,G)) and wound-healing assays (Figure 10(H,I)). These results collectively showed that lncRNA MIR181A2HG and LYPLAL1-DT affected the proliferation and migration of PTC cells.

### 3.8. Correlation analysis between the risk model score and TME, including immune checkpoint and immune cell infiltration

**Figure 11.**
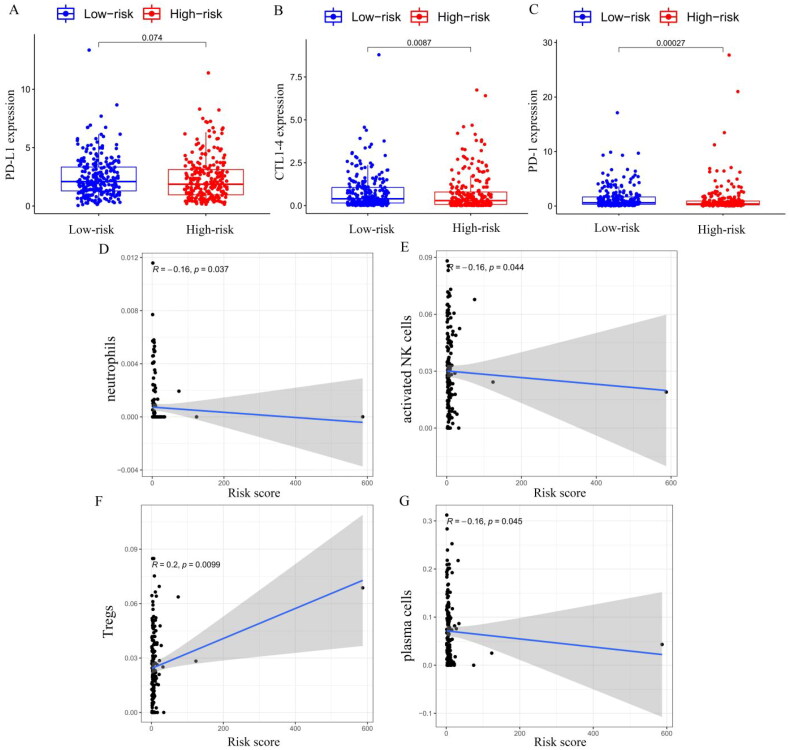
The PD-L1 (A), CTLA-4 (B) and PD-1 (C) expression levels by the risk score groups. Correlation between immune cells infiltration and risk score for (D) neutrophils, (E) activated natural killer cells, (F) regulatory T cells (Tregs) and (G) plasma cells.

Blocking immune checkpoints has become a popular approach to cancer treatment. To identify the association between immune checkpoint and the risk score, we compared the expression levels of immune checkpoint genes PD-L1, CTLA-4 and PD-1 in the high- and low-risk groups (Figure 11(A–C)), and we found that the expressions of CTLA-4 (*p* = 0.0087) and PD-1 (*p* = 0.00027) were higher in the low-risk group than in the high-risk group. We used the CIBERSORT algorithm to analyse immune cell infiltration in the high- and low-risk groups. Correlation analysis showed that neutrophils (Figure 11(D)), activated NK cells (Figure 11(E)) and plasma cells (Figure 11(G)) were negatively correlated with the risk model score, and regulatory T cells (Tregs) were positively correlated with the risk model score (*p* < 0.05, Figure 11(F)). However, no significant correlations were observed in relation to other types of immune cells. In addition to CIBERSORT, we used a different method of immune deconvolution analysis, ssGSEA [34], to further characterize the differences in immunological function. Using the ssGSEA method, we compared the infiltration of 23 tumour immune cells between high-risk and low-risk THCA patients. As shown in Supplementary Figure S4(B), the risk score was negatively correlated with most tumour immune cell types. Furthermore, the levels of most tumour immune cell types differed between the high-risk and low-risk groups. The low-risk group had a higher level of immune cell infiltrations than that of the high-risk group, and the correlation analysis demonstrated that the risk score was negatively correlated with the levels of most immune cells, except for CD56 dim natural killer cells, eosinophilia, monocyte and type 17 T helper cells (Supplementary Figure S4(C)). Collectively, our risk score may allow the assessment of the tumour immune microenvironment to further ascertain whether immunotherapy can be generally applied in THCA patients.

## 4. Discussion

Several studies have demonstrated that m6A modification may play a regulatory role in cancer pathogenesis; however, its role in THCA progression by regulating lncRNA is poorly understood. In our study, we identified 70 m6A-associated prognostic lncRNAs from 502 TCGA–THCA patients, subsequently, the TCGA–THCA dataset was divided into three cluster groups by consensus expression of 70 m6A-associated prognostic lncRNAs. Notably, a significant survival difference was found among the three groups, cluster 1 had worse survival and higher expression of m6A-associated lncRNAs than the other clusters. Moreover, we also found that lower immune and ESTIMATE scores were associated with poorer prognosis in cluster 1 compared with the other clusters. Regarding immune checkpoints, cluster 1, which showed worse OS, had a low level of PD-L1 expression, which indicated those patients would not respond to immune checkpoint inhibitors. These results indicate that m6A-associated lncRNAs exert vital functions in the tumour immune microenvironment and correlate with the prognosis of THCA patients. Subsequently, based on LASSO regression analysis, 11 of the m6A-associated lncRNAs were screened from the 70 m6A-associated prognostic lncRNAs to establish the risk model to accurately evaluate risk stratification in individual samples. OS analysis demonstrated that there were dramatic differences between the low- and high-risk groups. Subgroup analysis demonstrated that risk score is effective for further distinguishing different subgroups, and significant differences were observed with respect to age (*p* < 0.05), N stage (*p* < 0.05), immunoscore (*p* < 0.001) and cluster groups (*p* < 0.01) between the two risk groups. All the above results confirm that risk stratification according to the m6A-associated lncRNAs risk model would improve personalized therapy and improve outcomes of THCA patients.

Using the ssGSEA method, we found that the levels of most tumour immune cell types differed between the high-risk and low-risk groups. The low-risk group exhibited a higher level of immune cell infiltration than the high-risk group, and the correlation analysis demonstrated that the risk score was negatively correlated with the levels of most immune cells. Similarly, the high-risk group was characterized by lower expression of immune checkpoint genes (CTLA-4 and PD-1), which was negatively correlated with poorer outcomes. Together, these results suggest that the immune microenvironment plays an important role in the occurrence and progression of THCA and is closely related to the prognosis of THCA patients. Interestingly, we found the highest levels of CD8T cell infiltration in cluster 1, which also had the worst prognosis. Previous studies have demonstrated that CD8+ T cells can exert antitumour effects against multiple cancer types, and high levels of CD8+ T cell infiltration are associated with better prognosis. Recent research has discovered that CD8+ T cell status is correlated with poorer OS in invasive mucinous adenocarcinoma [35] and renal cell carcinoma (RCC) [36–38]. Nakano et al. indicated that the negative impact of CD8+ T cells and CD4+ T cells on prognosis was mainly attributed to the cancer grade or the proliferative activity of RCC cells, and infiltrations of CD8+ T cells were not correlated with the efficacy of antitumour immunity [38]. Petitprez et al. [39] also demonstrated that a higher progression rate was observed in prostate cancer patients with high CD8+ T cell counts. Moreover, Leclerc et al. showed that CD73 suppresses immune surveillance mediated by CD8+ T cells and converts them into cancer-promoting factors [40]. Likewise, Ness et al. [41] also found that CD8+ T cells have immunosuppressive capabilities, which is an important mechanism that underlies cancer development. While the specific mechanisms remain poorly understood, our results suggest that THCA patients with higher levels of CD8+ T cells tend to have worse clinical outcomes.

In order to further verify the reliability of our risk model, we detected the RNA expression levels of the representative genes in the model in thyroid cancer tissue samples. Consistent with TCGA database analysis, MIR181A2HG was highly expressed in thyroid cancer, while LYPLAL1-DT, EIF2AK3-DT and LINC00667 were low expressed in cancer tissues. MIR181A2HG has been reported to be associated with the prognosis of bladder cancer [42] and THCA [43,44]. Furthermore, MIR181A2HG may also impair the proliferation and migration of vascular endothelial cells through the miRNAs/AKT2 axis [45]. Similarly, Zhu et al. showed that LncRNA LYPLAL1-DT could regulate the process of autophagy through the miR-204-5p/SIRT1 axis [46]. In Zhu’s study, the overexpression of LYPLAL1-DT facilitated endothelial cell proliferation and migration, increased autophagy activity. Additionally, monocytic cells adherence to endothelial cells was also reduced by LYPLAL1-DT [46]. Interestingly, functional experiments in our study showed that knockdown of MIR181A2HG obviously inhibited the proliferation and migration of PTC cells *in vitro*, which was consistent with the finding of previous studies. Whereas LYPLAL1-DT overexpression promoted PTC cell proliferation and migration. The above results verified the reliability of our risk score in tissue and *in vitro* cell models, respectively.

At present, reports on how the m6A-associated regulator lncRNAs interact with the tumour immune response have been limited. Wang et al. [47] constructed a risk model based on eight m6A-associated lncRNAs. Consistent with our results, this study highlighted that m6A-associated lncRNAs are closely related to immune regulation in thyroid cancer. However, unlike the study by Wang et al. we also verified the reliability of our model through functional experiments on several factors from the risk model. Moreover, we verified the reliability of our risk score in both clinical sample tissues and *in vitro* cell models. In our study, the risk scores were evidently associated with immune cells and immune checkpoints. Furthermore, the scores were inversely related to the level of neutrophils, activated NK and plasma cells. Recent studies have demonstrated that neutrophils play a vital role in the antitumour effect [48,49]. Similarly, NK cells are well-known antitumour factors, as reported in several studies [50–52]. The low levels of NK cells and neutrophils, and low expression of immune checkpoint genes PD-L1 and CTLA-4 seem to be markedly correlated with worse prognosis in our risk model.

## 5. Conclusions

In the present study, we sought to examine how m6A-associated lncRNAs interact with the TME and affect the prognosis of patients with THCA. Our study identified 70 m6A-associated prognostic lncRNAs. Among them 11 lincRNAs were used to construct an m6A-associated lncRNA risk model, which might be used to predict the clinical outcomes of THCA patients. Two selected lncRNAs MIR181A2HG and LYPLAL1-DT were further verified in clinical samples and THCA cell lines. The present study may provide a novel and efficient immunotherapeutic strategy for the treatment of THCA patients.

## Supplementary Material

Supplemental Material

## Data Availability

Data are contained within the article or Supplementary. Further inquiries can be directed to the corresponding author.
