# Supplementary material for: Identification of m6A-associated LncRNAs as predict factors for the immune infiltration and prognosis of thyroid cancer
Source: Ann Med. 2023 Mar 28;55(1):1298–316. doi: 10.1080/07853890.2023.2192049 (PMC10054316; doi:10.1080/07853890.2023.2192049)
Supplement: Supplemental Material [file IANN_A_2192049_SM9212.docx]

Supporting Information


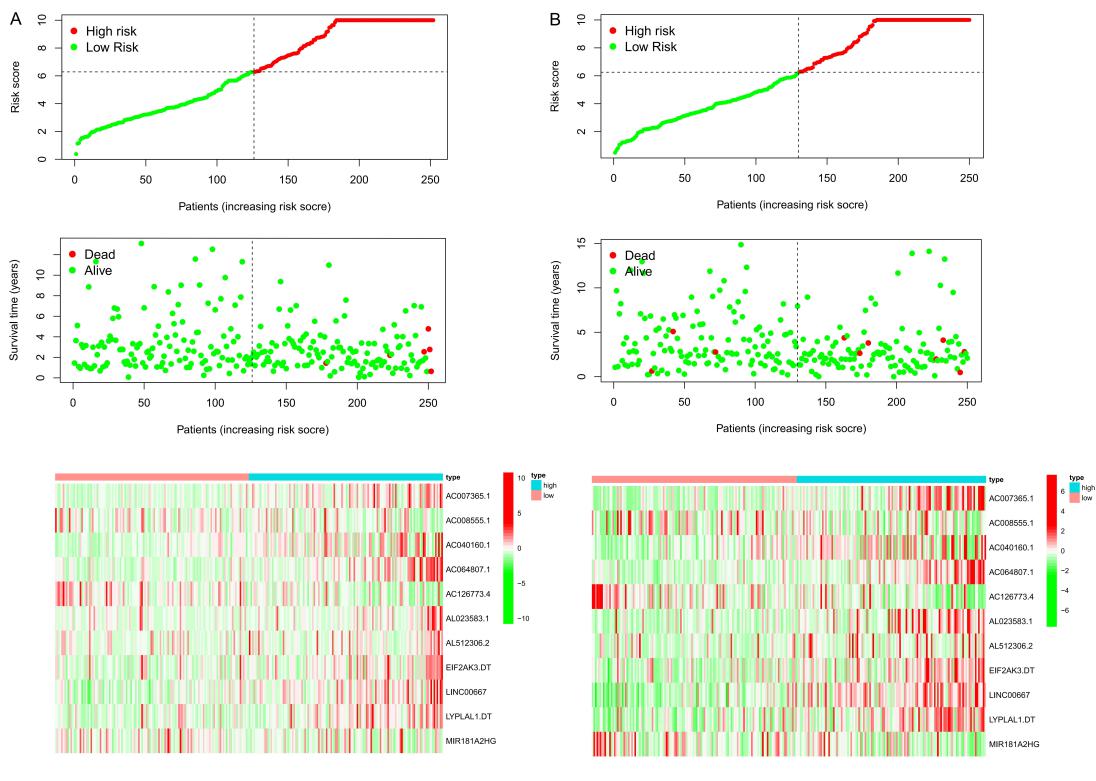


**Figure S1.** Risk model can distinguish high-risk and low-risk patients efficiently. The risk scores plot, overall survival status, and heatmap of 11 N^6^-methyladenosine-associated prognostic long noncoding RNAs in the training (A) and testing cohorts (B).


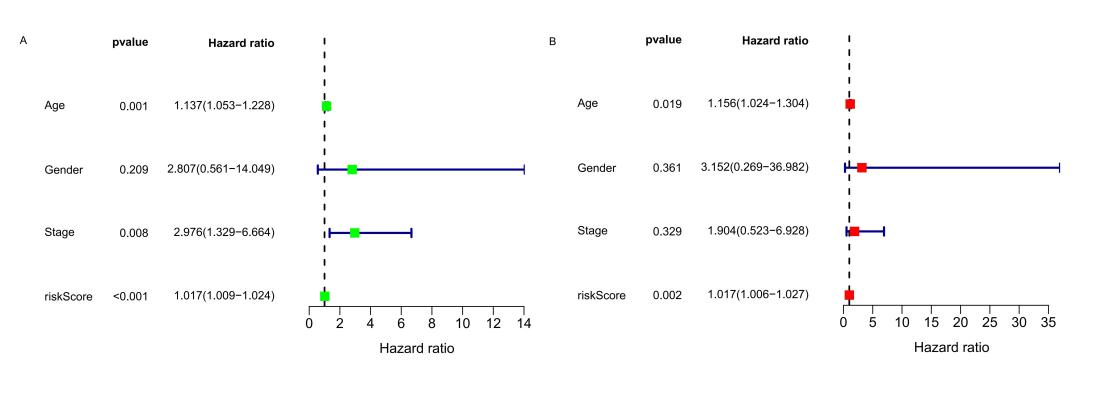


**Figure S2.** Risk model score as independent prognostic indicators. Validation of the independence of the m^6^A-associated prognostic lncRNAs in the overall survival through the univariate (A) and multivariate Cox regression analyses (B).


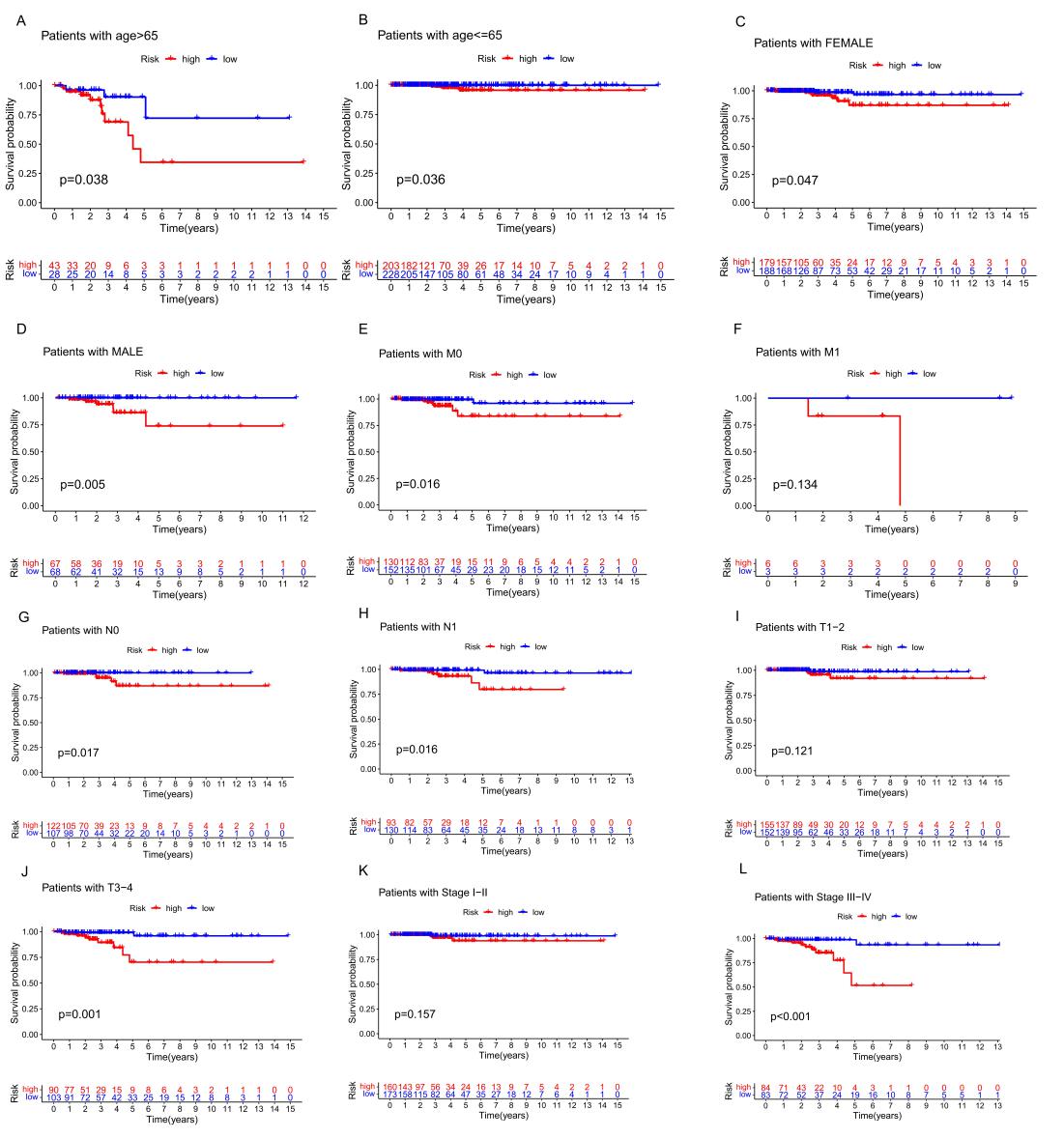


**Figure S3.** Survival analysis of the risk score for THCA patients within different clinicopathological characteristics. Subgroup overall survival analysis, including age (A, B), sex (C, D), TNM stage (E-J), and pathological stage (K, L).


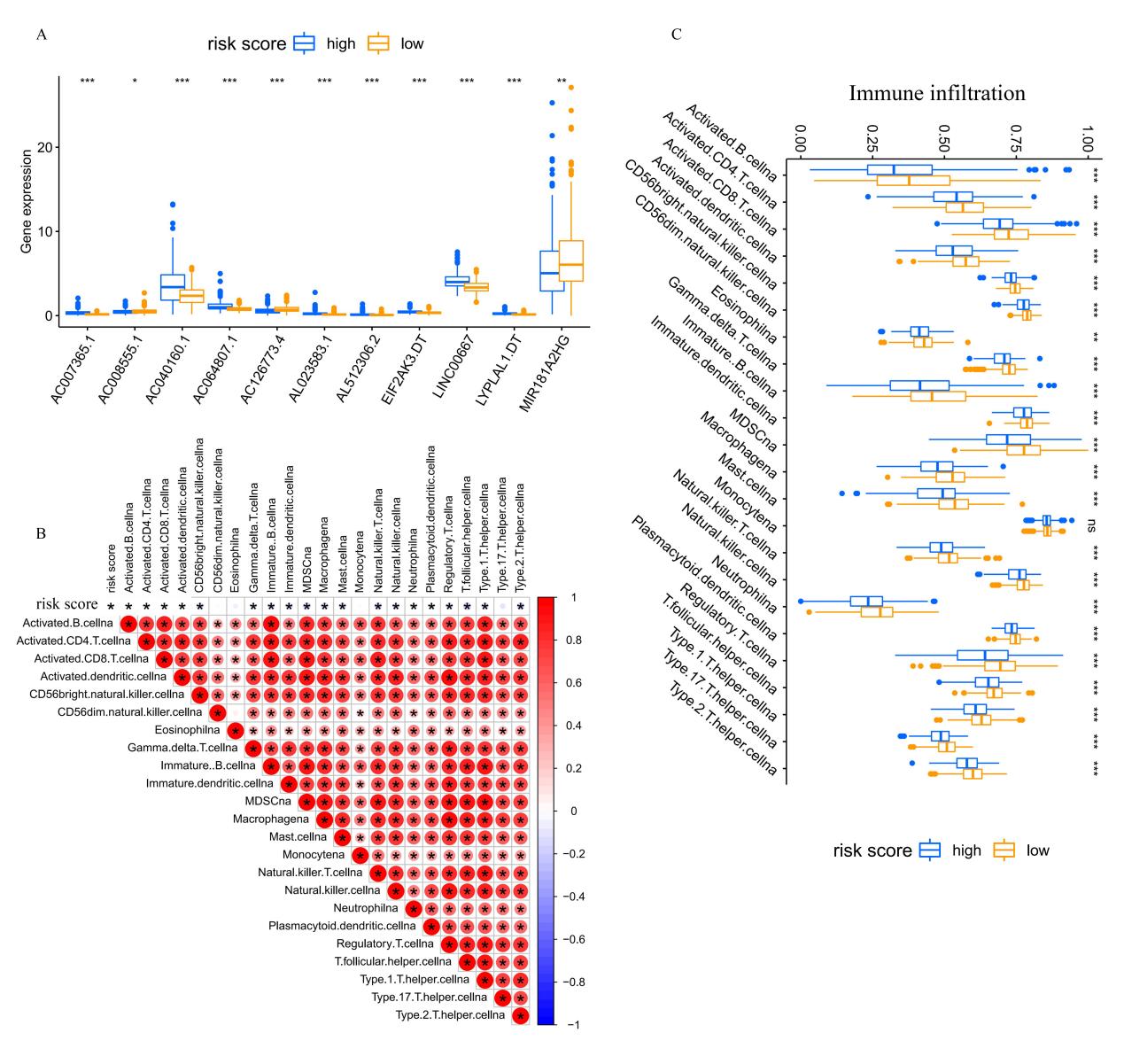


Figure S4. Correlation analysis between the risk model score and immune cell infiltration. (A) Differences expression of m6A-associated lncRNAs between low- and high-risk score groups. (B) Correlation analysis between risk score and and TME infiltration cells. (*p < 0.05; Blue, negative correlations; Red, positive correlations). (C) The abundance of each TME infiltrating cell in the high-risk and low-risk groups. (ns: not significant, *p < 0.05, ** p < 0.01, *** p < 0.001).

**Table S1.** Targeting sequence information of vectors used in the study.

| **LncRNA** | **Vector type** | **Targeting sequence** |
| --- | --- | --- |
| MIR181A2HG | shRNA1 plasmid | GCAAGAATCCTATCTGGAAA |
|  | shRNA2 plasmid | GGCGAACTGTCCTGTGGAAAG |

**Table S2.** Primer sequences list

| **LncRNA** | **Sequence** |
| --- | --- |
| LYPLAL1-DT | Forward: GGCTCCCAGATGACATTTTAGGAC  Reverse: ATGTTCACTCAAGGCCCAAGCA |
| EIF2AK3-DT | Forward: TGTGACCAACCAGCTACGTGA  Reverse: TGTGACCAACCAGCTACGTGA |
| MIR181A2HG | Forward: TCTAGCCACAGCAGCTCGAA  Reverse: CTGCATCCAGTAAAAGAACCGAAG |
| GAPDH | Forward: TGCACCACCAACTGCTTAGC  Reverse: GGCATGGACTGTGGTCATGAG |

**Table S3. The three hundred and twenty-two m6A-related prognostic lncRNAs in THCA patients.**

| **m6A** | **lncRNA** | **cor** | **P-value** |
| --- | --- | --- | --- |
| YTHDC1 | A2M-AS1 | 0.430401 | 2.05E-24 |
| YTHDF1 | A2M-AS1 | 0.429107 | 2.91E-24 |
| FTO | A2M-AS1 | 0.467649 | 4.49E-29 |
| ALKBH5 | A2M-AS1 | 0.499574 | 1.50E-33 |
| ALKBH5 | AC002401.4 | -0.41495 | 1.21E-22 |
| ALKBH5 | AC002451.1 | 0.442068 | 8.20E-26 |
| ALKBH5 | AC002467.1 | 0.534614 | 4.86E-39 |
| IGFBP3 | AC004540.1 | 0.626052 | 7.46E-57 |
| ALKBH5 | AC004825.2 | 0.45746 | 9.65E-28 |
| METTL3 | AC004923.4 | 0.440115 | 1.42E-25 |
| METTL14 | AC004943.2 | 0.496629 | 4.06E-33 |
| VIRMA | AC004943.2 | 0.420105 | 3.17E-23 |
| YTHDC1 | AC004943.2 | 0.575433 | 2.66E-46 |
| FTO | AC004943.2 | 0.513312 | 1.26E-35 |
| ALKBH5 | AC004943.2 | 0.419284 | 3.93E-23 |
| METTL3 | AC005014.2 | 0.403785 | 2.02E-21 |
| ALKBH5 | AC005034.5 | 0.488696 | 5.68E-32 |
| ALKBH5 | AC005076.2 | 0.475895 | 3.47E-30 |
| HNRNPC | AC005225.2 | -0.40733 | 8.36E-22 |
| ALKBH5 | AC005225.2 | 0.421889 | 1.99E-23 |
| METTL3 | AC005387.2 | 0.55615 | 9.57E-43 |
| METTL14 | AC006213.1 | 0.469392 | 2.63E-29 |
| YTHDC1 | AC006213.1 | 0.486622 | 1.12E-31 |
| FTO | AC006213.1 | 0.420568 | 2.81E-23 |
| ALKBH5 | AC006213.1 | 0.433331 | 9.25E-25 |
| FTO | AC006333.1 | 0.445299 | 3.29E-26 |
| ALKBH5 | AC006333.1 | 0.509067 | 5.64E-35 |
| METTL3 | AC007066.2 | 0.58449 | 4.70E-48 |
| YTHDC1 | AC007066.2 | 0.45645 | 1.30E-27 |
| YTHDF1 | AC007066.2 | 0.41764 | 6.03E-23 |
| HNRNPA2B1 | AC007066.2 | 0.454351 | 2.41E-27 |
| METTL3 | AC007336.1 | 0.508096 | 7.93E-35 |
| YTHDC2 | AC007336.1 | 0.439379 | 1.74E-25 |
| FTO | AC007365.1 | 0.48321 | 3.38E-31 |
| ALKBH5 | AC007365.1 | 0.506144 | 1.57E-34 |
| LRPPRC | AC007405.4 | 0.483209 | 3.38E-31 |
| ALKBH5 | AC008014.1 | 0.560195 | 1.80E-43 |
| ALKBH5 | AC008268.1 | 0.499142 | 1.74E-33 |
| ZC3H13 | AC008555.1 | 0.414482 | 1.36E-22 |
| YTHDC1 | AC008555.1 | 0.454199 | 2.52E-27 |
| YTHDF3 | AC008555.1 | 0.438012 | 2.55E-25 |
| RBMX | AC008555.1 | 0.421736 | 2.07E-23 |
| METTL14 | AC008669.1 | 0.429074 | 2.94E-24 |
| FMR1 | AC008669.1 | 0.456025 | 1.47E-27 |
| RBMX | AC008669.1 | 0.45063 | 7.12E-27 |
| VIRMA | AC008736.1 | -0.43632 | 4.07E-25 |
| ZC3H13 | AC008736.1 | -0.45523 | 1.86E-27 |
| YTHDF3 | AC008736.1 | -0.41578 | 9.75E-23 |
| YTHDF1 | AC008738.2 | 0.413019 | 1.98E-22 |
| METTL3 | AC008760.1 | 0.557072 | 6.55E-43 |
| RBMX | AC008760.1 | 0.457287 | 1.02E-27 |
| METTL3 | AC008870.3 | 0.614071 | 3.50E-54 |
| LRPPRC | AC008870.3 | -0.40588 | 1.20E-21 |
| FTO | AC009041.3 | 0.471064 | 1.57E-29 |
| METTL3 | AC009090.3 | 0.403298 | 2.28E-21 |
| ALKBH5 | AC009779.3 | 0.60217 | 1.22E-51 |
| RBM15B | AC009831.1 | 0.440856 | 1.15E-25 |
| RBMX | AC009831.1 | 0.428046 | 3.87E-24 |
| ALKBH5 | AC010226.1 | 0.413735 | 1.65E-22 |
| YTHDF1 | AC010536.3 | 0.429259 | 2.79E-24 |
| ALKBH5 | AC010735.2 | 0.504407 | 2.86E-34 |
| METTL3 | AC010809.2 | 0.453411 | 3.17E-27 |
| ALKBH5 | AC010857.1 | 0.460773 | 3.60E-28 |
| METTL3 | AC010864.1 | 0.466293 | 6.79E-29 |
| ALKBH5 | AC010980.2 | 0.484747 | 2.06E-31 |
| ALKBH5 | AC011700.1 | 0.457178 | 1.05E-27 |
| METTL3 | AC011921.1 | 0.554884 | 1.61E-42 |
| ALKBH5 | AC011921.1 | 0.472328 | 1.06E-29 |
| METTL3 | AC012181.1 | 0.442727 | 6.81E-26 |
| YTHDC2 | AC012181.1 | 0.424568 | 9.80E-24 |
| HNRNPA2B1 | AC012447.1 | 0.454331 | 2.43E-27 |
| ALKBH5 | AC012462.3 | -0.40469 | 1.61E-21 |
| FTO | AC012464.1 | 0.463975 | 1.37E-28 |
| HNRNPC | AC012668.3 | 0.445914 | 2.76E-26 |
| FMR1 | AC012668.3 | 0.419711 | 3.52E-23 |
| ALKBH5 | AC012668.3 | -0.43432 | 7.05E-25 |
| YTHDF1 | AC015726.1 | 0.415448 | 1.06E-22 |
| LRPPRC | AC015726.1 | -0.41122 | 3.14E-22 |
| METTL3 | AC016737.1 | 0.547295 | 3.45E-41 |
| ALKBH5 | AC018521.5 | 0.453581 | 3.02E-27 |
| FTO | AC018816.1 | -0.47154 | 1.35E-29 |
| METTL3 | AC019080.1 | 0.473425 | 7.53E-30 |
| YTHDC1 | AC019080.1 | 0.413918 | 1.57E-22 |
| YTHDC2 | AC019080.1 | 0.492185 | 1.80E-32 |
| IGFBP3 | AC022893.1 | 0.680237 | 1.53E-70 |
| LRPPRC | AC023302.1 | -0.41347 | 1.76E-22 |
| METTL3 | AC023510.2 | 0.470908 | 1.65E-29 |
| YTHDF1 | AC023510.2 | 0.41784 | 5.73E-23 |
| METTL3 | AC023632.2 | 0.414062 | 1.52E-22 |
| METTL3 | AC025682.2 | 0.447362 | 1.83E-26 |
| ALKBH5 | AC025682.2 | 0.453426 | 3.16E-27 |
| ALKBH5 | AC025871.2 | 0.413278 | 1.85E-22 |
| ALKBH5 | AC026116.1 | 0.432887 | 1.04E-24 |
| HNRNPC | AC026356.1 | 0.407044 | 8.97E-22 |
| RBMX | AC026356.2 | 0.40545 | 1.34E-21 |
| ALKBH5 | AC026704.1 | 0.443407 | 5.62E-26 |
| ALKBH5 | AC027281.1 | 0.450875 | 6.64E-27 |
| LRPPRC | AC027373.1 | -0.41026 | 4.00E-22 |
| METTL3 | AC027763.2 | 0.40257 | 2.73E-21 |
| METTL3 | AC027796.4 | 0.483869 | 2.73E-31 |
| YTHDF1 | AC034229.4 | 0.464526 | 1.16E-28 |
| ALKBH5 | AC035140.1 | 0.413865 | 1.60E-22 |
| FMR1 | AC040160.1 | 0.420291 | 3.02E-23 |
| RBMX | AC040160.1 | 0.531069 | 1.87E-38 |
| HNRNPC | AC046143.1 | 0.407514 | 7.97E-22 |
| ALKBH5 | AC063943.2 | 0.518785 | 1.76E-36 |
| ALKBH5 | AC064807.1 | 0.559239 | 2.67E-43 |
| ALKBH5 | AC064807.4 | 0.431746 | 1.42E-24 |
| METTL3 | AC067750.1 | 0.433831 | 8.07E-25 |
| ALKBH5 | AC067750.1 | 0.467128 | 5.27E-29 |
| METTL14 | AC068700.1 | 0.415449 | 1.06E-22 |
| VIRMA | AC069281.2 | -0.46979 | 2.33E-29 |
| ZC3H13 | AC069281.2 | -0.44238 | 7.52E-26 |
| LRPPRC | AC069281.2 | -0.47514 | 4.41E-30 |
| FTO | AC069281.2 | -0.42577 | 7.11E-24 |
| METTL3 | AC073487.1 | 0.563075 | 5.38E-44 |
| YTHDC2 | AC073487.1 | 0.463041 | 1.82E-28 |
| METTL3 | AC079075.1 | 0.505998 | 1.65E-34 |
| YTHDC1 | AC079075.1 | 0.44203 | 8.29E-26 |
| YTHDC2 | AC079075.1 | 0.404239 | 1.80E-21 |
| YTHDF1 | AC079414.3 | 0.422459 | 1.71E-23 |
| METTL3 | AC079684.2 | 0.446595 | 2.27E-26 |
| METTL3 | AC079807.1 | 0.607166 | 1.08E-52 |
| YTHDC1 | AC079807.1 | 0.486398 | 1.20E-31 |
| YTHDC2 | AC079807.1 | 0.430705 | 1.89E-24 |
| HNRNPA2B1 | AC079807.1 | 0.459215 | 5.73E-28 |
| FTO | AC079848.1 | 0.503593 | 3.79E-34 |
| ALKBH5 | AC079848.1 | 0.570742 | 2.05E-45 |
| RBM15B | AC084809.1 | 0.435334 | 5.34E-25 |
| RBMX | AC084809.1 | 0.45428 | 2.46E-27 |
| METTL14 | AC087854.1 | 0.411075 | 3.25E-22 |
| YTHDC1 | AC087854.1 | 0.406326 | 1.07E-21 |
| YTHDC2 | AC087854.1 | 0.46528 | 9.25E-29 |
| HNRNPA2B1 | AC087854.1 | 0.414089 | 1.51E-22 |
| METTL16 | AC090114.2 | 0.400397 | 4.65E-21 |
| METTL3 | AC090246.1 | 0.405304 | 1.38E-21 |
| YTHDF1 | AC090246.1 | 0.401015 | 3.99E-21 |
| ALKBH5 | AC090246.1 | 0.483864 | 2.74E-31 |
| YTHDC2 | AC090739.1 | 0.42474 | 9.36E-24 |
| METTL3 | AC090948.3 | 0.471651 | 1.31E-29 |
| IGFBP2 | AC091138.1 | 0.414042 | 1.52E-22 |
| ALKBH5 | AC091965.1 | 0.407012 | 9.04E-22 |
| ZC3H13 | AC092171.5 | -0.43423 | 7.23E-25 |
| ALKBH5 | AC092718.1 | 0.453599 | 3.00E-27 |
| METTL3 | AC092794.1 | 0.587874 | 1.01E-48 |
| METTL3 | AC095057.3 | 0.456229 | 1.39E-27 |
| YTHDF1 | AC095057.3 | 0.462642 | 2.05E-28 |
| YTHDC1 | AC096536.1 | 0.447922 | 1.55E-26 |
| YTHDC2 | AC096536.1 | 0.436515 | 3.86E-25 |
| ALKBH5 | AC096734.2 | 0.450854 | 6.68E-27 |
| ALKBH5 | AC096920.1 | 0.435335 | 5.34E-25 |
| METTL3 | AC096992.2 | 0.491927 | 1.96E-32 |
| YTHDC1 | AC096992.2 | 0.404528 | 1.68E-21 |
| HNRNPA2B1 | AC096992.2 | 0.432851 | 1.05E-24 |
| ALKBH5 | AC096992.2 | 0.423375 | 1.34E-23 |
| METTL3 | AC097376.3 | 0.515628 | 5.50E-36 |
| YTHDC1 | AC097376.3 | 0.549127 | 1.66E-41 |
| RBMX | AC097376.3 | 0.445746 | 2.90E-26 |
| RBMX | AC097639.1 | 0.422898 | 1.52E-23 |
| YTHDF1 | AC098820.2 | 0.409807 | 4.48E-22 |
| ALKBH5 | AC098820.2 | 0.414831 | 1.25E-22 |
| METTL3 | AC102953.2 | 0.472634 | 9.64E-30 |
| ALKBH5 | AC102953.2 | 0.422631 | 1.64E-23 |
| ALKBH5 | AC103957.2 | 0.422598 | 1.65E-23 |
| ALKBH5 | AC104078.1 | 0.411817 | 2.69E-22 |
| ALKBH5 | AC104211.2 | 0.431584 | 1.49E-24 |
| YTHDF1 | AC104825.1 | 0.464889 | 1.04E-28 |
| ALKBH5 | AC104825.1 | 0.48337 | 3.21E-31 |
| HNRNPC | AC106795.2 | -0.40955 | 4.78E-22 |
| ALKBH5 | AC106795.2 | 0.415286 | 1.11E-22 |
| YTHDF1 | AC106820.4 | 0.429773 | 2.43E-24 |
| ALKBH5 | AC106820.4 | 0.405833 | 1.21E-21 |
| FTO | AC106820.5 | 0.496591 | 4.12E-33 |
| ALKBH5 | AC106820.5 | 0.491255 | 2.44E-32 |
| METTL3 | AC107068.1 | 0.488559 | 5.94E-32 |
| METTL14 | AC107068.1 | 0.47877 | 1.40E-30 |
| YTHDC1 | AC107068.1 | 0.535938 | 2.93E-39 |
| YTHDC2 | AC107068.1 | 0.48572 | 1.50E-31 |
| YTHDF1 | AC107068.1 | 0.402419 | 2.83E-21 |
| HNRNPA2B1 | AC107068.1 | 0.53877 | 9.83E-40 |
| FTO | AC108472.1 | 0.452002 | 4.79E-27 |
| ALKBH5 | AC108472.1 | 0.40261 | 2.70E-21 |
| METTL3 | AC114956.1 | 0.436436 | 3.94E-25 |
| YTHDF1 | AC120498.3 | 0.425503 | 7.64E-24 |
| YTHDF1 | AC124045.1 | 0.470668 | 1.77E-29 |
| HNRNPA2B1 | AC124045.1 | 0.445626 | 3.00E-26 |
| ALKBH5 | AC125257.1 | 0.543901 | 1.32E-40 |
| ALKBH5 | AC125603.3 | 0.516027 | 4.76E-36 |
| FTO | AC125807.2 | 0.58725 | 1.34E-48 |
| ALKBH5 | AC125807.2 | 0.482374 | 4.43E-31 |
| LRPPRC | AC126773.4 | -0.45419 | 2.52E-27 |
| ALKBH5 | AC127070.1 | 0.439743 | 1.57E-25 |
| LRPPRC | AC137932.2 | -0.40438 | 1.74E-21 |
| RBMX | AC138150.2 | 0.410529 | 3.73E-22 |
| IGFBP3 | AC138649.1 | 0.424875 | 9.03E-24 |
| METTL3 | AC231981.1 | 0.532238 | 1.20E-38 |
| YTHDC1 | AC231981.1 | 0.487815 | 7.58E-32 |
| HNRNPA2B1 | AC231981.1 | 0.413823 | 1.61E-22 |
| WTAP | ADAMTS9-AS2 | 0.408549 | 6.15E-22 |
| YTHDC1 | ADAMTS9-AS2 | 0.49103 | 2.63E-32 |
| HNRNPC | ADAMTS9-AS2 | 0.416177 | 8.81E-23 |
| FMR1 | ADAMTS9-AS2 | 0.600283 | 3.02E-51 |
| RBMX | ADAMTS9-AS2 | 0.624432 | 1.74E-56 |
| METTL14 | AF131215.5 | 0.54017 | 5.71E-40 |
| VIRMA | AF131215.5 | 0.592062 | 1.46E-49 |
| ZC3H13 | AF131215.5 | 0.520517 | 9.38E-37 |
| YTHDC1 | AF131215.5 | 0.52431 | 2.33E-37 |
| YTHDC2 | AF131215.5 | 0.412273 | 2.40E-22 |
| YTHDF3 | AF131215.5 | 0.557771 | 4.91E-43 |
| FMR1 | AF131215.5 | 0.506175 | 1.55E-34 |
| FTO | AF131215.5 | 0.460874 | 3.49E-28 |
| ALKBH5 | AF131216.3 | 0.432492 | 1.16E-24 |
| ALKBH5 | AL023583.1 | 0.423818 | 1.20E-23 |
| METTL3 | AL031775.2 | 0.507946 | 8.35E-35 |
| RBM15B | AL031985.3 | 0.498032 | 2.53E-33 |
| HNRNPC | AL031985.3 | 0.436286 | 4.11E-25 |
| RBMX | AL031985.3 | 0.404843 | 1.55E-21 |
| ALKBH5 | AL033519.4 | 0.400231 | 4.84E-21 |
| IGFBP3 | AL034346.1 | 0.442552 | 7.16E-26 |
| YTHDF1 | AL035587.1 | 0.411571 | 2.86E-22 |
| ALKBH5 | AL035701.1 | 0.421195 | 2.39E-23 |
| METTL3 | AL049780.2 | 0.496379 | 4.42E-33 |
| YTHDC1 | AL049780.2 | 0.426858 | 5.32E-24 |
| ALKBH5 | AL049780.2 | 0.4054 | 1.35E-21 |
| IGFBP3 | AL079303.1 | 0.413269 | 1.86E-22 |
| ALKBH5 | AL118505.1 | 0.46404 | 1.35E-28 |
| ALKBH5 | AL121574.1 | 0.474722 | 5.02E-30 |
| YTHDF1 | AL121839.2 | 0.405177 | 1.43E-21 |
| ALKBH5 | AL132639.2 | 0.457236 | 1.03E-27 |
| METTL3 | AL136980.1 | 0.438978 | 1.95E-25 |
| YTHDC1 | AL137003.1 | 0.467861 | 4.21E-29 |
| YTHDC2 | AL137003.1 | 0.428775 | 3.18E-24 |
| YTHDF1 | AL138999.1 | 0.471718 | 1.28E-29 |
| METTL3 | AL139288.1 | 0.434315 | 7.07E-25 |
| METTL3 | AL157400.3 | 0.450997 | 6.41E-27 |
| METTL3 | AL158196.1 | 0.462257 | 2.31E-28 |
| METTL14 | AL158212.3 | 0.511301 | 2.57E-35 |
| YTHDC1 | AL158212.3 | 0.423724 | 1.23E-23 |
| FTO | AL158212.3 | 0.461689 | 2.74E-28 |
| METTL3 | AL162385.2 | 0.440824 | 1.16E-25 |
| YTHDC2 | AL162385.2 | 0.463292 | 1.69E-28 |
| FTO | AL353150.1 | -0.42296 | 1.50E-23 |
| ALKBH5 | AL354920.1 | 0.479863 | 9.89E-31 |
| FTO | AL355512.1 | -0.48313 | 3.47E-31 |
| ALKBH5 | AL355607.1 | 0.413961 | 1.56E-22 |
| METTL3 | AL356512.1 | 0.424873 | 9.03E-24 |
| YTHDC1 | AL356599.1 | 0.447421 | 1.79E-26 |
| FTO | AL356599.1 | 0.473195 | 8.09E-30 |
| ALKBH5 | AL359532.1 | 0.532848 | 9.53E-39 |
| METTL3 | AL359715.2 | 0.577839 | 9.22E-47 |
| ALKBH5 | AL359962.1 | 0.450338 | 7.75E-27 |
| METTL3 | AL391807.1 | 0.490425 | 3.21E-32 |
| METTL3 | AL512303.1 | 0.423262 | 1.38E-23 |
| METTL3 | AL512306.2 | 0.423507 | 1.30E-23 |
| YTHDF1 | AL513534.3 | 0.412946 | 2.02E-22 |
| RBMX | AL589765.5 | 0.473977 | 6.34E-30 |
| METTL3 | AL592211.1 | 0.524418 | 2.24E-37 |
| LRPPRC | AL592211.1 | -0.4214 | 2.26E-23 |
| METTL3 | AL592435.1 | 0.526 | 1.25E-37 |
| METTL3 | AP000442.1 | 0.44671 | 2.20E-26 |
| ALKBH5 | AP000802.1 | 0.501779 | 7.06E-34 |
| ALKBH5 | AP000866.2 | 0.401237 | 3.78E-21 |
| METTL3 | AP001029.2 | 0.483514 | 3.07E-31 |
| YTHDF1 | AP001085.1 | 0.417466 | 6.31E-23 |
| FTO | AP001271.1 | 0.413662 | 1.68E-22 |
| METTL3 | AP001453.4 | 0.447553 | 1.73E-26 |
| METTL14 | AP001486.2 | 0.508912 | 5.95E-35 |
| VIRMA | AP001486.2 | 0.414332 | 1.42E-22 |
| YTHDC1 | AP001486.2 | 0.498212 | 2.38E-33 |
| YTHDF3 | AP001486.2 | 0.416082 | 9.03E-23 |
| FTO | AP001486.2 | 0.434097 | 7.50E-25 |
| ALKBH5 | AP001486.2 | 0.436676 | 3.69E-25 |
| YTHDF1 | AP001599.1 | 0.433756 | 8.23E-25 |
| ALKBH5 | AP001830.1 | 0.448504 | 1.32E-26 |
| METTL3 | AP002026.1 | 0.537872 | 1.39E-39 |
| YTHDC2 | AP002026.1 | 0.400282 | 4.78E-21 |
| FTO | AP003174.1 | -0.44155 | 9.48E-26 |
| ALKBH5 | AP003721.4 | 0.523143 | 3.59E-37 |
| METTL3 | AP004608.1 | 0.418422 | 4.92E-23 |
| YTHDC1 | ATP2B1-AS1 | 0.404124 | 1.86E-21 |
| HNRNPA2B1 | ATP2B1-AS1 | 0.468889 | 3.07E-29 |
| METTL3 | BCDIN3D-AS1 | 0.459203 | 5.75E-28 |
| FTO | BCDIN3D-AS1 | 0.447328 | 1.84E-26 |
| ALKBH5 | BCDIN3D-AS1 | 0.616173 | 1.21E-54 |
| METTL3 | BDNF-AS | 0.501372 | 8.12E-34 |
| METTL14 | BDNF-AS | 0.45985 | 4.74E-28 |
| YTHDC1 | BDNF-AS | 0.5519 | 5.42E-42 |
| HNRNPA2B1 | BDNF-AS | 0.520725 | 8.70E-37 |
| RBMX | BDNF-AS | 0.439609 | 1.63E-25 |
| ALKBH5 | BDNF-AS | 0.424883 | 9.01E-24 |
| METTL3 | BET1-AS1 | 0.610997 | 1.63E-53 |
| YTHDC2 | BET1-AS1 | 0.44884 | 1.19E-26 |
| HNRNPA2B1 | BET1-AS1 | 0.483309 | 3.28E-31 |
| YTHDC1 | C21orf62-AS1 | 0.416173 | 8.82E-23 |
| METTL3 | C5orf67 | 0.463103 | 1.79E-28 |
| RBMX | C5orf67 | 0.474605 | 5.21E-30 |
| IGFBP3 | C8orf37-AS1 | 0.553949 | 2.36E-42 |
| METTL14 | CASC2 | 0.403742 | 2.04E-21 |
| FTO | CASC2 | 0.473473 | 7.42E-30 |
| ALKBH5 | CASC2 | 0.617894 | 5.06E-55 |
| YTHDF1 | CATIP-AS2 | 0.440897 | 1.14E-25 |
| YTHDC1 | CAVIN2-AS1 | 0.467378 | 4.88E-29 |
| METTL3 | CCDC13-AS1 | 0.405856 | 1.21E-21 |
| ALKBH5 | CCDC13-AS1 | 0.426977 | 5.16E-24 |
| ALKBH5 | CEBPA-DT | 0.506378 | 1.44E-34 |
| METTL3 | CHROMR | 0.455441 | 1.75E-27 |
| YTHDC1 | CHROMR | 0.408606 | 6.06E-22 |
| YTHDC2 | CHROMR | 0.450532 | 7.33E-27 |
| HNRNPA2B1 | CHROMR | 0.438906 | 1.99E-25 |
| METTL3 | COX10-AS1 | 0.573536 | 6.10E-46 |
| YTHDC1 | COX10-AS1 | 0.531618 | 1.52E-38 |
| YTHDC2 | COX10-AS1 | 0.467338 | 4.94E-29 |
| HNRNPA2B1 | COX10-AS1 | 0.446531 | 2.32E-26 |
| FMR1 | CYP1B1-AS1 | 0.492926 | 1.40E-32 |
| ZC3H13 | CYTOR | -0.41575 | 9.84E-23 |
| FTO | CYTOR | -0.47135 | 1.44E-29 |
| ALKBH5 | DANCR | 0.4072 | 8.63E-22 |
| ALKBH5 | DLEU1 | 0.40966 | 4.65E-22 |
| RBMX | DLGAP1-AS2 | 0.447416 | 1.80E-26 |
| FTO | DNAJC3-DT | 0.416912 | 7.28E-23 |
| ALKBH5 | DNAJC3-DT | 0.514918 | 7.09E-36 |
| ALKBH5 | DOCK8-AS1 | 0.410532 | 3.73E-22 |
| RBMX | DOCK9-DT | 0.430532 | 1.98E-24 |
| ALKBH5 | DPH6-DT | 0.542421 | 2.37E-40 |
| FTO | DUBR | 0.411775 | 2.72E-22 |
| ALKBH5 | DYRK3-AS1 | 0.427456 | 4.53E-24 |
| METTL3 | EIF2AK3-DT | 0.442483 | 7.30E-26 |
| YTHDC1 | EIF2AK3-DT | 0.459358 | 5.49E-28 |
| FTO | EIF2AK3-DT | 0.411533 | 2.89E-22 |
| ALKBH5 | EIF2AK3-DT | 0.476564 | 2.81E-30 |
| METTL3 | ELOA-AS1 | 0.485266 | 1.74E-31 |
| ALKBH5 | ELOA-AS1 | 0.435059 | 5.76E-25 |
| ALKBH5 | FAM182B | 0.493786 | 1.05E-32 |
| ALKBH5 | FAM201A | 0.539427 | 7.62E-40 |
| RBM15B | FIGNL2-DT | 0.428847 | 3.12E-24 |
| RBMX | FIGNL2-DT | 0.512151 | 1.90E-35 |
| ALKBH5 | FZD4-DT | 0.410548 | 3.71E-22 |
| YTHDF1 | GIHCG | 0.418333 | 5.04E-23 |
| ALKBH5 | GIHCG | 0.494704 | 7.75E-33 |
| METTL3 | H1FX-AS1 | 0.400708 | 4.31E-21 |
| LRPPRC | H1FX-AS1 | -0.44434 | 4.32E-26 |
| ALKBH5 | IQCH-AS1 | 0.483257 | 3.33E-31 |
| YTHDC1 | ITGA9-AS1 | 0.533294 | 8.05E-39 |
| RBMX | ITGA9-AS1 | 0.524343 | 2.30E-37 |
| ALKBH5 | ITPR1-DT | 0.459532 | 5.21E-28 |
| ALKBH5 | LCMT1-AS1 | 0.439664 | 1.61E-25 |
| ALKBH5 | LIFR-AS1 | 0.580291 | 3.10E-47 |
| YTHDF1 | LINC00205 | 0.484656 | 2.12E-31 |
| ALKBH5 | LINC00205 | 0.458574 | 6.93E-28 |
| ALKBH5 | LINC00629 | 0.480737 | 7.48E-31 |
| METTL3 | LINC00667 | 0.476766 | 2.64E-30 |
| YTHDC1 | LINC00667 | 0.49833 | 2.29E-33 |
| YTHDC2 | LINC00667 | 0.41632 | 8.49E-23 |
| VIRMA | LINC00853 | -0.45708 | 1.08E-27 |
| ZC3H13 | LINC00853 | -0.46768 | 4.44E-29 |
| YTHDF3 | LINC00853 | -0.45339 | 3.19E-27 |
| FTO | LINC00853 | -0.44162 | 9.30E-26 |
| FTO | LINC00886 | 0.426887 | 5.28E-24 |
| ALKBH5 | LINC00886 | 0.588396 | 7.92E-49 |
| FMR1 | LINC00891 | 0.481602 | 5.67E-31 |
| ALKBH5 | LINC00940 | 0.452475 | 4.17E-27 |
| FTO | LINC01135 | 0.485205 | 1.77E-31 |
| ALKBH5 | LINC01135 | 0.454772 | 2.13E-27 |
| VIRMA | LINC01137 | -0.43731 | 3.09E-25 |
| ZC3H13 | LINC01137 | -0.48789 | 7.40E-32 |
| YTHDF3 | LINC01137 | -0.40817 | 6.76E-22 |
| FTO | LINC01137 | -0.4493 | 1.05E-26 |
| ALKBH5 | LINC01144 | 0.474175 | 5.96E-30 |
| ALKBH5 | LINC01152 | 0.437586 | 2.87E-25 |
| ALKBH5 | LINC01184 | 0.42508 | 8.55E-24 |
| IGFBP3 | LINC01224 | 0.482637 | 4.07E-31 |
| YTHDF1 | LINC01270 | 0.437362 | 3.05E-25 |
| ALKBH5 | LINC01270 | 0.427303 | 4.72E-24 |
| METTL3 | LINC01473 | 0.452911 | 3.67E-27 |
| RBMX | LINC01473 | 0.421494 | 2.21E-23 |
| ALKBH5 | LINC01508 | 0.475002 | 4.60E-30 |
| ALKBH5 | LINC01550 | 0.516319 | 4.29E-36 |
| ALKBH5 | LINC01571 | 0.42943 | 2.67E-24 |
| ALKBH5 | LINC01586 | 0.550835 | 8.33E-42 |
| YTHDF1 | LINC01607 | 0.431774 | 1.41E-24 |
| ALKBH5 | LINC01644 | -0.40559 | 1.29E-21 |
| METTL3 | LINC01786 | 0.480415 | 8.29E-31 |
| METTL3 | LINC01844 | 0.463296 | 1.69E-28 |
| YTHDF1 | LINC01844 | 0.403818 | 2.00E-21 |
| IGFBP3 | LINC01844 | 0.434944 | 5.95E-25 |
| RBMX | LINC01918 | 0.472002 | 1.17E-29 |
| ALKBH5 | LINC01975 | 0.42141 | 2.26E-23 |
| ALKBH5 | LINC02028 | 0.499232 | 1.68E-33 |
| ALKBH5 | LINC02447 | 0.402079 | 3.08E-21 |
| FMR1 | LINC02454 | 0.400855 | 4.15E-21 |
| ZC3H13 | LINC02560 | -0.41991 | 3.34E-23 |
| FTO | LINC02560 | -0.51609 | 4.65E-36 |
| METTL3 | LINC02848 | 0.421448 | 2.23E-23 |
| VIRMA | LNCSRLR | 0.450092 | 8.33E-27 |
| ZC3H13 | LNCSRLR | 0.498339 | 2.28E-33 |
| YTHDF3 | LNCSRLR | 0.474138 | 6.03E-30 |
| ALKBH5 | LOH12CR2 | 0.59395 | 6.04E-50 |
| ALKBH5 | LRIG2-DT | 0.48405 | 2.58E-31 |
| FMR1 | LRRC52-AS1 | 0.401118 | 3.89E-21 |
| RBMX | LRRC52-AS1 | 0.430017 | 2.28E-24 |
| METTL14 | LYPLAL1-DT | 0.476481 | 2.89E-30 |
| YTHDC1 | LYPLAL1-DT | 0.419825 | 3.42E-23 |
| FTO | LYPLAL1-DT | 0.443234 | 5.90E-26 |
| ALKBH5 | LYPLAL1-DT | 0.415368 | 1.08E-22 |
| HNRNPC | MIR100HG | 0.413387 | 1.80E-22 |
| RBMX | MIR100HG | 0.55091 | 8.08E-42 |
| RBMX | MIR181A2HG | 0.413662 | 1.68E-22 |
| FTO | MKLN1-AS | 0.494488 | 8.33E-33 |
| ALKBH5 | MKLN1-AS | 0.524177 | 2.45E-37 |
| YTHDC1 | MRPS30-DT | 0.430398 | 2.05E-24 |
| ALKBH5 | NCK1-DT | 0.428572 | 3.36E-24 |
| YTHDC1 | NOP14-AS1 | 0.412974 | 2.00E-22 |
| RBMX | NOP14-AS1 | 0.450803 | 6.78E-27 |
| ALKBH5 | NR2F1-AS1 | -0.45515 | 1.91E-27 |
| ALKBH5 | NRSN2-AS1 | 0.470336 | 1.96E-29 |
| METTL3 | OLMALINC | 0.501955 | 6.65E-34 |
| RBMX | OLMALINC | 0.518211 | 2.17E-36 |
| METTL3 | OR2A1-AS1 | 0.415222 | 1.13E-22 |
| YTHDC1 | OR2A1-AS1 | 0.427049 | 5.06E-24 |
| YTHDC2 | OR2A1-AS1 | 0.416084 | 9.02E-23 |
| FTO | OR2A1-AS1 | 0.4299 | 2.35E-24 |
| METTL14 | OTUD6B-AS1 | 0.632385 | 2.59E-58 |
| VIRMA | OTUD6B-AS1 | 0.492848 | 1.44E-32 |
| ZC3H13 | OTUD6B-AS1 | 0.484875 | 1.97E-31 |
| YTHDC1 | OTUD6B-AS1 | 0.574834 | 3.46E-46 |
| YTHDF3 | OTUD6B-AS1 | 0.447678 | 1.67E-26 |
| HNRNPA2B1 | OTUD6B-AS1 | 0.424229 | 1.07E-23 |
| FTO | OTUD6B-AS1 | 0.527601 | 6.88E-38 |
| ALKBH5 | OTUD6B-AS1 | 0.422983 | 1.49E-23 |
| YTHDC1 | PAX8-AS1 | 0.415917 | 9.42E-23 |
| YTHDC2 | PAX8-AS1 | 0.511164 | 2.69E-35 |
| FTO | PAX8-AS1 | 0.441686 | 9.13E-26 |
| IGFBP3 | PRKAG2-AS1 | 0.410387 | 3.87E-22 |
| ALKBH5 | PRKCQ-AS1 | 0.44227 | 7.75E-26 |
| ALKBH5 | PROX1-AS1 | 0.491058 | 2.61E-32 |
| LRPPRC | RARA-AS1 | -0.51845 | 1.99E-36 |
| METTL3 | RASSF8-AS1 | 0.454262 | 2.47E-27 |
| METTL3 | RBM26-AS1 | 0.467149 | 5.23E-29 |
| YTHDC1 | RBM26-AS1 | 0.437617 | 2.84E-25 |
| FTO | RBM26-AS1 | 0.417102 | 6.93E-23 |
| METTL3 | SAP30-DT | 0.433876 | 7.97E-25 |
| METTL3 | SDCBP2-AS1 | 0.530733 | 2.12E-38 |
| FTO | SDCBP2-AS1 | 0.460084 | 4.42E-28 |
| ALKBH5 | SDCBP2-AS1 | 0.506957 | 1.18E-34 |
| ALKBH5 | SEPTIN4-AS1 | 0.449818 | 9.01E-27 |
| ALKBH5 | SGMS1-AS1 | 0.439707 | 1.59E-25 |
| ALKBH5 | SLC25A5-AS1 | 0.582217 | 1.31E-47 |
| ALKBH5 | SLC26A4-AS1 | 0.532228 | 1.21E-38 |
| ALKBH5 | SNHG30 | 0.407798 | 7.43E-22 |
| RBMX | SNHG7 | 0.403088 | 2.40E-21 |
| HNRNPA2B1 | SOS1-IT1 | 0.449753 | 9.18E-27 |
| ALKBH5 | SOX9-AS1 | 0.504669 | 2.61E-34 |
| METTL3 | SRP14-AS1 | 0.508163 | 7.74E-35 |
| ALKBH5 | SRP14-AS1 | 0.531706 | 1.47E-38 |
| METTL3 | ST3GAL5-AS1 | 0.451753 | 5.15E-27 |
| WTAP | ST3GAL5-AS1 | 0.407375 | 8.26E-22 |
| YTHDC1 | ST3GAL5-AS1 | 0.507344 | 1.03E-34 |
| FMR1 | ST3GAL5-AS1 | 0.429311 | 2.75E-24 |
| RBMX | ST3GAL5-AS1 | 0.519512 | 1.35E-36 |
| ALKBH5 | ST7-AS1 | 0.482678 | 4.01E-31 |
| FTO | STX18-AS1 | 0.439524 | 1.67E-25 |
| ALKBH5 | STX18-AS1 | 0.511722 | 2.21E-35 |
| ALKBH5 | SYNE1-AS1 | 0.481599 | 5.68E-31 |
| YTHDC1 | TAPT1-AS1 | 0.401538 | 3.51E-21 |
| ALKBH5 | TBC1D8-AS1 | 0.55627 | 9.11E-43 |
| METTL3 | TCERG1L-AS1 | 0.441897 | 8.61E-26 |
| RBMX | TCERG1L-AS1 | 0.422749 | 1.59E-23 |
| METTL3 | TMCC1-AS1 | 0.48319 | 3.40E-31 |
| ALKBH5 | TMEM220-AS1 | 0.453803 | 2.83E-27 |
| ALKBH5 | TONSL-AS1 | 0.55654 | 8.15E-43 |
| METTL3 | UBR5-AS1 | 0.514122 | 9.42E-36 |
| YTHDC1 | UBR5-AS1 | 0.465921 | 7.61E-29 |
| FTO | UNC5B-AS1 | -0.4363 | 4.10E-25 |
| METTL3 | USP3-AS1 | 0.443117 | 6.10E-26 |
| ALKBH5 | USP3-AS1 | 0.435613 | 4.95E-25 |
| METTL14 | USP46-AS1 | 0.426873 | 5.30E-24 |
| VIRMA | USP46-AS1 | 0.449427 | 1.01E-26 |
| ZC3H13 | USP46-AS1 | 0.467782 | 4.31E-29 |
| YTHDC2 | USP46-AS1 | 0.400792 | 4.22E-21 |
| YTHDF3 | USP46-AS1 | 0.463773 | 1.46E-28 |
| LRPPRC | USP46-AS1 | 0.449086 | 1.11E-26 |
| FTO | USP46-AS1 | 0.516009 | 4.79E-36 |
| METTL14 | UXT-AS1 | 0.444457 | 4.18E-26 |
| ZC3H13 | UXT-AS1 | 0.410832 | 3.46E-22 |
| YTHDC1 | UXT-AS1 | 0.505151 | 2.21E-34 |
| FTO | UXT-AS1 | 0.509146 | 5.48E-35 |
| METTL14 | WDFY3-AS2 | 0.416664 | 7.77E-23 |
| FTO | WDFY3-AS2 | 0.516768 | 3.65E-36 |
| ALKBH5 | WDFY3-AS2 | 0.517344 | 2.97E-36 |
| ALKBH5 | WWTR1-AS1 | 0.531156 | 1.81E-38 |
| ALKBH5 | Z95115.1 | 0.451838 | 5.02E-27 |
| METTL3 | Z98884.2 | 0.500639 | 1.04E-33 |
| METTL3 | ZBED3-AS1 | 0.480192 | 8.90E-31 |
| ALKBH5 | ZBED3-AS1 | 0.431836 | 1.39E-24 |
| ALKBH5 | ZBED5-AS1 | 0.561937 | 8.67E-44 |
| METTL3 | ZNF674-AS1 | 0.532145 | 1.24E-38 |
| YTHDC1 | ZNF674-AS1 | 0.417945 | 5.57E-23 |
| ALKBH5 | ZNF674-AS1 | 0.403194 | 2.34E-21 |
| ALKBH5 | ZNF710-AS1 | 0.48536 | 1.69E-31 |
| IGFBP3 | ZRANB2-AS2 | 0.739366 | 2.57E-89 |
| VIRMA | ZSCAN16-AS1 | -0.41809 | 5.36E-23 |
| ZC3H13 | ZSCAN16-AS1 | -0.47975 | 1.03E-30 |
| FMR1 | ZSCAN16-AS1 | -0.44714 | 1.94E-26 |

**Table S4.** Cox analysis of 70 m^6^A-associated lncRNAs in THCA patients.

| **Gene** | **HR** | **HR.95L** | **HR.95H** | **P-value** |
| --- | --- | --- | --- | --- |
| AC007365.1 | 7.3281 | 2.6591 | 20.1948 | 0.0001 |
| STX18.AS1 | 135.8049 | 10.3982 | 1773.665 | 0.0002 |
| AC102953.2 | 3.5975 | 1.8193 | 7.1139 | 0.0002 |
| SGMS1.AS1 | 9.5622 | 2.8491 | 32.0934 | 0.0003 |
| SAP30.DT | 5.0804 | 2.1174 | 12.1895 | 0.0003 |
| LINC00667 | 2.0476 | 1.3728 | 3.054 | 0.0004 |
| PROX1.AS1 | 27.5561 | 4.2346 | 179.3174 | 0.0005 |
| DYRK3.AS1 | 18.5172 | 3.4925 | 98.1781 | 0.0006 |
| LINC02028 | 53.6934 | 4.9371 | 583.9463 | 0.0011 |
| AC004825.2 | 1.9423 | 1.3019 | 2.8976 | 0.0011 |
| AC106820.4 | 7.0971 | 2.1462 | 23.4692 | 0.0013 |
| DPH6.DT | 6.2716 | 1.9939 | 19.7261 | 0.0017 |
| AC106820.5 | 12.2017 | 2.5537 | 58.3006 | 0.0017 |
| IQCH.AS1 | 4.0987 | 1.6441 | 10.2179 | 0.0025 |
| AL139288.1 | 7.3653 | 1.9555 | 27.7408 | 0.0032 |
| AC011700.1 | 9.4139 | 2.1196 | 41.8104 | 0.0032 |
| AC040160.1 | 1.2667 | 1.0813 | 1.4838 | 0.0034 |
| AC019080.1 | 131.1804 | 4.9043 | 3508.833 | 0.0036 |
| A2M.AS1 | 4.4503 | 1.5818 | 12.5204 | 0.0047 |
| AC064807.1 | 2.1681 | 1.2546 | 3.7467 | 0.0056 |
| LINC01184 | 1.966 | 1.2159 | 3.1789 | 0.0058 |
| AC005034.5 | 2.6936 | 1.3281 | 5.4631 | 0.006 |
| LINC01975 | 2.6079 | 1.3124 | 5.1821 | 0.0062 |
| AC079848.1 | 2.8485 | 1.3062 | 6.212 | 0.0085 |
| AC125807.2 | 1.7729 | 1.1569 | 2.7171 | 0.0086 |
| LYPLAL1.DT | 32.9198 | 2.2487 | 481.9202 | 0.0107 |
| EIF2AK3.DT | 9.9604 | 1.6968 | 58.4671 | 0.0109 |
| AC067750.1 | 3.6098 | 1.3434 | 9.6999 | 0.0109 |
| ST7.AS1 | 1.6689 | 1.1231 | 2.4799 | 0.0113 |
| AC010980.2 | 1.3758 | 1.071 | 1.7673 | 0.0125 |
| AC046143.1 | 0.2015 | 0.0569 | 0.7133 | 0.013 |
| AC104825.1 | 1.2528 | 1.047 | 1.4991 | 0.0138 |
| AC114956.1 | 9.6755 | 1.5845 | 59.0816 | 0.0139 |
| TMEM220.AS1 | 2.2829 | 1.163 | 4.4814 | 0.0165 |
| AC008738.2 | 3.6344 | 1.2609 | 10.4757 | 0.0169 |
| AC006213.1 | 15.6074 | 1.6364 | 148.8608 | 0.0169 |
| DOCK9.DT | 0.8993 | 0.824 | 0.9814 | 0.0173 |
| AL023583.1 | 4.3157 | 1.2844 | 14.5012 | 0.018 |
| AP001271.1 | 4.8187 | 1.2991 | 17.8737 | 0.0187 |
| AC108472.1 | 8.6613 | 1.4184 | 52.8889 | 0.0194 |
| AC091965.1 | 8.6155 | 1.369 | 54.2211 | 0.0218 |
| WDFY3.AS2 | 2.1514 | 1.1085 | 4.1753 | 0.0235 |
| UXT.AS1 | 25.7814 | 1.5472 | 429.5917 | 0.0236 |
| DLGAP1.AS2 | 1.2531 | 1.0302 | 1.5243 | 0.024 |
| LIFR.AS1 | 1.7779 | 1.0779 | 2.9325 | 0.0242 |
| AL359532.1 | 4.9293 | 1.2208 | 19.9035 | 0.0251 |
| PAX8.AS1 | 2.6244 | 1.1252 | 6.1212 | 0.0256 |
| CASC2 | 4.2864 | 1.1825 | 15.5377 | 0.0268 |
| TONSL.AS1 | 1.7106 | 1.063 | 2.7528 | 0.027 |
| AL512306.2 | 6.353 | 1.2331 | 32.7321 | 0.0271 |
| LINC02454 | 0.6313 | 0.4188 | 0.9517 | 0.028 |
| ITPR1.DT | 1.5907 | 1.0473 | 2.4161 | 0.0295 |
| LINC00205 | 1.1717 | 1.0153 | 1.3522 | 0.0301 |
| TBC1D8.AS1 | 3.108 | 1.1149 | 8.6637 | 0.0302 |
| AC008555.1 | 0.0636 | 0.0052 | 0.7708 | 0.0304 |
| AP001453.4 | 2.5844 | 1.0781 | 6.1951 | 0.0333 |
| MKLN1.AS | 5.2044 | 1.1384 | 23.7932 | 0.0334 |
| MIR181A2HG | 0.83 | 0.6982 | 0.9866 | 0.0346 |
| LINC01144 | 1.4731 | 1.0284 | 2.11 | 0.0346 |
| MRPS30.DT | 7.5359 | 1.1282 | 50.3369 | 0.0371 |
| AP000802.1 | 8.6192 | 1.1368 | 65.3509 | 0.0372 |
| FAM182B | 7.4717 | 1.1088 | 50.3485 | 0.0388 |
| AC126773.4 | 0.1957 | 0.0414 | 0.9243 | 0.0395 |
| LCMT1.AS1 | 7.8504 | 1.1013 | 55.9588 | 0.0398 |
| LINC02447 | 3.7168 | 1.0542 | 13.1039 | 0.0411 |
| AC004540.1 | 1.2254 | 1.005 | 1.4941 | 0.0445 |
| AC103957.2 | 1.2588 | 1.0032 | 1.5796 | 0.0469 |
| AC096920.1 | 13.5952 | 1.0308 | 179.307 | 0.0474 |
| ADAMTS9.AS2 | 0.2073 | 0.0436 | 0.9854 | 0.0479 |
| AC023510.2 | 2.3571 | 1.0032 | 5.5384 | 0.0492 |

**Table S5.** The clinicopathological characteristics of THCA patients in different clusters.

| **Id** | **Cluster** | **Age** | **Gender** | **Stage** | **T** | **M** | **N** |
| --- | --- | --- | --- | --- | --- | --- | --- |
| TCGA-4C-A93U | Cluster1 | >65 | FEMALE | Stage III-IV | T3-4 | M0 | N1b |
| TCGA-BJ-A0YZ | Cluster1 | <=65 | MALE | Stage I-II | T1-2 | M0 | N0 |
| TCGA-BJ-A0Z0 | Cluster1 | <=65 | MALE | Stage I-II | T1-2 | unknow | N0 |
| TCGA-BJ-A0Z9 | Cluster1 | <=65 | FEMALE | Stage III-IV | T3-4 | unknow | N0 |
| TCGA-BJ-A0ZA | Cluster1 | >65 | FEMALE | Stage I-II | T1-2 | M0 | N0 |
| TCGA-BJ-A0ZC | Cluster1 | <=65 | MALE | Stage I-II | T1-2 | M0 | N0 |
| TCGA-BJ-A0ZE | Cluster1 | <=65 | FEMALE | Stage III-IV | T3-4 | M0 | N0 |
| TCGA-BJ-A0ZF | Cluster1 | <=65 | FEMALE | Stage I-II | T1-2 | M0 | N0 |
| TCGA-BJ-A0ZG | Cluster1 | >65 | MALE | Stage I-II | T1-2 | M0 | N0 |
| TCGA-BJ-A0ZJ | Cluster1 | <=65 | MALE | Stage I-II | T1-2 | M0 | N1a |
| TCGA-BJ-A18Y | Cluster1 | <=65 | MALE | Stage I-II | T1-2 | unknow | N0 |
| TCGA-BJ-A190 | Cluster1 | <=65 | MALE | Stage I-II | T1-2 | unknow | N0 |
| TCGA-BJ-A191 | Cluster1 | <=65 | FEMALE | Stage I-II | T1-2 | M0 | N0 |
| TCGA-BJ-A192 | Cluster1 | <=65 | FEMALE | Stage III-IV | T3-4 | M0 | NX |
| TCGA-BJ-A28S | Cluster1 | >65 | MALE | Stage I-II | T1-2 | M0 | N0 |
| TCGA-BJ-A28T | Cluster1 | <=65 | FEMALE | Stage I-II | T1-2 | M0 | N1a |
| TCGA-BJ-A28V | Cluster1 | >65 | FEMALE | Stage III-IV | T3-4 | unknow | N0 |
| TCGA-BJ-A28W | Cluster1 | <=65 | FEMALE | Stage I-II | T1-2 | M0 | N0 |
| TCGA-BJ-A28Z | Cluster1 | <=65 | FEMALE | Stage III-IV | T3-4 | M0 | N1a |
| TCGA-BJ-A291 | Cluster1 | <=65 | FEMALE | Stage I-II | T1-2 | M0 | N0 |
| TCGA-BJ-A2N7 | Cluster1 | <=65 | FEMALE | Stage I-II | T3-4 | M0 | N0 |
| TCGA-BJ-A2N9 | Cluster1 | <=65 | FEMALE | Stage I-II | T1-2 | M0 | N0 |
| TCGA-BJ-A2P4 | Cluster1 | <=65 | FEMALE | Stage I-II | T1-2 | M0 | N0 |
| TCGA-BJ-A3F0 | Cluster1 | <=65 | FEMALE | Stage I-II | T1-2 | M0 | N0 |
| TCGA-BJ-A3PT | Cluster1 | <=65 | FEMALE | Stage III-IV | T3-4 | M0 | N0 |
| TCGA-BJ-A45C | Cluster1 | >65 | MALE | Stage III-IV | T3-4 | M0 | N0 |
| TCGA-BJ-A45D | Cluster1 | <=65 | MALE | Stage I-II | T1-2 | M0 | N0 |
| TCGA-BJ-A45E | Cluster1 | <=65 | FEMALE | Stage I-II | T1-2 | M0 | N0 |
| TCGA-BJ-A45F | Cluster1 | <=65 | FEMALE | Stage I-II | T1-2 | M0 | N0 |
| TCGA-BJ-A45G | Cluster1 | <=65 | FEMALE | Stage III-IV | T1-2 | M0 | N1 |
| TCGA-BJ-A45K | Cluster1 | <=65 | MALE | Stage I-II | T1-2 | M0 | N0 |
| TCGA-CE-A27D | Cluster1 | <=65 | FEMALE | Stage I-II | T1-2 | M0 | N1 |
| TCGA-DE-A2OL | Cluster1 | <=65 | FEMALE | Stage I-II | T1-2 | unknow | unknow |
| TCGA-DE-A4M9 | Cluster1 | <=65 | MALE | Stage I-II | T1-2 | M0 | NX |
| TCGA-DE-A4MB | Cluster1 | >65 | FEMALE | Stage III-IV | T3-4 | M0 | N1b |
| TCGA-DJ-A13M | Cluster1 | <=65 | FEMALE | Stage I-II | T1-2 | M0 | N0 |
| TCGA-DJ-A13R | Cluster1 | <=65 | MALE | Stage III-IV | T3-4 | M0 | N0 |
| TCGA-DJ-A13S | Cluster1 | <=65 | FEMALE | Stage I-II | T1-2 | M0 | N0 |
| TCGA-DJ-A13W | Cluster1 | <=65 | FEMALE | Stage I-II | T1-2 | M0 | N0 |
| TCGA-DJ-A1QG | Cluster1 | <=65 | MALE | Stage I-II | T1-2 | M0 | N0 |
| TCGA-DJ-A1QL | Cluster1 | >65 | MALE | Stage I-II | T1-2 | M0 | N0 |
| TCGA-DJ-A1QM | Cluster1 | <=65 | MALE | Stage I-II | T1-2 | M0 | N0 |
| TCGA-DJ-A2PP | Cluster1 | <=65 | MALE | Stage I-II | T1-2 | M0 | N0 |
| TCGA-DJ-A2Q0 | Cluster1 | <=65 | FEMALE | Stage I-II | T1-2 | M0 | N0 |
| TCGA-DJ-A2Q1 | Cluster1 | <=65 | FEMALE | Stage I-II | T1-2 | M0 | N1b |
| TCGA-DJ-A2Q2 | Cluster1 | <=65 | FEMALE | Stage III-IV | T3-4 | M0 | N0 |
| TCGA-DJ-A2Q9 | Cluster1 | <=65 | FEMALE | Stage III-IV | T1-2 | M0 | N1a |
| TCGA-DJ-A2QB | Cluster1 | <=65 | FEMALE | Stage III-IV | T3-4 | M0 | N1b |
| TCGA-DJ-A3US | Cluster1 | <=65 | FEMALE | Stage I-II | T1-2 | M0 | N0 |
| TCGA-DJ-A3UT | Cluster1 | <=65 | FEMALE | Stage I-II | T1-2 | M0 | N0 |
| TCGA-DJ-A3UV | Cluster1 | <=65 | FEMALE | Stage I-II | T1-2 | M0 | N0 |
| TCGA-DJ-A3UY | Cluster1 | <=65 | FEMALE | Stage I-II | T1-2 | M0 | N0 |
| TCGA-DJ-A3VD | Cluster1 | <=65 | FEMALE | Stage I-II | T1-2 | M0 | N1a |
| TCGA-DJ-A3VF | Cluster1 | <=65 | FEMALE | Stage III-IV | T3-4 | M0 | N1b |
| TCGA-DJ-A3VG | Cluster1 | <=65 | MALE | Stage I-II | T1-2 | M0 | N0 |
| TCGA-DJ-A3VL | Cluster1 | <=65 | MALE | Stage I-II | T1-2 | M0 | N0 |
| TCGA-DJ-A3VM | Cluster1 | >65 | FEMALE | Stage I-II | T1-2 | M0 | N0 |
| TCGA-DJ-A4UQ | Cluster1 | <=65 | MALE | Stage III-IV | T3-4 | M0 | N1b |
| TCGA-DJ-A4UR | Cluster1 | <=65 | FEMALE | Stage I-II | T3-4 | M1 | N1b |
| TCGA-DJ-A4V0 | Cluster1 | <=65 | FEMALE | Stage I-II | T1-2 | M0 | N0 |
| TCGA-DO-A2HM | Cluster1 | <=65 | MALE | Stage III-IV | T1-2 | unknow | N1 |
| TCGA-E3-A3DZ | Cluster1 | <=65 | FEMALE | Stage III-IV | T3-4 | M0 | N0 |
| TCGA-E8-A414 | Cluster1 | <=65 | FEMALE | Stage III-IV | T3-4 | M0 | N0 |
| TCGA-E8-A416 | Cluster1 | <=65 | FEMALE | Stage I-II | T1-2 | M0 | N0 |
| TCGA-E8-A434 | Cluster1 | <=65 | FEMALE | Stage I-II | T1-2 | M0 | N0 |
| TCGA-E8-A438 | Cluster1 | <=65 | FEMALE | Stage I-II | T1-2 | M0 | N0 |
| TCGA-E8-A44M | Cluster1 | <=65 | FEMALE | Stage I-II | T1-2 | M0 | N0 |
| TCGA-EL-A3CN | Cluster1 | <=65 | FEMALE | Stage I-II | T3-4 | M0 | N0 |
| TCGA-EL-A3CO | Cluster1 | >65 | MALE | Stage III-IV | T3-4 | M0 | N1b |
| TCGA-EL-A3CS | Cluster1 | <=65 | FEMALE | Stage III-IV | T3-4 | M0 | N0 |
| TCGA-EL-A3CT | Cluster1 | >65 | FEMALE | Stage III-IV | T3-4 | M0 | N1a |
| TCGA-EL-A3CV | Cluster1 | <=65 | MALE | Stage I-II | T3-4 | M0 | N1b |
| TCGA-EL-A3CW | Cluster1 | >65 | FEMALE | Stage III-IV | T1-2 | M0 | N1a |
| TCGA-EL-A3CX | Cluster1 | <=65 | FEMALE | Stage I-II | T1-2 | M0 | N0 |
| TCGA-EL-A3CY | Cluster1 | <=65 | MALE | Stage I-II | unknow | M0 | N1b |
| TCGA-EL-A3CZ | Cluster1 | <=65 | FEMALE | Stage I-II | T1-2 | M0 | N1a |
| TCGA-EL-A3D4 | Cluster1 | <=65 | MALE | Stage III-IV | T3-4 | M0 | N1b |
| TCGA-EL-A3D5 | Cluster1 | <=65 | FEMALE | Stage I-II | T3-4 | M0 | N1b |
| TCGA-EL-A3GO | Cluster1 | <=65 | FEMALE | Stage I-II | T1-2 | M0 | N0 |
| TCGA-EL-A3GP | Cluster1 | >65 | MALE | Stage III-IV | T3-4 | M0 | N1a |
| TCGA-EL-A3GW | Cluster1 | <=65 | FEMALE | Stage I-II | T1-2 | M0 | N0 |
| TCGA-EL-A3H1 | Cluster1 | >65 | FEMALE | Stage I-II | T1-2 | M0 | N0 |
| TCGA-EL-A3H2 | Cluster1 | <=65 | MALE | Stage I-II | T1-2 | unknow | N0 |
| TCGA-EL-A3H3 | Cluster1 | <=65 | FEMALE | Stage I-II | T1-2 | M0 | N1 |
| TCGA-EL-A3H5 | Cluster1 | <=65 | FEMALE | Stage III-IV | T3-4 | M0 | N1 |
| TCGA-EL-A3MY | Cluster1 | >65 | MALE | Stage III-IV | T3-4 | M1 | NX |
| TCGA-EL-A3T1 | Cluster1 | <=65 | FEMALE | Stage I-II | T1-2 | M0 | N0 |
| TCGA-EL-A3T2 | Cluster1 | <=65 | FEMALE | Stage III-IV | T1-2 | M0 | N1 |
| TCGA-EL-A3T9 | Cluster1 | >65 | FEMALE | Stage III-IV | T3-4 | unknow | N1 |
| TCGA-EL-A3ZK | Cluster1 | <=65 | FEMALE | Stage I-II | T1-2 | M0 | N1 |
| TCGA-EL-A3ZO | Cluster1 | >65 | FEMALE | Stage III-IV | T3-4 | M0 | N1 |
| TCGA-EL-A3ZR | Cluster1 | <=65 | FEMALE | Stage I-II | T1-2 | M0 | N0 |
| TCGA-EL-A4JV | Cluster1 | <=65 | FEMALE | Stage I-II | T1-2 | M0 | N0 |
| TCGA-EL-A4JX | Cluster1 | <=65 | FEMALE | Stage I-II | T3-4 | unknow | N0 |
| TCGA-EL-A4K1 | Cluster1 | >65 | FEMALE | Stage III-IV | T3-4 | M0 | N1 |
| TCGA-EL-A4K2 | Cluster1 | <=65 | FEMALE | Stage I-II | T1-2 | M0 | N0 |
| TCGA-EL-A4KD | Cluster1 | <=65 | MALE | Stage I-II | T3-4 | M0 | N1 |
| TCGA-EM-A1CW | Cluster1 | <=65 | FEMALE | Stage I-II | T3-4 | unknow | N0 |
| TCGA-EM-A1YA | Cluster1 | >65 | FEMALE | Stage I-II | T1-2 | unknow | unknow |
| TCGA-EM-A1YB | Cluster1 | >65 | FEMALE | Stage I-II | T1-2 | unknow | unknow |
| TCGA-EM-A1YC | Cluster1 | >65 | FEMALE | Stage III-IV | T3-4 | unknow | N0 |
| TCGA-EM-A1YD | Cluster1 | <=65 | FEMALE | Stage I-II | T1-2 | unknow | N0 |
| TCGA-EM-A1YE | Cluster1 | <=65 | FEMALE | Stage I-II | T1-2 | unknow | N0 |
| TCGA-EM-A22J | Cluster1 | <=65 | FEMALE | Stage I-II | T1-2 | unknow | N0 |
| TCGA-EM-A22K | Cluster1 | <=65 | FEMALE | Stage III-IV | T1-2 | unknow | N1 |
| TCGA-EM-A22L | Cluster1 | <=65 | FEMALE | Stage I-II | T1-2 | unknow | N0 |
| TCGA-EM-A22N | Cluster1 | >65 | FEMALE | Stage I-II | T1-2 | unknow | N0 |
| TCGA-EM-A22Q | Cluster1 | <=65 | MALE | Stage I-II | T3-4 | unknow | N0 |
| TCGA-EM-A2CJ | Cluster1 | <=65 | FEMALE | Stage I-II | T1-2 | unknow | unknow |
| TCGA-EM-A2CK | Cluster1 | <=65 | MALE | Stage I-II | T1-2 | unknow | N1 |
| TCGA-EM-A2CL | Cluster1 | <=65 | MALE | Stage I-II | T1-2 | unknow | unknow |
| TCGA-EM-A2CM | Cluster1 | <=65 | FEMALE | Stage III-IV | T3-4 | unknow | N0 |
| TCGA-EM-A2CO | Cluster1 | >65 | FEMALE | Stage I-II | T1-2 | unknow | unknow |
| TCGA-EM-A2CP | Cluster1 | <=65 | FEMALE | Stage I-II | T1-2 | unknow | N0 |
| TCGA-EM-A2CQ | Cluster1 | <=65 | FEMALE | Stage I-II | T1-2 | unknow | N1 |
| TCGA-EM-A2CR | Cluster1 | <=65 | FEMALE | Stage I-II | T1-2 | unknow | N0 |
| TCGA-EM-A2CT | Cluster1 | <=65 | FEMALE | Stage I-II | T1-2 | unknow | N0 |
| TCGA-EM-A2OV | Cluster1 | <=65 | FEMALE | Stage I-II | T1-2 | unknow | N0 |
| TCGA-EM-A2OW | Cluster1 | <=65 | FEMALE | Stage I-II | T1-2 | unknow | unknow |
| TCGA-EM-A2OY | Cluster1 | <=65 | FEMALE | Stage I-II | T1-2 | unknow | N0 |
| TCGA-EM-A2P1 | Cluster1 | <=65 | MALE | Stage I-II | T1-2 | unknow | N1 |
| TCGA-EM-A3AI | Cluster1 | >65 | FEMALE | Stage I-II | T1-2 | unknow | unknow |
| TCGA-EM-A3AJ | Cluster1 | <=65 | MALE | Stage I-II | T3-4 | unknow | N1 |
| TCGA-EM-A3AL | Cluster1 | <=65 | FEMALE | Stage I-II | T1-2 | unknow | N0 |
| TCGA-EM-A3AN | Cluster1 | <=65 | FEMALE | Stage I-II | T1-2 | unknow | N1 |
| TCGA-EM-A3AP | Cluster1 | <=65 | FEMALE | Stage I-II | T1-2 | unknow | unknow |
| TCGA-EM-A3AR | Cluster1 | <=65 | MALE | Stage I-II | T1-2 | unknow | N1 |
| TCGA-EM-A3FL | Cluster1 | <=65 | FEMALE | Stage I-II | T1-2 | unknow | unknow |
| TCGA-EM-A3FN | Cluster1 | <=65 | FEMALE | Stage I-II | T3-4 | unknow | N0 |
| TCGA-EM-A3FP | Cluster1 | >65 | FEMALE | Stage I-II | T1-2 | unknow | N0 |
| TCGA-EM-A3FR | Cluster1 | <=65 | FEMALE | Stage III-IV | T1-2 | unknow | N1 |
| TCGA-EM-A3O6 | Cluster1 | <=65 | FEMALE | Stage I-II | T1-2 | unknow | unknow |
| TCGA-EM-A3OA | Cluster1 | <=65 | FEMALE | Stage I-II | T1-2 | unknow | N0 |
| TCGA-EM-A3OB | Cluster1 | <=65 | FEMALE | Stage I-II | T1-2 | unknow | N0 |
| TCGA-EM-A3SY | Cluster1 | <=65 | MALE | Stage I-II | T1-2 | unknow | unknow |
| TCGA-EM-A4FH | Cluster1 | >65 | MALE | Stage III-IV | T3-4 | unknow | unknow |
| TCGA-EM-A4FK | Cluster1 | <=65 | FEMALE | Stage I-II | T1-2 | unknow | unknow |
| TCGA-EM-A4FO | Cluster1 | >65 | MALE | Stage I-II | T1-2 | unknow | N0 |
| TCGA-EM-A4FU | Cluster1 | <=65 | MALE | Stage I-II | T3-4 | unknow | unknow |
| TCGA-ET-A25G | Cluster1 | <=65 | FEMALE | Stage III-IV | T3-4 | unknow | N0 |
| TCGA-ET-A25I | Cluster1 | <=65 | FEMALE | Stage I-II | T1-2 | unknow | N0 |
| TCGA-ET-A2N3 | Cluster1 | <=65 | FEMALE | Stage I-II | T1-2 | unknow | unknow |
| TCGA-ET-A2N5 | Cluster1 | <=65 | FEMALE | Stage III-IV | T3-4 | unknow | unknow |
| TCGA-ET-A39I | Cluster1 | >65 | FEMALE | Stage I-II | T1-2 | unknow | unknow |
| TCGA-ET-A39L | Cluster1 | <=65 | FEMALE | Stage I-II | T3-4 | unknow | N0 |
| TCGA-ET-A39N | Cluster1 | <=65 | FEMALE | Stage I-II | T3-4 | unknow | unknow |
| TCGA-ET-A3BV | Cluster1 | <=65 | FEMALE | Stage III-IV | T1-2 | unknow | N1 |
| TCGA-ET-A3DO | Cluster1 | <=65 | FEMALE | Stage I-II | T1-2 | unknow | unknow |
| TCGA-ET-A3DQ | Cluster1 | <=65 | FEMALE | Stage I-II | T1-2 | unknow | N0 |
| TCGA-ET-A3DR | Cluster1 | <=65 | FEMALE | Stage I-II | T1-2 | unknow | N1 |
| TCGA-ET-A3DS | Cluster1 | <=65 | FEMALE | Stage I-II | T1-2 | unknow | N0 |
| TCGA-ET-A3DU | Cluster1 | <=65 | FEMALE | Stage I-II | T1-2 | unknow | N1 |
| TCGA-ET-A3DV | Cluster1 | >65 | FEMALE | Stage I-II | T3-4 | unknow | N0 |
| TCGA-ET-A40P | Cluster1 | <=65 | FEMALE | Stage I-II | T1-2 | unknow | N0 |
| TCGA-ET-A40S | Cluster1 | <=65 | MALE | Stage I-II | T1-2 | unknow | N0 |
| TCGA-ET-A4KQ | Cluster1 | <=65 | FEMALE | Stage I-II | T1-2 | unknow | unknow |
| TCGA-FE-A239 | Cluster1 | >65 | MALE | Stage III-IV | T3-4 | unknow | N0 |
| TCGA-FK-A3SD | Cluster1 | <=65 | FEMALE | Stage I-II | T1-2 | M0 | N0 |
| TCGA-FY-A2QD | Cluster1 | <=65 | FEMALE | unknow | T1-2 | unknow | N0 |
| TCGA-FY-A3I5 | Cluster1 | <=65 | FEMALE | Stage III-IV | T3-4 | unknow | N0 |
| TCGA-FY-A3NM | Cluster1 | <=65 | FEMALE | Stage III-IV | T3-4 | unknow | N0 |
| TCGA-FY-A3NP | Cluster1 | >65 | MALE | Stage I-II | T1-2 | M0 | N0 |
| TCGA-FY-A3R6 | Cluster1 | <=65 | FEMALE | Stage III-IV | T1-2 | unknow | N1 |
| TCGA-FY-A3R7 | Cluster1 | <=65 | FEMALE | Stage III-IV | T3-4 | unknow | N1 |
| TCGA-FY-A3R9 | Cluster1 | >65 | FEMALE | Stage I-II | T1-2 | unknow | N0 |
| TCGA-FY-A3TY | Cluster1 | <=65 | FEMALE | Stage III-IV | T3-4 | unknow | N0 |
| TCGA-FY-A3W9 | Cluster1 | >65 | FEMALE | Stage I-II | T1-2 | unknow | N0 |
| TCGA-FY-A3WA | Cluster1 | <=65 | FEMALE | Stage I-II | T1-2 | unknow | unknow |
| TCGA-FY-A40M | Cluster1 | <=65 | FEMALE | Stage I-II | T1-2 | unknow | N0 |
| TCGA-FY-A4B0 | Cluster1 | >65 | MALE | Stage I-II | T1-2 | unknow | unknow |
| TCGA-H2-A2K9 | Cluster1 | <=65 | MALE | Stage I-II | T1-2 | unknow | N1 |
| TCGA-J8-A3O0 | Cluster1 | <=65 | MALE | Stage I-II | T1-2 | M0 | N0 |
| TCGA-J8-A3O1 | Cluster1 | <=65 | FEMALE | Stage I-II | T3-4 | M0 | N1b |
| TCGA-J8-A3YD | Cluster1 | <=65 | FEMALE | Stage III-IV | T1-2 | M0 | N1a |
| TCGA-KS-A41I | Cluster1 | <=65 | FEMALE | Stage I-II | T1-2 | M0 | N0 |
| TCGA-KS-A41L | Cluster1 | <=65 | FEMALE | Stage I-II | T1-2 | M0 | N0 |
| TCGA-KS-A4ID | Cluster1 | <=65 | FEMALE | Stage I-II | T1-2 | M0 | N0 |
| TCGA-L6-A4EU | Cluster1 | <=65 | FEMALE | Stage III-IV | T3-4 | M0 | N1a |
| TCGA-MK-A84Z | Cluster1 | >65 | MALE | Stage III-IV | T3-4 | unknow | unknow |
| TCGA-QD-A8IV | Cluster1 | <=65 | FEMALE | Stage III-IV | T3-4 | unknow | N1 |
| TCGA-BJ-A0Z2 | Cluster2 | <=65 | MALE | Stage III-IV | T1-2 | M1 | N0 |
| TCGA-BJ-A0Z3 | Cluster2 | <=65 | FEMALE | Stage I-II | T1-2 | M0 | N0 |
| TCGA-BJ-A0Z5 | Cluster2 | <=65 | MALE | Stage III-IV | T3-4 | M0 | N1a |
| TCGA-BJ-A0ZB | Cluster2 | >65 | MALE | Stage III-IV | T3-4 | M0 | N1b |
| TCGA-BJ-A0ZH | Cluster2 | <=65 | FEMALE | Stage III-IV | T1-2 | unknow | N1 |
| TCGA-BJ-A18Z | Cluster2 | <=65 | MALE | Stage III-IV | T3-4 | unknow | N1 |
| TCGA-BJ-A28R | Cluster2 | <=65 | FEMALE | Stage I-II | T1-2 | M0 | N0 |
| TCGA-BJ-A28X | Cluster2 | <=65 | FEMALE | Stage I-II | T3-4 | M0 | N1a |
| TCGA-BJ-A290 | Cluster2 | >65 | MALE | Stage III-IV | T3-4 | M0 | N1a |
| TCGA-BJ-A2N8 | Cluster2 | <=65 | FEMALE | Stage I-II | T1-2 | M0 | N0 |
| TCGA-BJ-A2NA | Cluster2 | >65 | MALE | Stage III-IV | T3-4 | M0 | N0 |
| TCGA-BJ-A3EZ | Cluster2 | <=65 | MALE | Stage III-IV | T3-4 | M0 | N1b |
| TCGA-BJ-A3PR | Cluster2 | >65 | FEMALE | Stage I-II | T1-2 | M0 | N0 |
| TCGA-BJ-A3PU | Cluster2 | <=65 | MALE | Stage III-IV | T3-4 | M0 | N1a |
| TCGA-BJ-A45I | Cluster2 | <=65 | FEMALE | Stage III-IV | T3-4 | M0 | N0 |
| TCGA-BJ-A45J | Cluster2 | <=65 | FEMALE | Stage I-II | T1-2 | M0 | N1a |
| TCGA-BJ-A4O8 | Cluster2 | <=65 | MALE | Stage III-IV | T3-4 | M0 | N1a |
| TCGA-BJ-A4O9 | Cluster2 | <=65 | FEMALE | Stage I-II | T1-2 | M0 | N0 |
| TCGA-CE-A13K | Cluster2 | <=65 | FEMALE | Stage I-II | T3-4 | M0 | N1 |
| TCGA-CE-A3MD | Cluster2 | <=65 | MALE | Stage I-II | T1-2 | M0 | N1 |
| TCGA-CE-A3ME | Cluster2 | <=65 | FEMALE | Stage I-II | T1-2 | M0 | N0 |
| TCGA-CE-A481 | Cluster2 | <=65 | FEMALE | Stage I-II | T1-2 | M0 | N0 |
| TCGA-CE-A483 | Cluster2 | <=65 | FEMALE | Stage I-II | T1-2 | M0 | N1 |
| TCGA-CE-A484 | Cluster2 | <=65 | FEMALE | Stage I-II | T1-2 | M0 | N1 |
| TCGA-CE-A485 | Cluster2 | <=65 | MALE | Stage I-II | T1-2 | M0 | N1 |
| TCGA-DE-A0XZ | Cluster2 | <=65 | FEMALE | Stage III-IV | T1-2 | M0 | N1a |
| TCGA-DE-A0Y2 | Cluster2 | <=65 | FEMALE | Stage I-II | T1-2 | M0 | N1a |
| TCGA-DE-A0Y3 | Cluster2 | <=65 | FEMALE | Stage III-IV | T3-4 | M1 | N1b |
| TCGA-DE-A3KN | Cluster2 | <=65 | FEMALE | Stage III-IV | T3-4 | M0 | N1b |
| TCGA-DE-A4M8 | Cluster2 | <=65 | FEMALE | Stage III-IV | T1-2 | M0 | N1 |
| TCGA-DE-A4MC | Cluster2 | <=65 | FEMALE | Stage I-II | T3-4 | M0 | N1b |
| TCGA-DE-A4MD | Cluster2 | >65 | MALE | Stage III-IV | T3-4 | M0 | N1b |
| TCGA-DE-A69K | Cluster2 | <=65 | FEMALE | Stage III-IV | T3-4 | M0 | N0 |
| TCGA-DE-A7U5 | Cluster2 | <=65 | FEMALE | Stage I-II | T1-2 | M0 | N0 |
| TCGA-DJ-A13L | Cluster2 | >65 | MALE | Stage I-II | T1-2 | M0 | N0 |
| TCGA-DJ-A13O | Cluster2 | <=65 | MALE | Stage I-II | T1-2 | M0 | N0 |
| TCGA-DJ-A13P | Cluster2 | <=65 | FEMALE | Stage I-II | T1-2 | M0 | N0 |
| TCGA-DJ-A13T | Cluster2 | <=65 | FEMALE | Stage I-II | T1-2 | M0 | N0 |
| TCGA-DJ-A13U | Cluster2 | <=65 | MALE | Stage III-IV | T3-4 | M0 | N0 |
| TCGA-DJ-A13X | Cluster2 | <=65 | FEMALE | Stage III-IV | T3-4 | M0 | N0 |
| TCGA-DJ-A1QD | Cluster2 | <=65 | FEMALE | Stage I-II | T1-2 | M0 | N1b |
| TCGA-DJ-A1QE | Cluster2 | <=65 | FEMALE | Stage III-IV | T3-4 | M0 | N0 |
| TCGA-DJ-A1QF | Cluster2 | <=65 | FEMALE | Stage I-II | T1-2 | M0 | N0 |
| TCGA-DJ-A1QH | Cluster2 | <=65 | FEMALE | Stage III-IV | T3-4 | M0 | N1a |
| TCGA-DJ-A1QI | Cluster2 | <=65 | FEMALE | Stage I-II | T1-2 | M0 | N0 |
| TCGA-DJ-A1QN | Cluster2 | <=65 | FEMALE | Stage I-II | T1-2 | M0 | N0 |
| TCGA-DJ-A1QO | Cluster2 | >65 | MALE | Stage III-IV | T3-4 | M0 | N0 |
| TCGA-DJ-A1QQ | Cluster2 | <=65 | MALE | Stage I-II | T3-4 | M0 | N0 |
| TCGA-DJ-A2PN | Cluster2 | >65 | FEMALE | Stage I-II | T1-2 | M0 | N0 |
| TCGA-DJ-A2PO | Cluster2 | <=65 | MALE | Stage I-II | T1-2 | M0 | N0 |
| TCGA-DJ-A2PS | Cluster2 | <=65 | FEMALE | Stage I-II | T3-4 | M0 | N1a |
| TCGA-DJ-A2PT | Cluster2 | >65 | FEMALE | Stage III-IV | T3-4 | M0 | N0 |
| TCGA-DJ-A2PV | Cluster2 | <=65 | FEMALE | Stage I-II | T1-2 | M0 | N0 |
| TCGA-DJ-A2PW | Cluster2 | <=65 | MALE | Stage III-IV | T3-4 | M0 | N1a |
| TCGA-DJ-A2PX | Cluster2 | <=65 | FEMALE | Stage I-II | T1-2 | M0 | N0 |
| TCGA-DJ-A2PY | Cluster2 | <=65 | FEMALE | Stage III-IV | T3-4 | M0 | N1a |
| TCGA-DJ-A2PZ | Cluster2 | <=65 | MALE | Stage III-IV | T3-4 | M0 | N0 |
| TCGA-DJ-A2Q3 | Cluster2 | <=65 | FEMALE | Stage III-IV | T3-4 | M0 | N1a |
| TCGA-DJ-A2Q4 | Cluster2 | <=65 | MALE | Stage III-IV | T1-2 | M0 | N1a |
| TCGA-DJ-A2Q6 | Cluster2 | <=65 | FEMALE | Stage I-II | T1-2 | M0 | N1b |
| TCGA-DJ-A2Q7 | Cluster2 | <=65 | FEMALE | Stage III-IV | T1-2 | M0 | N1a |
| TCGA-DJ-A2QA | Cluster2 | <=65 | FEMALE | Stage III-IV | T3-4 | M0 | N0 |
| TCGA-DJ-A2QC | Cluster2 | >65 | FEMALE | Stage I-II | T1-2 | M0 | N0 |
| TCGA-DJ-A3UO | Cluster2 | <=65 | MALE | Stage III-IV | T3-4 | M0 | N1b |
| TCGA-DJ-A3UP | Cluster2 | <=65 | FEMALE | Stage I-II | T1-2 | M0 | N0 |
| TCGA-DJ-A3UQ | Cluster2 | <=65 | FEMALE | Stage III-IV | T3-4 | M0 | N1a |
| TCGA-DJ-A3UW | Cluster2 | <=65 | FEMALE | Stage I-II | T1-2 | M0 | N0 |
| TCGA-DJ-A3UX | Cluster2 | <=65 | FEMALE | Stage I-II | T1-2 | M0 | N0 |
| TCGA-DJ-A3UZ | Cluster2 | >65 | FEMALE | Stage III-IV | T3-4 | M0 | N0 |
| TCGA-DJ-A3V0 | Cluster2 | <=65 | MALE | Stage III-IV | T3-4 | M0 | N1b |
| TCGA-DJ-A3V3 | Cluster2 | <=65 | FEMALE | Stage I-II | T1-2 | M0 | N0 |
| TCGA-DJ-A3V4 | Cluster2 | <=65 | FEMALE | Stage III-IV | T3-4 | M0 | N1a |
| TCGA-DJ-A3V5 | Cluster2 | >65 | FEMALE | Stage III-IV | T3-4 | M0 | N0 |
| TCGA-DJ-A3V6 | Cluster2 | <=65 | MALE | Stage III-IV | T3-4 | M0 | N0 |
| TCGA-DJ-A3V7 | Cluster2 | <=65 | FEMALE | Stage III-IV | T3-4 | M0 | N0 |
| TCGA-DJ-A3VA | Cluster2 | <=65 | FEMALE | Stage I-II | T1-2 | M0 | N1a |
| TCGA-DJ-A3VB | Cluster2 | <=65 | MALE | Stage III-IV | T3-4 | M0 | N1a |
| TCGA-DJ-A3VI | Cluster2 | <=65 | FEMALE | Stage III-IV | T1-2 | M0 | N1a |
| TCGA-DJ-A3VK | Cluster2 | <=65 | MALE | Stage III-IV | T3-4 | M0 | N0 |
| TCGA-DJ-A4UP | Cluster2 | <=65 | FEMALE | Stage I-II | T1-2 | M0 | N1b |
| TCGA-DJ-A4UT | Cluster2 | <=65 | FEMALE | Stage I-II | T1-2 | M0 | N0 |
| TCGA-DJ-A4UW | Cluster2 | <=65 | FEMALE | Stage I-II | T1-2 | M0 | N1b |
| TCGA-DJ-A4V2 | Cluster2 | <=65 | FEMALE | Stage I-II | T1-2 | M0 | N0 |
| TCGA-DJ-A4V4 | Cluster2 | <=65 | FEMALE | Stage I-II | T1-2 | M0 | N0 |
| TCGA-DJ-A4V5 | Cluster2 | <=65 | MALE | Stage III-IV | T3-4 | M0 | N1b |
| TCGA-DO-A1K0 | Cluster2 | <=65 | FEMALE | Stage I-II | T3-4 | unknow | N1 |
| TCGA-E3-A3DY | Cluster2 | <=65 | MALE | Stage I-II | T3-4 | M0 | N1a |
| TCGA-E3-A3E0 | Cluster2 | <=65 | FEMALE | Stage I-II | T1-2 | unknow | N0 |
| TCGA-E3-A3E1 | Cluster2 | <=65 | FEMALE | Stage I-II | T1-2 | M0 | N0 |
| TCGA-E3-A3E2 | Cluster2 | <=65 | FEMALE | Stage I-II | T1-2 | unknow | N1 |
| TCGA-E3-A3E3 | Cluster2 | <=65 | FEMALE | Stage I-II | T1-2 | M0 | N0 |
| TCGA-E3-A3E5 | Cluster2 | <=65 | MALE | Stage III-IV | T3-4 | unknow | N1 |
| TCGA-E8-A242 | Cluster2 | <=65 | FEMALE | Stage III-IV | T3-4 | unknow | N0 |
| TCGA-E8-A2EA | Cluster2 | <=65 | FEMALE | Stage I-II | T1-2 | M0 | N0 |
| TCGA-E8-A2JQ | Cluster2 | <=65 | FEMALE | Stage I-II | T3-4 | M0 | N1a |
| TCGA-E8-A3X7 | Cluster2 | <=65 | FEMALE | Stage III-IV | T3-4 | M0 | N0 |
| TCGA-E8-A413 | Cluster2 | <=65 | FEMALE | Stage I-II | T1-2 | M0 | N0 |
| TCGA-E8-A415 | Cluster2 | <=65 | FEMALE | Stage I-II | T1-2 | M0 | N1b |
| TCGA-E8-A417 | Cluster2 | <=65 | FEMALE | Stage I-II | T1-2 | M0 | N1a |
| TCGA-E8-A418 | Cluster2 | >65 | FEMALE | Stage III-IV | T3-4 | M0 | N0 |
| TCGA-E8-A419 | Cluster2 | <=65 | FEMALE | Stage I-II | T1-2 | M0 | N1 |
| TCGA-E8-A432 | Cluster2 | <=65 | FEMALE | Stage I-II | T1-2 | M0 | N0 |
| TCGA-E8-A436 | Cluster2 | <=65 | FEMALE | Stage III-IV | T1-2 | M0 | N1b |
| TCGA-E8-A437 | Cluster2 | <=65 | FEMALE | Stage I-II | unknow | M0 | NX |
| TCGA-E8-A44K | Cluster2 | <=65 | FEMALE | Stage I-II | T1-2 | M0 | N0 |
| TCGA-EL-A3CM | Cluster2 | <=65 | FEMALE | Stage III-IV | T3-4 | M0 | N1b |
| TCGA-EL-A3CR | Cluster2 | >65 | FEMALE | Stage III-IV | T3-4 | M0 | N1b |
| TCGA-EL-A3CU | Cluster2 | >65 | FEMALE | Stage III-IV | T1-2 | M0 | N1a |
| TCGA-EL-A3D0 | Cluster2 | <=65 | MALE | Stage III-IV | T3-4 | M0 | N1b |
| TCGA-EL-A3D6 | Cluster2 | <=65 | FEMALE | Stage III-IV | T3-4 | M0 | N1b |
| TCGA-EL-A3GQ | Cluster2 | >65 | FEMALE | Stage I-II | T1-2 | M0 | N0 |
| TCGA-EL-A3GR | Cluster2 | <=65 | FEMALE | Stage I-II | T3-4 | M0 | NX |
| TCGA-EL-A3GS | Cluster2 | <=65 | FEMALE | Stage I-II | T3-4 | M0 | N1 |
| TCGA-EL-A3GU | Cluster2 | >65 | FEMALE | Stage III-IV | T3-4 | M0 | N1 |
| TCGA-EL-A3GV | Cluster2 | <=65 | FEMALE | Stage III-IV | T3-4 | M0 | N0 |
| TCGA-EL-A3GX | Cluster2 | <=65 | FEMALE | Stage I-II | T1-2 | M0 | N1 |
| TCGA-EL-A3GY | Cluster2 | <=65 | FEMALE | Stage I-II | T1-2 | M1 | N1 |
| TCGA-EL-A3H4 | Cluster2 | <=65 | FEMALE | Stage III-IV | T3-4 | M0 | N1 |
| TCGA-EL-A3H7 | Cluster2 | <=65 | FEMALE | Stage I-II | T3-4 | M0 | N1 |
| TCGA-EL-A3H8 | Cluster2 | <=65 | FEMALE | Stage I-II | T3-4 | M0 | N1 |
| TCGA-EL-A3MZ | Cluster2 | >65 | MALE | Stage III-IV | T3-4 | unknow | N1 |
| TCGA-EL-A3N2 | Cluster2 | <=65 | FEMALE | Stage I-II | T1-2 | M0 | N1 |
| TCGA-EL-A3N3 | Cluster2 | <=65 | FEMALE | Stage I-II | T3-4 | M0 | NX |
| TCGA-EL-A3T0 | Cluster2 | <=65 | FEMALE | Stage III-IV | T3-4 | M0 | N1 |
| TCGA-EL-A3T6 | Cluster2 | <=65 | FEMALE | Stage I-II | T3-4 | M0 | N0 |
| TCGA-EL-A3TA | Cluster2 | <=65 | MALE | Stage I-II | T1-2 | M0 | N0 |
| TCGA-EL-A3ZG | Cluster2 | <=65 | MALE | Stage I-II | T3-4 | M0 | N0 |
| TCGA-EL-A3ZH | Cluster2 | <=65 | FEMALE | Stage I-II | T3-4 | M0 | N1 |
| TCGA-EL-A3ZM | Cluster2 | <=65 | MALE | Stage III-IV | T3-4 | M0 | N1 |
| TCGA-EL-A3ZN | Cluster2 | <=65 | FEMALE | Stage I-II | T3-4 | M0 | N1 |
| TCGA-EL-A3ZP | Cluster2 | <=65 | MALE | Stage I-II | T3-4 | M0 | N1 |
| TCGA-EL-A3ZS | Cluster2 | <=65 | FEMALE | Stage I-II | T1-2 | M0 | N1 |
| TCGA-EL-A3ZT | Cluster2 | <=65 | MALE | Stage I-II | T3-4 | M0 | N0 |
| TCGA-EL-A4JW | Cluster2 | <=65 | FEMALE | Stage I-II | T1-2 | M0 | N0 |
| TCGA-EL-A4JZ | Cluster2 | <=65 | FEMALE | Stage III-IV | T3-4 | M0 | N1a |
| TCGA-EL-A4K0 | Cluster2 | <=65 | FEMALE | Stage I-II | T1-2 | M0 | N0 |
| TCGA-EL-A4K4 | Cluster2 | <=65 | FEMALE | Stage I-II | T3-4 | M0 | N1 |
| TCGA-EL-A4K6 | Cluster2 | >65 | MALE | Stage III-IV | T3-4 | unknow | N1 |
| TCGA-EL-A4K7 | Cluster2 | >65 | MALE | Stage III-IV | T1-2 | M0 | N1 |
| TCGA-EM-A1CS | Cluster2 | <=65 | FEMALE | Stage I-II | T1-2 | unknow | N0 |
| TCGA-EM-A1CT | Cluster2 | >65 | MALE | Stage III-IV | T1-2 | unknow | N1 |
| TCGA-EM-A1CV | Cluster2 | <=65 | FEMALE | Stage I-II | T1-2 | unknow | N0 |
| TCGA-EM-A22I | Cluster2 | <=65 | FEMALE | Stage III-IV | T3-4 | unknow | N0 |
| TCGA-EM-A22M | Cluster2 | <=65 | MALE | Stage I-II | T1-2 | unknow | N0 |
| TCGA-EM-A22O | Cluster2 | >65 | MALE | Stage III-IV | T3-4 | unknow | N1 |
| TCGA-EM-A22P | Cluster2 | <=65 | MALE | Stage III-IV | T1-2 | unknow | N1 |
| TCGA-EM-A2CN | Cluster2 | <=65 | MALE | Stage III-IV | T3-4 | M1 | N0 |
| TCGA-EM-A2CS | Cluster2 | <=65 | FEMALE | Stage III-IV | T3-4 | unknow | N1 |
| TCGA-EM-A2CU | Cluster2 | <=65 | FEMALE | Stage III-IV | T1-2 | unknow | N1 |
| TCGA-EM-A2OZ | Cluster2 | >65 | MALE | Stage I-II | T1-2 | unknow | N0 |
| TCGA-EM-A2P2 | Cluster2 | <=65 | MALE | Stage III-IV | T3-4 | unknow | N0 |
| TCGA-EM-A2P3 | Cluster2 | <=65 | FEMALE | Stage I-II | T1-2 | unknow | unknow |
| TCGA-EM-A3AK | Cluster2 | <=65 | FEMALE | Stage I-II | T1-2 | unknow | N0 |
| TCGA-EM-A3AO | Cluster2 | <=65 | MALE | Stage III-IV | T1-2 | unknow | N1 |
| TCGA-EM-A3FM | Cluster2 | <=65 | MALE | Stage III-IV | T1-2 | unknow | N1 |
| TCGA-EM-A3FO | Cluster2 | <=65 | MALE | Stage I-II | T3-4 | unknow | N0 |
| TCGA-EM-A3FQ | Cluster2 | <=65 | FEMALE | Stage I-II | T3-4 | unknow | N1 |
| TCGA-EM-A3O3 | Cluster2 | >65 | FEMALE | Stage III-IV | T1-2 | unknow | N1 |
| TCGA-EM-A3O8 | Cluster2 | <=65 | FEMALE | Stage I-II | T1-2 | unknow | N0 |
| TCGA-EM-A3ST | Cluster2 | <=65 | FEMALE | Stage III-IV | T3-4 | unknow | N0 |
| TCGA-EM-A3SU | Cluster2 | <=65 | FEMALE | Stage I-II | T1-2 | unknow | N1 |
| TCGA-EM-A3SX | Cluster2 | <=65 | FEMALE | Stage I-II | T3-4 | unknow | N1 |
| TCGA-EM-A3SZ | Cluster2 | <=65 | FEMALE | Stage I-II | T1-2 | unknow | N0 |
| TCGA-EM-A4FF | Cluster2 | <=65 | FEMALE | Stage I-II | T1-2 | unknow | N1 |
| TCGA-EM-A4FM | Cluster2 | <=65 | FEMALE | Stage III-IV | T3-4 | unknow | N1 |
| TCGA-EM-A4FN | Cluster2 | <=65 | FEMALE | Stage III-IV | T1-2 | unknow | N1 |
| TCGA-EM-A4FQ | Cluster2 | <=65 | FEMALE | Stage I-II | T1-2 | unknow | N1 |
| TCGA-EM-A4FR | Cluster2 | <=65 | MALE | Stage I-II | T3-4 | unknow | N1 |
| TCGA-EM-A4FV | Cluster2 | <=65 | FEMALE | Stage I-II | T1-2 | unknow | N0 |
| TCGA-EM-A4G1 | Cluster2 | <=65 | FEMALE | Stage I-II | T1-2 | unknow | N0 |
| TCGA-ET-A25K | Cluster2 | <=65 | FEMALE | Stage I-II | T1-2 | unknow | N1 |
| TCGA-ET-A25L | Cluster2 | <=65 | FEMALE | Stage III-IV | T3-4 | unknow | N1 |
| TCGA-ET-A25M | Cluster2 | <=65 | MALE | Stage I-II | T1-2 | unknow | N1 |
| TCGA-ET-A25N | Cluster2 | <=65 | FEMALE | Stage I-II | T1-2 | unknow | N1 |
| TCGA-ET-A25O | Cluster2 | <=65 | FEMALE | Stage I-II | T1-2 | unknow | N1 |
| TCGA-ET-A25P | Cluster2 | <=65 | FEMALE | Stage I-II | T1-2 | unknow | N0 |
| TCGA-ET-A25R | Cluster2 | <=65 | FEMALE | Stage III-IV | T3-4 | unknow | unknow |
| TCGA-ET-A2MX | Cluster2 | <=65 | MALE | Stage I-II | T1-2 | unknow | N1 |
| TCGA-ET-A2MY | Cluster2 | >65 | FEMALE | Stage I-II | T1-2 | unknow | unknow |
| TCGA-ET-A2MZ | Cluster2 | <=65 | MALE | Stage I-II | T1-2 | unknow | N0 |
| TCGA-ET-A2N4 | Cluster2 | <=65 | FEMALE | Stage III-IV | T3-4 | unknow | N0 |
| TCGA-ET-A39O | Cluster2 | <=65 | MALE | Stage I-II | T1-2 | unknow | N0 |
| TCGA-ET-A39P | Cluster2 | >65 | FEMALE | Stage III-IV | T3-4 | unknow | N0 |
| TCGA-ET-A39R | Cluster2 | <=65 | FEMALE | Stage I-II | T1-2 | unknow | N0 |
| TCGA-ET-A39T | Cluster2 | <=65 | FEMALE | Stage III-IV | T3-4 | unknow | N0 |
| TCGA-ET-A3BN | Cluster2 | <=65 | FEMALE | Stage I-II | T1-2 | unknow | unknow |
| TCGA-ET-A3BO | Cluster2 | <=65 | FEMALE | Stage I-II | T3-4 | unknow | N1 |
| TCGA-ET-A3BP | Cluster2 | <=65 | FEMALE | Stage I-II | T1-2 | unknow | N1 |
| TCGA-ET-A3BT | Cluster2 | <=65 | FEMALE | Stage III-IV | T1-2 | unknow | N1 |
| TCGA-ET-A3BX | Cluster2 | <=65 | MALE | Stage I-II | T3-4 | unknow | N1 |
| TCGA-ET-A3DW | Cluster2 | <=65 | MALE | Stage III-IV | T1-2 | unknow | N1 |
| TCGA-ET-A40Q | Cluster2 | <=65 | MALE | Stage I-II | T3-4 | unknow | N1 |
| TCGA-ET-A40R | Cluster2 | <=65 | FEMALE | Stage I-II | T1-2 | unknow | N1 |
| TCGA-ET-A40T | Cluster2 | <=65 | FEMALE | Stage I-II | T1-2 | unknow | N1 |
| TCGA-ET-A4KN | Cluster2 | <=65 | FEMALE | Stage III-IV | T3-4 | unknow | N0 |
| TCGA-FE-A22Z | Cluster2 | <=65 | FEMALE | Stage III-IV | T3-4 | unknow | N1 |
| TCGA-FE-A230 | Cluster2 | <=65 | FEMALE | Stage I-II | T3-4 | unknow | N1 |
| TCGA-FE-A231 | Cluster2 | >65 | MALE | Stage III-IV | T3-4 | unknow | N1 |
| TCGA-FE-A232 | Cluster2 | <=65 | FEMALE | Stage I-II | T3-4 | unknow | unknow |
| TCGA-FE-A233 | Cluster2 | <=65 | FEMALE | Stage I-II | T1-2 | unknow | unknow |
| TCGA-FE-A234 | Cluster2 | <=65 | FEMALE | Stage I-II | T1-2 | unknow | N1 |
| TCGA-FE-A236 | Cluster2 | <=65 | MALE | Stage I-II | T1-2 | unknow | N1 |
| TCGA-FE-A237 | Cluster2 | <=65 | FEMALE | Stage I-II | T3-4 | unknow | N1 |
| TCGA-FE-A238 | Cluster2 | <=65 | FEMALE | Stage I-II | T3-4 | unknow | N0 |
| TCGA-FE-A23A | Cluster2 | <=65 | FEMALE | Stage I-II | T1-2 | unknow | N0 |
| TCGA-FE-A3PA | Cluster2 | <=65 | MALE | Stage I-II | T3-4 | M1 | N0 |
| TCGA-FE-A3PB | Cluster2 | <=65 | FEMALE | Stage I-II | T3-4 | unknow | N1 |
| TCGA-FE-A3PC | Cluster2 | <=65 | FEMALE | Stage I-II | T3-4 | unknow | N1 |
| TCGA-FE-A3PD | Cluster2 | <=65 | FEMALE | Stage I-II | T1-2 | unknow | N0 |
| TCGA-FK-A3S3 | Cluster2 | <=65 | FEMALE | Stage I-II | T3-4 | unknow | N1 |
| TCGA-FK-A3SE | Cluster2 | <=65 | FEMALE | Stage I-II | T1-2 | unknow | N1 |
| TCGA-FK-A3SG | Cluster2 | <=65 | FEMALE | Stage I-II | T1-2 | unknow | N1 |
| TCGA-FK-A4UB | Cluster2 | <=65 | MALE | Stage III-IV | T3-4 | unknow | N1 |
| TCGA-FY-A3BL | Cluster2 | <=65 | MALE | Stage I-II | T1-2 | M0 | NX |
| TCGA-FY-A3I4 | Cluster2 | <=65 | FEMALE | Stage I-II | T1-2 | M0 | N0 |
| TCGA-FY-A3NN | Cluster2 | <=65 | FEMALE | Stage III-IV | T1-2 | unknow | N1 |
| TCGA-FY-A3R8 | Cluster2 | <=65 | FEMALE | Stage III-IV | T3-4 | unknow | unknow |
| TCGA-FY-A3RA | Cluster2 | <=65 | FEMALE | Stage I-II | T1-2 | unknow | unknow |
| TCGA-FY-A3YR | Cluster2 | <=65 | FEMALE | Stage III-IV | T3-4 | unknow | N1 |
| TCGA-FY-A40L | Cluster2 | <=65 | FEMALE | Stage III-IV | T3-4 | unknow | N1 |
| TCGA-FY-A40N | Cluster2 | <=65 | FEMALE | Stage I-II | T1-2 | unknow | N0 |
| TCGA-FY-A4B3 | Cluster2 | <=65 | MALE | Stage III-IV | T3-4 | unknow | N1 |
| TCGA-FY-A4B4 | Cluster2 | <=65 | FEMALE | Stage III-IV | T1-2 | unknow | N1 |
| TCGA-FY-A76V | Cluster2 | <=65 | MALE | Stage I-II | T1-2 | unknow | unknow |
| TCGA-GE-A2C6 | Cluster2 | <=65 | FEMALE | Stage I-II | T1-2 | unknow | N1 |
| TCGA-H2-A26U | Cluster2 | <=65 | FEMALE | Stage III-IV | T3-4 | unknow | N0 |
| TCGA-H2-A3RH | Cluster2 | <=65 | FEMALE | Stage I-II | T1-2 | M0 | N0 |
| TCGA-H2-A3RI | Cluster2 | <=65 | FEMALE | Stage I-II | T3-4 | unknow | N0 |
| TCGA-H2-A421 | Cluster2 | <=65 | FEMALE | Stage I-II | T1-2 | M0 | N1a |
| TCGA-H2-A422 | Cluster2 | <=65 | FEMALE | Stage I-II | T1-2 | unknow | N0 |
| TCGA-IM-A3EB | Cluster2 | <=65 | FEMALE | Stage I-II | T3-4 | unknow | N1 |
| TCGA-IM-A3ED | Cluster2 | <=65 | FEMALE | Stage I-II | T1-2 | unknow | N0 |
| TCGA-IM-A3U2 | Cluster2 | >65 | FEMALE | Stage III-IV | T3-4 | unknow | N1 |
| TCGA-IM-A3U3 | Cluster2 | <=65 | FEMALE | Stage I-II | T1-2 | unknow | N0 |
| TCGA-IM-A41Y | Cluster2 | <=65 | FEMALE | Stage I-II | T3-4 | unknow | N1 |
| TCGA-IM-A41Z | Cluster2 | <=65 | FEMALE | Stage I-II | T1-2 | unknow | N0 |
| TCGA-IM-A420 | Cluster2 | <=65 | FEMALE | Stage I-II | T1-2 | unknow | N1 |
| TCGA-IM-A4EB | Cluster2 | <=65 | MALE | Stage I-II | T3-4 | unknow | N1 |
| TCGA-J8-A3NZ | Cluster2 | <=65 | FEMALE | Stage III-IV | T3-4 | M0 | N1a |
| TCGA-J8-A3YE | Cluster2 | <=65 | FEMALE | Stage I-II | T3-4 | M0 | N1 |
| TCGA-J8-A3YF | Cluster2 | >65 | MALE | Stage III-IV | T1-2 | M0 | N1 |
| TCGA-J8-A3YG | Cluster2 | <=65 | FEMALE | Stage III-IV | T1-2 | M0 | N1a |
| TCGA-J8-A4HW | Cluster2 | <=65 | FEMALE | Stage III-IV | T1-2 | M0 | N1 |
| TCGA-J8-A4HY | Cluster2 | >65 | FEMALE | Stage III-IV | T3-4 | unknow | N1 |
| TCGA-KS-A4I3 | Cluster2 | <=65 | MALE | Stage I-II | T3-4 | M0 | N1 |
| TCGA-KS-A4I5 | Cluster2 | <=65 | FEMALE | Stage III-IV | T1-2 | M0 | N1b |
| TCGA-KS-A4I9 | Cluster2 | <=65 | FEMALE | Stage I-II | T1-2 | M0 | N0 |
| TCGA-KS-A4IB | Cluster2 | <=65 | FEMALE | Stage I-II | T1-2 | M0 | N1 |
| TCGA-MK-A4N6 | Cluster2 | <=65 | MALE | Stage I-II | T3-4 | M0 | N1b |
| TCGA-BJ-A45H | Cluster3 | <=65 | MALE | Stage III-IV | T3-4 | M0 | N0 |
| TCGA-CE-A482 | Cluster3 | <=65 | FEMALE | Stage I-II | T1-2 | M0 | N1 |
| TCGA-DE-A4MA | Cluster3 | <=65 | FEMALE | Stage III-IV | T3-4 | M0 | N1a |
| TCGA-DE-A69J | Cluster3 | <=65 | FEMALE | Stage I-II | T1-2 | M0 | NX |
| TCGA-DJ-A13V | Cluster3 | <=65 | FEMALE | Stage I-II | T3-4 | M0 | N1a |
| TCGA-DJ-A2PQ | Cluster3 | <=65 | MALE | Stage I-II | T1-2 | M0 | N1a |
| TCGA-DJ-A2PR | Cluster3 | <=65 | MALE | Stage I-II | T3-4 | M0 | N1a |
| TCGA-DJ-A2PU | Cluster3 | <=65 | FEMALE | Stage I-II | T1-2 | M0 | N0 |
| TCGA-DJ-A2Q5 | Cluster3 | <=65 | MALE | Stage III-IV | T1-2 | M0 | N1b |
| TCGA-DJ-A3UK | Cluster3 | <=65 | FEMALE | Stage I-II | T1-2 | M0 | N0 |
| TCGA-DJ-A3UM | Cluster3 | <=65 | FEMALE | Stage I-II | T1-2 | M0 | N0 |
| TCGA-DJ-A3UN | Cluster3 | <=65 | FEMALE | Stage I-II | T1-2 | M0 | N0 |
| TCGA-DJ-A3UR | Cluster3 | <=65 | FEMALE | Stage III-IV | T3-4 | M0 | N1a |
| TCGA-DJ-A3UU | Cluster3 | <=65 | FEMALE | Stage III-IV | T3-4 | M0 | N0 |
| TCGA-DJ-A3V2 | Cluster3 | <=65 | FEMALE | Stage I-II | T1-2 | M0 | N1a |
| TCGA-DJ-A3V8 | Cluster3 | <=65 | FEMALE | Stage I-II | T3-4 | M0 | N1b |
| TCGA-DJ-A3V9 | Cluster3 | <=65 | FEMALE | Stage III-IV | T1-2 | M0 | N1b |
| TCGA-DJ-A3VE | Cluster3 | <=65 | MALE | Stage I-II | T1-2 | M0 | N0 |
| TCGA-DJ-A3VJ | Cluster3 | <=65 | MALE | Stage I-II | T3-4 | M0 | N1a |
| TCGA-DJ-A4UL | Cluster3 | >65 | FEMALE | Stage I-II | T1-2 | M0 | N0 |
| TCGA-DO-A1JZ | Cluster3 | <=65 | FEMALE | Stage I-II | T1-2 | unknow | unknow |
| TCGA-E8-A433 | Cluster3 | <=65 | FEMALE | Stage I-II | T1-2 | M0 | N0 |
| TCGA-EL-A3CL | Cluster3 | >65 | FEMALE | Stage I-II | T1-2 | M0 | NX |
| TCGA-EL-A3CP | Cluster3 | <=65 | FEMALE | unknow | T1-2 | M0 | NX |
| TCGA-EL-A3D1 | Cluster3 | <=65 | MALE | Stage I-II | T1-2 | M0 | N0 |
| TCGA-EL-A3GZ | Cluster3 | <=65 | FEMALE | Stage I-II | T1-2 | M0 | N0 |
| TCGA-EL-A3MW | Cluster3 | <=65 | FEMALE | Stage I-II | T1-2 | M0 | N0 |
| TCGA-EL-A3MX | Cluster3 | >65 | FEMALE | Stage III-IV | T3-4 | M1 | N1 |
| TCGA-EL-A3T3 | Cluster3 | <=65 | MALE | Stage I-II | T1-2 | M0 | N0 |
| TCGA-EL-A3T7 | Cluster3 | <=65 | FEMALE | Stage I-II | T1-2 | M0 | N0 |
| TCGA-EL-A3T8 | Cluster3 | <=65 | MALE | Stage I-II | T1-2 | M0 | N0 |
| TCGA-EL-A3TB | Cluster3 | <=65 | FEMALE | Stage III-IV | T3-4 | M0 | N1a |
| TCGA-EL-A3ZL | Cluster3 | <=65 | FEMALE | Stage I-II | T1-2 | M0 | N1 |
| TCGA-EL-A3ZQ | Cluster3 | >65 | FEMALE | Stage I-II | T1-2 | M0 | N0 |
| TCGA-EL-A4K9 | Cluster3 | >65 | MALE | Stage I-II | T1-2 | M0 | N0 |
| TCGA-EL-A4KG | Cluster3 | <=65 | FEMALE | Stage I-II | T3-4 | M0 | N0 |
| TCGA-EL-A4KH | Cluster3 | <=65 | FEMALE | Stage I-II | T1-2 | M0 | N0 |
| TCGA-EL-A4KI | Cluster3 | <=65 | MALE | Stage III-IV | T3-4 | unknow | N0 |
| TCGA-EM-A1CU | Cluster3 | <=65 | MALE | Stage I-II | T3-4 | M0 | N1a |
| TCGA-EM-A2OX | Cluster3 | <=65 | MALE | Stage III-IV | T1-2 | unknow | N1 |
| TCGA-EM-A2P0 | Cluster3 | <=65 | MALE | Stage I-II | T1-2 | unknow | N1 |
| TCGA-EM-A3AQ | Cluster3 | >65 | FEMALE | Stage I-II | T1-2 | unknow | N0 |
| TCGA-EM-A3FJ | Cluster3 | <=65 | FEMALE | Stage I-II | T1-2 | unknow | N1 |
| TCGA-EM-A3FK | Cluster3 | <=65 | FEMALE | Stage I-II | T1-2 | unknow | N1 |
| TCGA-EM-A3O7 | Cluster3 | <=65 | FEMALE | Stage III-IV | T3-4 | unknow | unknow |
| TCGA-ET-A2N0 | Cluster3 | <=65 | FEMALE | Stage III-IV | T1-2 | unknow | N1 |
| TCGA-ET-A39J | Cluster3 | <=65 | FEMALE | Stage I-II | T1-2 | unknow | unknow |
| TCGA-ET-A39K | Cluster3 | <=65 | FEMALE | Stage III-IV | T3-4 | unknow | N1 |
| TCGA-ET-A39M | Cluster3 | <=65 | MALE | Stage I-II | T1-2 | unknow | unknow |
| TCGA-ET-A39S | Cluster3 | <=65 | FEMALE | Stage I-II | T1-2 | unknow | N0 |
| TCGA-ET-A3BQ | Cluster3 | <=65 | FEMALE | Stage I-II | T1-2 | unknow | N0 |
| TCGA-ET-A3BS | Cluster3 | <=65 | MALE | Stage I-II | T3-4 | unknow | N1 |
| TCGA-ET-A3BU | Cluster3 | <=65 | MALE | Stage I-II | T1-2 | unknow | N1 |
| TCGA-ET-A3BW | Cluster3 | <=65 | FEMALE | Stage I-II | T1-2 | unknow | unknow |
| TCGA-ET-A3DP | Cluster3 | <=65 | FEMALE | Stage I-II | T1-2 | unknow | unknow |
| TCGA-FE-A235 | Cluster3 | <=65 | FEMALE | Stage I-II | T1-2 | unknow | N1 |
| TCGA-FK-A3SB | Cluster3 | <=65 | FEMALE | Stage I-II | T3-4 | unknow | N1 |
| TCGA-FK-A3SH | Cluster3 | <=65 | FEMALE | Stage III-IV | T1-2 | unknow | N1 |
| TCGA-FY-A3ON | Cluster3 | <=65 | MALE | Stage I-II | T1-2 | unknow | N1 |
| TCGA-FY-A40K | Cluster3 | <=65 | FEMALE | Stage I-II | T1-2 | unknow | N0 |
| TCGA-J8-A3O2 | Cluster3 | <=65 | MALE | Stage I-II | T3-4 | M0 | N1b |
| TCGA-J8-A3YH | Cluster3 | <=65 | MALE | Stage I-II | T3-4 | M0 | N1b |
| TCGA-J8-A42S | Cluster3 | <=65 | MALE | Stage III-IV | T1-2 | unknow | N1 |
| TCGA-KS-A41F | Cluster3 | <=65 | FEMALE | Stage I-II | T1-2 | M0 | N1 |
| TCGA-KS-A41J | Cluster3 | <=65 | FEMALE | Stage I-II | T1-2 | M0 | N1 |
| TCGA-KS-A4I1 | Cluster3 | <=65 | FEMALE | Stage I-II | T1-2 | M0 | N0 |
| TCGA-KS-A4I7 | Cluster3 | <=65 | FEMALE | Stage I-II | T1-2 | M0 | N0 |
| TCGA-KS-A4IC | Cluster3 | <=65 | FEMALE | Stage III-IV | T1-2 | M0 | N1a |
| TCGA-L6-A4EP | Cluster3 | <=65 | FEMALE | Stage I-II | T1-2 | M0 | N0 |
| TCGA-L6-A4EQ | Cluster3 | <=65 | MALE | Stage III-IV | T1-2 | M0 | N1a |
| TCGA-L6-A4ET | Cluster3 | <=65 | FEMALE | Stage III-IV | T3-4 | M1 | NX |
| TCGA-MK-A4N7 | Cluster3 | <=65 | FEMALE | Stage I-II | T1-2 | M0 | N0 |
| TCGA-MK-A4N9 | Cluster3 | <=65 | FEMALE | Stage I-II | T3-4 | M0 | N1 |

**Table S6.** Correlation analysis of immune checkpoint genes (PD-1 and PD-L1) with m6A-associated lncRNAs.

| **ID** | **PD-1** | | **PD-L1** | |
| --- | --- | --- | --- | --- |
|  | **cor** | **P-value** | **cor** | **P-value** |
| A2M-AS1 | -0.03208 | 0.469795 | -0.34209 | 1.9E-15 |
| AC004540.1 | 0.00445 | 0.920143 | -0.06666 | 0.132762 |
| AC004825.2 | -0.02243 | 0.613242 | -0.3602 | 4.54E-17 |
| AC005034.5 | -0.08947 | 0.043415 | -0.42797 | 3.95E-24 |
| AC006213.1 | -0.17914 | 4.73E-05 | -0.41532 | 1.1E-22 |
| AC007365.1 | -0.04012 | 0.365888 | -0.22649 | 2.35E-07 |
| AC008555.1 | -0.06218 | 0.1609 | -0.08881 | 0.044996 |
| AC008738.2 | -0.06587 | 0.137396 | -0.31771 | 2.01E-13 |
| AC010980.2 | -0.03296 | 0.457658 | -0.24007 | 4.05E-08 |
| AC011700.1 | -0.0653 | 0.140865 | -0.34021 | 2.76E-15 |
| AC019080.1 | -0.10421 | 0.018568 | -0.19068 | 1.45E-05 |
| AC023510.2 | 0.11156 | 0.0117 | -0.31547 | 3.01E-13 |
| AC040160.1 | -0.14369 | 0.001138 | 0.029022 | 0.513148 |
| AC046143.1 | -0.12859 | 0.003626 | 0.06352 | 0.152026 |
| AC064807.1 | -0.03629 | 0.413447 | -0.24694 | 1.6E-08 |
| AC067750.1 | -0.12571 | 0.004466 | -0.30859 | 1.03E-12 |
| AC079848.1 | -0.16642 | 0.00016 | -0.16286 | 0.000221 |
| AC091965.1 | -0.08544 | 0.053828 | -0.20742 | 2.31E-06 |
| AC096920.1 | -0.07069 | 0.110816 | -0.15441 | 0.000466 |
| AC102953.2 | -0.04614 | 0.298323 | -0.12546 | 0.004546 |
| AC103957.2 | -0.04236 | 0.339674 | -0.25386 | 6.09E-09 |
| AC104825.1 | -0.07181 | 0.105271 | -0.52154 | 6.46E-37 |
| AC106820.4 | -0.0603 | 0.173937 | -0.33321 | 1.09E-14 |
| AC106820.5 | -0.11855 | 0.007358 | -0.08357 | 0.0593 |
| AC108472.1 | -0.08073 | 0.068503 | -0.25563 | 4.73E-09 |
| AC114956.1 | -0.06826 | 0.123653 | -0.2087 | 1.99E-06 |
| AC125807.2 | -0.04734 | 0.285974 | -0.14317 | 0.001187 |
| AC126773.4 | 0.003827 | 0.931303 | -0.17495 | 7.13E-05 |
| ADAMTS9-AS2 | -0.18196 | 3.57E-05 | 0.043627 | 0.325463 |
| AL023583.1 | -0.00026 | 0.995414 | -0.28038 | 1.15E-10 |
| AL139288.1 | 0.024763 | 0.576891 | -0.09183 | 0.038157 |
| AL359532.1 | -0.02826 | 0.524334 | -0.34379 | 1.35E-15 |
| AL512306.2 | 0.41112 | 3.21E-22 | 0.009409 | 0.832128 |
| AP000802.1 | -0.00954 | 0.82985 | -0.19083 | 1.43E-05 |
| AP001271.1 | -0.04152 | 0.349365 | -0.20059 | 4.99E-06 |
| AP001453.4 | -0.00717 | 0.87166 | -0.10997 | 0.012956 |
| CASC2 | -0.15191 | 0.000577 | -0.34612 | 8.46E-16 |
| DLGAP1-AS2 | -0.05119 | 0.248528 | -0.10977 | 0.013122 |
| DOCK9-DT | -0.13284 | 0.002648 | -0.06216 | 0.161003 |
| DPH6-DT | -0.08496 | 0.055183 | -0.17674 | 5.99E-05 |
| DYRK3-AS1 | -0.01541 | 0.72843 | -0.25319 | 6.69E-09 |
| EIF2AK3-DT | 0.029643 | 0.504174 | -0.41283 | 2.08E-22 |
| FAM182B | -0.11265 | 0.010901 | -0.25181 | 8.12E-09 |
| IQCH-AS1 | -0.08849 | 0.045791 | -0.37089 | 4.46E-18 |
| ITPR1-DT | -0.04893 | 0.270067 | -0.31544 | 3.03E-13 |
| LCMT1-AS1 | 0.017906 | 0.686642 | -0.32061 | 1.18E-13 |
| LIFR-AS1 | -0.11563 | 0.008958 | -0.2817 | 9.31E-11 |
| LINC00205 | -0.05043 | 0.25563 | -0.40682 | 9.49E-22 |
| LINC00667 | -0.00878 | 0.843197 | -0.09796 | 0.026959 |
| LINC01144 | -0.14313 | 0.00119 | -0.39424 | 2.06E-20 |
| LINC01184 | -0.19022 | 1.53E-05 | -0.33349 | 1.03E-14 |
| LINC01975 | -0.04396 | 0.321754 | -0.147 | 0.00087 |
| LINC02028 | -0.06715 | 0.129887 | -0.33244 | 1.26E-14 |
| LINC02447 | -0.0923 | 0.037184 | -0.42098 | 2.53E-23 |
| LINC02454 | -0.09913 | 0.025173 | 0.229387 | 1.63E-07 |
| LYPLAL1-DT | -0.12258 | 0.005574 | -0.17699 | 5.85E-05 |
| MIR181A2HG | -0.12563 | 0.00449 | -0.10716 | 0.015477 |
| MKLN1-AS | -0.09197 | 0.037866 | -0.04249 | 0.338275 |
| MRPS30-DT | -0.19714 | 7.28E-06 | -0.24521 | 2.03E-08 |
| PAX8-AS1 | -0.04348 | 0.327079 | -0.19855 | 6.24E-06 |
| PROX1-AS1 | -0.07319 | 0.098747 | -0.39622 | 1.28E-20 |
| SAP30-DT | -0.00189 | 0.966051 | -0.22343 | 3.43E-07 |
| SGMS1-AS1 | -0.17962 | 4.51E-05 | -0.40058 | 4.45E-21 |
| ST7-AS1 | -0.05466 | 0.217869 | -0.26802 | 7.71E-10 |
| STX18-AS1 | -0.07261 | 0.101457 | -0.1857 | 2.44E-05 |
| TBC1D8-AS1 | -0.07619 | 0.085621 | -0.33983 | 2.98E-15 |
| TMEM220-AS1 | -0.09857 | 0.026012 | -0.27403 | 3.09E-10 |
| TONSL-AS1 | -0.03992 | 0.368289 | -0.37481 | 1.86E-18 |
| UXT-AS1 | -0.12211 | 0.005758 | -0.11645 | 0.00848 |
| WDFY3-AS2 | -0.17915 | 4.73E-05 | -0.27868 | 1.5E-10 |
